# Specific G-quadruplex ligands modulate the alternative splicing of Bcl-X

**DOI:** 10.1093/nar/gkx1122

**Published:** 2017-11-16

**Authors:** Carika Weldon, Justine G Dacanay, Vijay Gokhale, Peda Venkat L Boddupally, Isabelle Behm-Ansmant, Glenn A Burley, Christiane Branlant, Laurence H Hurley, Cyril Dominguez, Ian C Eperon

**Affiliations:** 1 Leicester Institute of Structural & Chemical Biology and Department of Molecular & Cell Biology, University of Leicester, Leicester LE1 7RH, UK; 2 College of Pharmacy and College of Pharmacy and BIO5 Institute, University of Arizona, Tucson, AZ 85721, USA; 3 Fluoroorganic Division, CSIR-Indian Institute of Chemical Technology, Hyderabad, Telangana 500 007, India; 4 IMoPA (Ingénierie Moléculaire et Physiopathologie Articulaire), UMR 7365 CNRS-UL, Biopôle de l’Université de Lorraine, 9 Avenue de la Forêt de Haye, 54505 Vandoeuvre-lès-Nancy, France; 5 Department of Pure and Applied Chemistry, University of Strathclyde, Glasgow G1 1XL, UK; 6 Arizona Cancer Center, University of Arizona, Tucson, AZ 85724, USA

## Abstract

Sequences with the potential to form RNA G-quadruplexes (G4s) are common in mammalian introns, especially in the proximity of the 5′ splice site (5′SS). However, the difficulty of demonstrating that G4s form in pre-mRNA in functional conditions has meant that little is known about their effects or mechanisms of action. We have shown previously that two G4s form in Bcl-X pre-mRNA, one close to each of the two alternative 5′SS. If these G4s affect splicing but are in competition with other RNA structures or RNA binding proteins, then ligands that stabilize them would increase the proportion of Bcl-X pre-mRNA molecules in which either or both G4s had formed, shifting Bcl-X splicing. We show here that a restricted set of G4 ligands do affect splicing, that their activity and specificity are strongly dependent on their structures and that they act independently at the two splice sites. One of the ligands, the ellipticine GQC-05, antagonizes the major 5′SS that expresses the anti-apoptotic isoform of Bcl-X and activates the alternative 5′SS that expresses a pro-apoptotic isoform. We propose mechanisms that would account for these see-saw effects and suggest that these effects contribute to the ability of GQC-05 to induce apoptosis.

## INTRODUCTION

G-rich sequences were first identified as functional elements in pre-mRNA splicing when G-triplets were found to be enriched in short introns, where they stimulate splicing and, in particular, the use of adjacent upstream 5′ splice sites (5′SSs) (1). Subsequently, they have been shown to be enriched in introns of all sizes, especially near the 5′SS, where they are thought to buffer 5′SS sequences against the effects of mutations (2). G-rich elements have been shown to facilitate the recruitment of U1 and U11 snRNPs (3–5). G-tracts can be bound directly by hnRNPs F and H (6–9), and it is likely that these proteins participate in the recruitment of snRNPs (5). HnRNP H is also implicated when these sequences act in other contexts as silencers (10–12) or to improve the splicing of long introns via the juxtaposition of distant G-tracts (13).

When G-rich sequences are extensive, they have another potential property, that of folding into four-stranded quadruplex structures (G4s) (14–16). These are stacks in which each plane comprises four guanines connected in a ring by Hoogsteen base-pairing. Four G-triplets in reasonable proximity, therefore, might form a G4 structure with three such rings in a stack. These structures readily form *in vitro* and can be characterized by analysis of short RNA oligonucleotides (17,18). However, although sequences with the potential to form such structures have been shown to affect the splicing of a number of pre-mRNAs (19–27), it has been difficult to prove that the G4s form in the context of long RNA molecules in functional splicing conditions, where their formation might face competition with secondary structures or the binding of proteins (18,28). We have recently developed a new method based on the use of deazaguanine-substituted RNA (FOLDeR) by which we have shown that two G4s form in functional conditions in Bcl-X pre-mRNA in the G-rich tracts adjacent to the two alternative 5′SS of exon 2 (29).

The proportion of molecules with G4-forming potential that contain G4 structures at any one time is hard to estimate. For example, immunostaining assays have detected RNA G4s in the cytoplasm of cells (30), but chemical modification experiments have shown that G4s that could form *in vitro* were largely unfolded in cells (31). This was attributed to competition with RNA-binding proteins. Indeed, we have shown previously that an artificial enhancer comprising repeats of a binding site for SRSF1, GGA, is able to adopt any of three distinct states, a G4 or complexes with either hnRNP F/H or SRSF1, and we suggested that these states were in dynamic equilibrium (18). Similarly, we have shown that hnRNP F/H binding and G4 formation were mutually exclusive (9,32), and a silencer in intron 1 of proinsulin has been shown to be in equilibrium between a G4 and a stem-loop structure (23). This is a very important point. If G4s form in only a minority of candidate sequences at any one time but there is an equilibrium, then the proportion of RNA forming a G4 should be increased by the addition of G4-stabilizing ligands. This might lead to significant shifts in reactions sensitive to G4 formation. We have tested this using Bcl-X pre-mRNA, and show that well-known DNA G4-stabilizers produce diverse effects. This is indicative of these molecules engaging with defined molecular targets. Some of these shift splicing from the dominant anti-apoptotic Bcl-XL isoform to the pro-apoptotic Bcl-XS isoform, while others have no effect on splicing. The active molecules are all from the ellipticine or quindoline classes, with fused tetracyclic cores and show differential effects on the two alternative splice sites according to the core and specific substituent groups. We further demonstrate that the most effective molecule interacts with and acts via the G4-forming sequences of Bcl-X pre-mRNA, and a preliminary structure-activity comparison suggests that specific G4-binding molecules have the potential to control splice site selection by acting at a subset of G4 structures.

## MATERIALS AND METHODS

### Synthesis of ellipticine and quindoline analogs

The syntheses and analytical characterizations of the quindoline series and the ellipticine analogs GQC-05 GSA1112 and GSA1125 have been described (33,34). The synthesis and characterization of GSA-1113, GSA-1126, GSA-1133 and GSA-1135 is described in Supplementary Data.

### *In vitro* transcription and splicing of RNA

*In vitro* transcription and splicing were done as described (35) using nuclear extracts from Cilbiotech. In brief, pre-mRNA was transcribed by T7 RNA polymerase in the presence of GpppG (to form the 5′ cap) and [α-^32^P]GTP and purified on a denaturing gel. Splicing assays were done in 10 μl reactions by adding 1 μl of RNA to pre-aliquoted reaction mixtures in microtiter plates. Reactions were incubated for 2 h at 30°C, processed in the microtiter plate and analyzed by denaturing gel electrophoresis. After analysis by a phosphor imager, quantification of pre-mRNA and both mRNA products was done using OptiQuant software, and intensities were normalized to account for the number of guanines in each RNA species.

### Analysis of splicing complex formation

Splicing complex formation was done as described (36) using nuclear extracts from Cilbiotech. Samples were incubated for 0, 5, 10, 15, 20, 25 and 30 min, and then incubated with heparin at 0.8 mg/ml for 30 min at ambient temperature. An equal volume of loading dyes (50 mM Tris base, 50 mM glycine, 40% glycerol, xylene cyanol and bromophenol blue) was added and samples run on a 2% native LMP agarose gel (50 mM Tris base, 50 mM glycine) at 5 V/cm for 6 h at 4°C. The gel was then compressed, dried and exposed to a phosphorimager screen.

### RNase digestion and primer extension

Transcripts were digested with ribonucleases T1 (Roche), T2 (Ambion) or VI (Mobitech) and the sites of cleavage detected using primer extension, as described (29). The phosphor image was analyzed using SAFA (37) and significant changes calculated by using the logarithms of the following ratio or index to analyze each nucleotide position: [V1(10 μM GQC-05)/V1(no GQC-05)]/[T2(10 μM GQC-05)/T2(no GQC-05)]. The mean and standard deviation were calculated for the log(Index) values for all positions analyzed, and positions at which the log(Index) value deviated from the mean by more than two standard deviations were considered to have shown significant changes.

### Fluorescence assays

A total of 1 μM RNA and 20 μM GQC-05 were mixed in a Hellma fluorescence cuvette (Suprasil^®^ quartz, limit 200–2500 nm spectral range, pathlength 10 × 2 mm, chamber volume 100 μl) and fluorescence spectra were obtained on a FluoroMax-4 spectrofluorometer at room temperature with excitation at 321 nm.

### Treatment of cells with G4 ligands

HeLa cells at 70% confluency were incubated with potential G4-stabilizing reagents at 10 μM for 4 h. RNA was extracted and analyzed by reverse transcription-PCR, with 45 cycles of amplification, followed by agarose gel electrophoresis. The primers used were ATGGCAGCAGTAAAGCAAGCG (forward) and TCATTTCCGACTGAAGAGTGA (reverse).

## RESULTS

**Figure 1.**
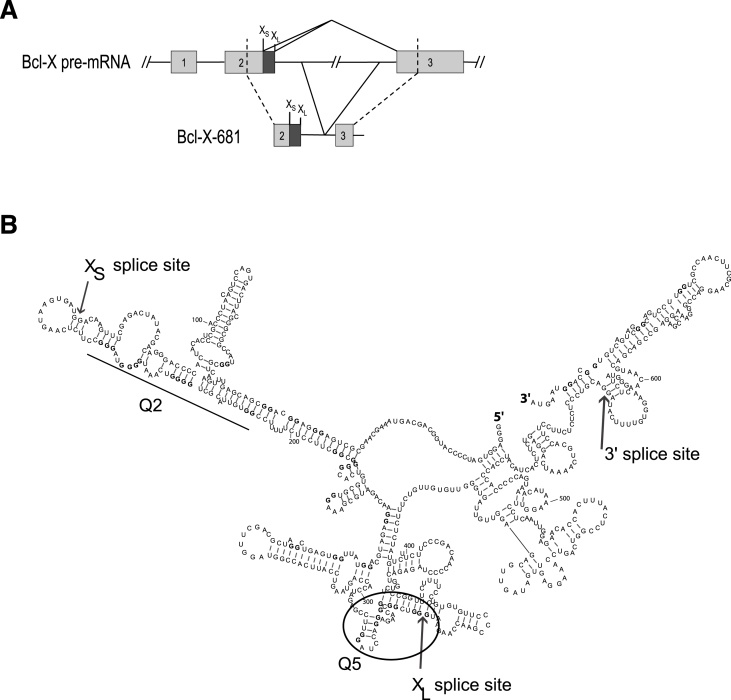
Organization and secondary structure of the Bcl-X-681 pre-mRNA. (**A**) Organization of the Bcl-X-681 transcript. X_L_ and X_S_ 5′ splice sites are indicated above the diagrams. (**B**) Experimentally determined secondary structure and location of the Q2 and Q5 G4s in Bcl-X-681 as previously determined (29).

**Figure 2.**
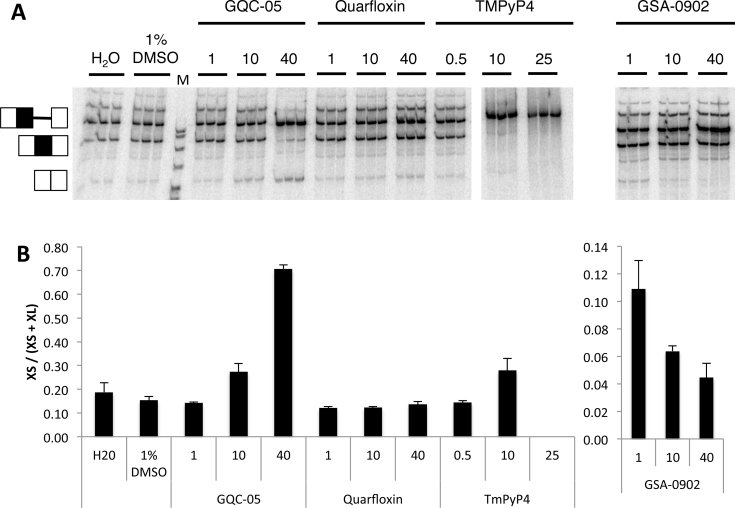
*In vitro* splicing of Bcl-X-681 in the presence of representative G4 ligands. (**A**) Splicing assays were done in triplicate with the designated ligands at the concentrations shown (μM). The ligands were dissolved in 10% DMSO, and controls included samples done in the presence of DMSO at the same final concentration of 1%. All the reactions were done at the same time and run on the same gel. (**B**) The proportion of the mRNA spliced to the X_S_ site in the reactions above, after correction for the different numbers of labeled nucleotides in the two different molecular species. The error bar shows the standard deviation.

Figure 1 shows a secondary structure of Bcl-X-681, an *in vitro* splicing substrate that comprises the 3′ half of Bcl-X exon 2, including both alternative 5′ splice sites (SSs), the adjacent 180 nucleotides (nts) of intron 2, the 3′ 61 nts of intron 2 and the 5′ part of exon 3 (29). Both 5′SS are used *in vitro* but, as with the endogenous gene, the downstream Bcl-X_L_ isoform predominates (29) (Figure 2, left column). We determined this structure previously using RNase footprinting and RNase H, showing that it forms in the presence or absence of nuclear extract; analysis of deazaguanosine-substituted pre-mRNA showed that, of six regions that might have had the potential to form G4s, only the regions marked Q2 and Q5 did so (29).

### Certain putative G4 ligands affect Bcl-X splice site selection

More than 800 molecules of various chemical classes have been described as G4-ligands (38), and a number of these affect splicing (18,22,24–26). We tested whether G4 ligands would affect the selection of 5′SS in Bcl-X by using molecules representing various chemical classes of ligands (Supplementary Figures S1–3; (33,39–45). As illustrated for four ligands in Figure 2, Quarfloxin, Thioflavin T and 360A had no detectable effects on splicing of Bcl-X-681 *in vitro*, whereas splicing was abolished by TMPyP4, Pyridostatin and Zn-DIGP (Figure 2; Supplementary Table S1 and Figure S1). In contrast, GQC-05 (NSC 338258; EPED3) and GSA-0902 had selective but opposite effects: GQC-05 increased the use of the X_S_ 5′SS while GSA-0902 decreased it (Figure 2; Supplementary Table S1 and Figures S2 and 3). The effect of GQC-05, an ellipticine derivative developed to bind a DNA G4 in the c-MYC promoter G4 (39), was particularly striking. At a concentration of 10 μM, GQC-05 increased usage of the X_S_ splice site with little apparent change in X_L_ site usage, whereas at 40 μM a greater increase in X_S_ splice site usage was accompanied by a decrease in X_L_ splice site. This resulted in the proportion of mRNA forming the X_S_ isoform rising from 15 to 80% (Figure 2B).

Since X_L_ splicing is normally the predominant pathway, the apparent change in X_L_ splice site usage in the presence of 40 μM GQC-05 involved a higher proportion of molecules of pre-mRNA than did the increase in X_S_ splicing. The effect could be due to a decrease in the efficiency of splicing from the X_L_ site or an increased level of degradation of the spliced X_L_ mRNA. To distinguish between these alternatives, we analyzed the effects of GQC-05 on the assembly of spliceosomes on Bcl-X-681. Native gel electrophoresis of samples taken from splicing reaction mixtures at various time intervals showed that spliceosome formation was reduced (Supplementary Figure S4). This is consistent with the suggestion that GQC-05 inhibits the major (X_L_) splicing pathway.

### Structural features of individual G4 ligands determine the target site and effects

**Figure 3.**
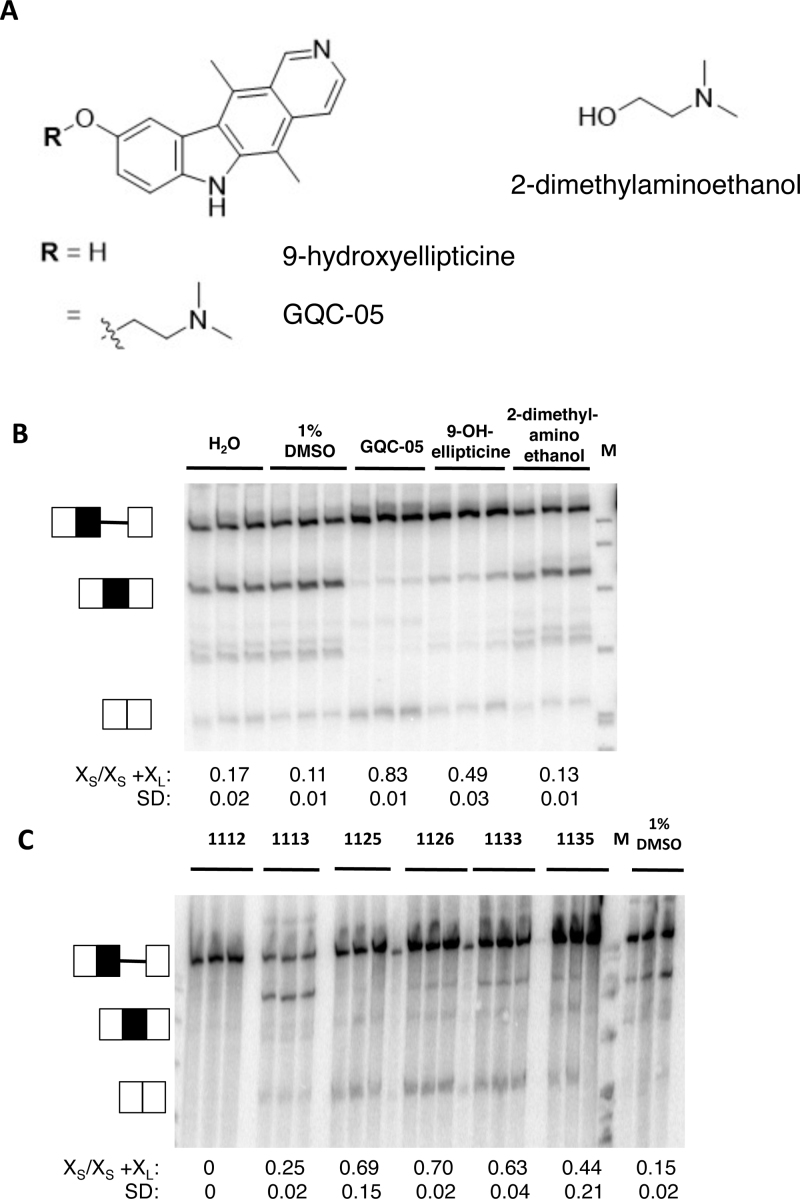
*In vitro* splicing of Bcl-X-681 in the presence of ellipticines. (**A**) Diagrams showing the core and tail of GQC-05. (**B**) Splicing assays done *in vitro* in triplicate with GQC-05 and the separate core and tail. The proportion of the mRNA spliced to the X_S_ site is shown below the image, together with the standard deviation. M, size markers. (**C**) Splicing assays done *in vitro* with other ellipticines (Supplementary Figure S2).

The opposite effects of GQC-05, an ellipticine derivative and GSA-0902, a quindoline derivative, were remarkable, given the similarity of their fused tetracyclic cores (Supplementary Figures S2 and 3). To determine the basis for this discrimination, we tested a range of analogs of GQC-05. GQC-05 can be divided into its tetracyclic core and the exocyclic tertiary amine tail, which will be protonated under physiological conditions (Figure 3A). Addition of 9-hydroxyellipticine sufficed to inhibit the downstream X_L_ 5′SS, but it did not stimulate use of the X_S_ 5′SS, whereas the tertiary amine tail alone had no effect (Figure 3B). The effect of the 9-hydroxyellipticine is consistent with independent effects of GQC-05 at the two 5′SS, indicating that up-regulation of the X_S_ site is not merely a consequence of downregulation of the X_L_ site.

Analysis of a range of other ellipticine derivatives (Supplementary Figure S2) revealed some general principles. Increasing the steric bulk of the tertiary amine was tolerated, as GSA-1125, GSA-1126, GSA-1133 and GSA-1135 showed the same activities as GQC-05, albeit to a lesser extent (Figure 3C). Unlike the 9-hydroxyellipticine alone, they stimulated use of the X_S_ 5′SS. GSA-1112 inhibited both sites, indicating that functionalization of the indole N-H (R^2^, position 6) prevents stimulation of the X_S_ 5′SS, but GSA-1113 had little effect on either site, from which we infer that when R^2^ is a tertiary amine it blocks the effect on the X_S_ site and that when R^1^ is an ether it prevents the inhibition of the X_L_ 5′SS. In summary, it appears that inhibition of the X_L_ 5′SS requires the ellipticine core without an ether at position 9 and that stimulation of the X_S_ 5′SS occurs with a positively charged tertiary amine at position 9 and an unfunctionalized indole NH in position 6.

We have examined a similar set of quindolines (Supplementary Figures S3 and 5; Supplementary Table S2). None of these greatly affected the X_L_ 5′SS, from which we infer that the tetracyclic ellipticine core structure is important for its inhibition. The X_S_ 5′SS was slightly stimulated by quindoline itself and slightly more strongly by SYUIQ-5, which has a tertiary amine on ring position 11. It is possible that this plays a role equivalent to the tertiary amine on position 9 of the ellipticines. However, the presence of piperazine rings at position 11 prevented any stimulation of the X_S_ 5′SS. Indeed, GSA-0902 and GSA-0820 inhibited the site. These results confirm that the G4 ligands act independently at the two sites and that specific features of the ligands are required for these effects; the shift in splicing induced by GQC-05 is not the result of binding by a generic G4 ligand.

**Figure 4.**
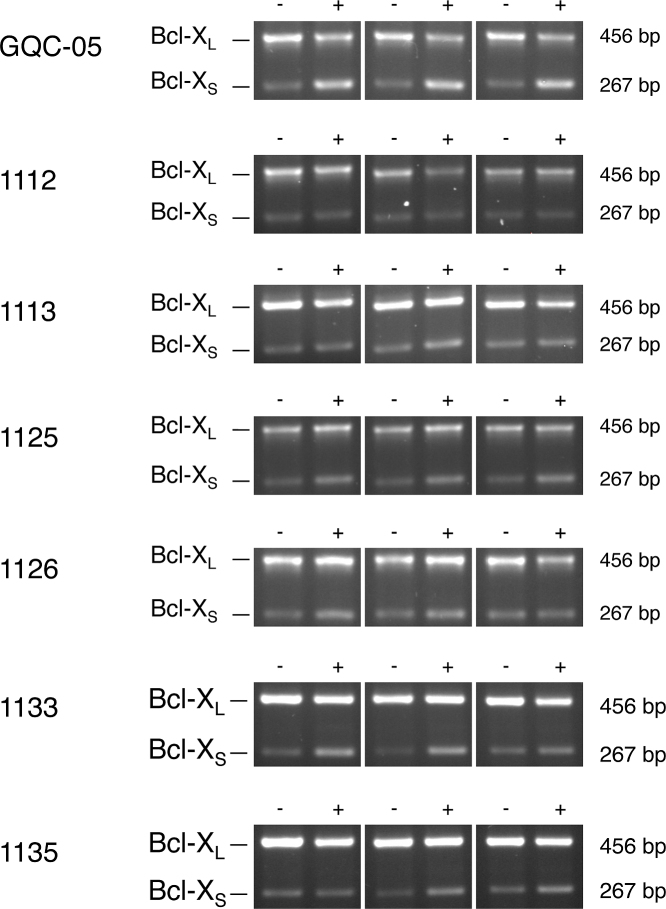
Effects of GQC-05 and other ellipticines on splicing of endogenous Bcl-X transcripts in HeLa cells. The cells were incubated with the compound at 10 μM for 4 h, followed by isolation of the RNA and amplification by reverse transcriptase-PCR. The products were separated by agarose gel electrophoresis and detected by ethidium bromide. The experiments were done in triplicate. Reactions done in parallel with reaction mixtures lacking reverse transcriptase were blank and have been omitted.

To test whether the effects of these molecules were replicated in cells, HeLa cells were incubated with these G4 ligands at 10 μM for 4 h and splicing of the endogenous Bcl-X was analyzed by RT-PCR. The results showed that GQC-05 produced a clear shift toward Bcl-X_S_ (Figure 4 and Supplementary Table S3). GSA-1125 produced a weaker shift, and the effects of GSA-1133, GSA-1135 and GSA-1126 were weaker still. As might have been expected from the *in vitro* results, GSA-1112 and GSA-1113 had little or no effect. The other G4 ligands tested produced little or no effect (Supplementary Figure S6 and Table S3). In the cases of Quarfloxin, Thioflavin T, 360A and the quindolines, these results were essentially the same as with the *in vitro* assays. However, there was no evidence of the inhibition of splicing seen *in vitro* with TMPyP4, Pyridostatin and Zn-DIGP.

Since GQC-05 showed a much stronger effect than any of the other candidates, further work was done with this ellipticine.

### GQC-05 interacts with the G4 regions of Bcl-X pre-mRNA

**Figure 5.**
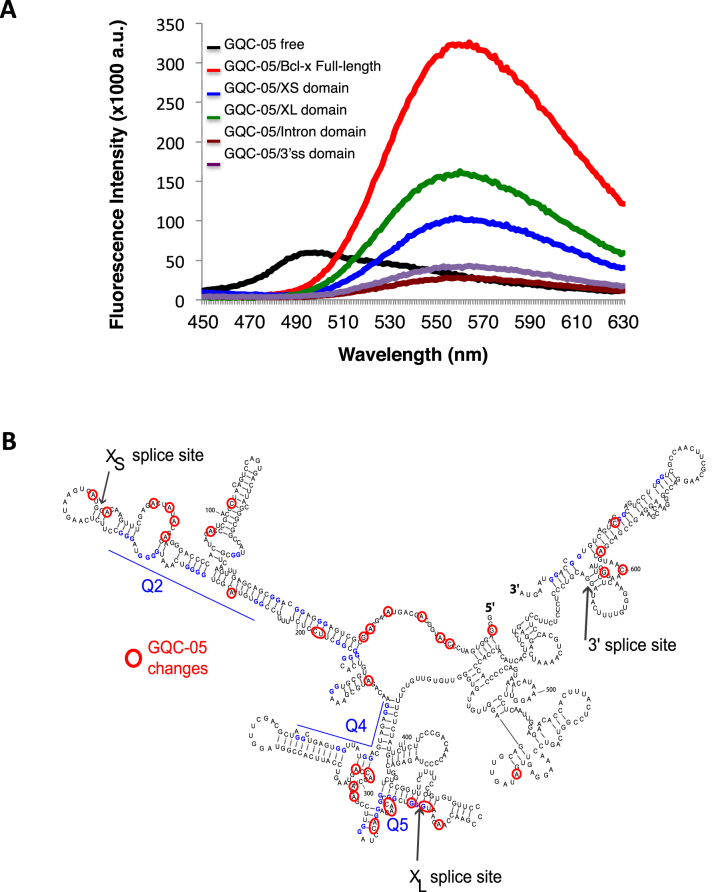
Direct interaction of GQC-05 with G4-forming sequences. (**A**) Fluorescence emission spectra of free GQC-05 (black), and GQC-05 in the presence of full-length Bcl-X-681 (FL) (red), X_S_ (blue), X_L_ (green), intron (brown) and 3′SS domains (purple). RNA was incubated at 1 μM with GQC-05 at 20 μM and excitation was at 321 nm. (**B**) Mapping of changes in RNase accessibility resulting from incubation with GQC-05 at 1, 5 and 10 μM. The nucleotides marked showed statistically significant differences in the value of the cleavage index (see ‘Materials and Methods’ section and Supplementary Data).

The ability of GQC-05 to alter usage of both splice sites might involve direct interactions with the Q2 and Q5 G4-forming sequences, but it was also possible that the effects were indirect consequences of interactions with splicing factors. To test whether GQC-05 binds directly to Bcl-X-681 pre-mRNA, its fluorescence was measured in the presence of the RNA. The intensity of fluorescence of GQC-05 upon excitation at 321 nm was increased 15-fold by the addition of Bcl-X-681 RNA, and the emission peak shifted from 493 to 558 nm (Figure 5A). This is consistent with direct binding. Furthermore, only the X_S_ and X_L_ domains, which contain Q2 and Q5, respectively, were bound by GQC-05, whilst the intron and 3′SS domains produced relatively little change. This is consistent with interactions with the G4-forming sequences.

To map the sites of interaction, RNA footprinting assays of Bcl-X-681 RNA were done in the presence of increasing concentrations of GQC-05 (Figure 5B, Supplementary Data). The intensities of the primer extension products following nuclease cleavage were expressed for each nucleotide as a ratio or index, [V1(10 μM GQC-05)/V1(no GQC-05)]/[T2(10 μM GQC-05)/T2(no GQC-05)]. The frequencies of the logarithm of this index formed a Gaussian distribution with a mean value of -0.0011 and a standard deviation of 0.20 (Supplementary Data). Positions at which the log (Index) differed by more than two standard deviations from the mean are shown in Figure 5B. The highest densities of changes in the secondary structure due to GQC-05 occurred in the same regions, Q2 and Q5, in which the secondary structures were most affected by deazaguanosine substitution (29). All of the changes in the Q2 region produced positive scores, indicating an increase in the proportion of molecules containing the structure, whereas in the Q5 region there were positive and negative scores, indicative of significant re-structuring. Another region with a high density of changes was the 5′ end, but the significance of the changes is unclear. We conclude that GQC-05 interacted directly with the Q2 and Q5 G4-forming regions in the Bcl-X-681 RNA.

### Interaction of GQC-05 with each G4-forming sequence affects the adjacent 5′SS

**Figure 6.**
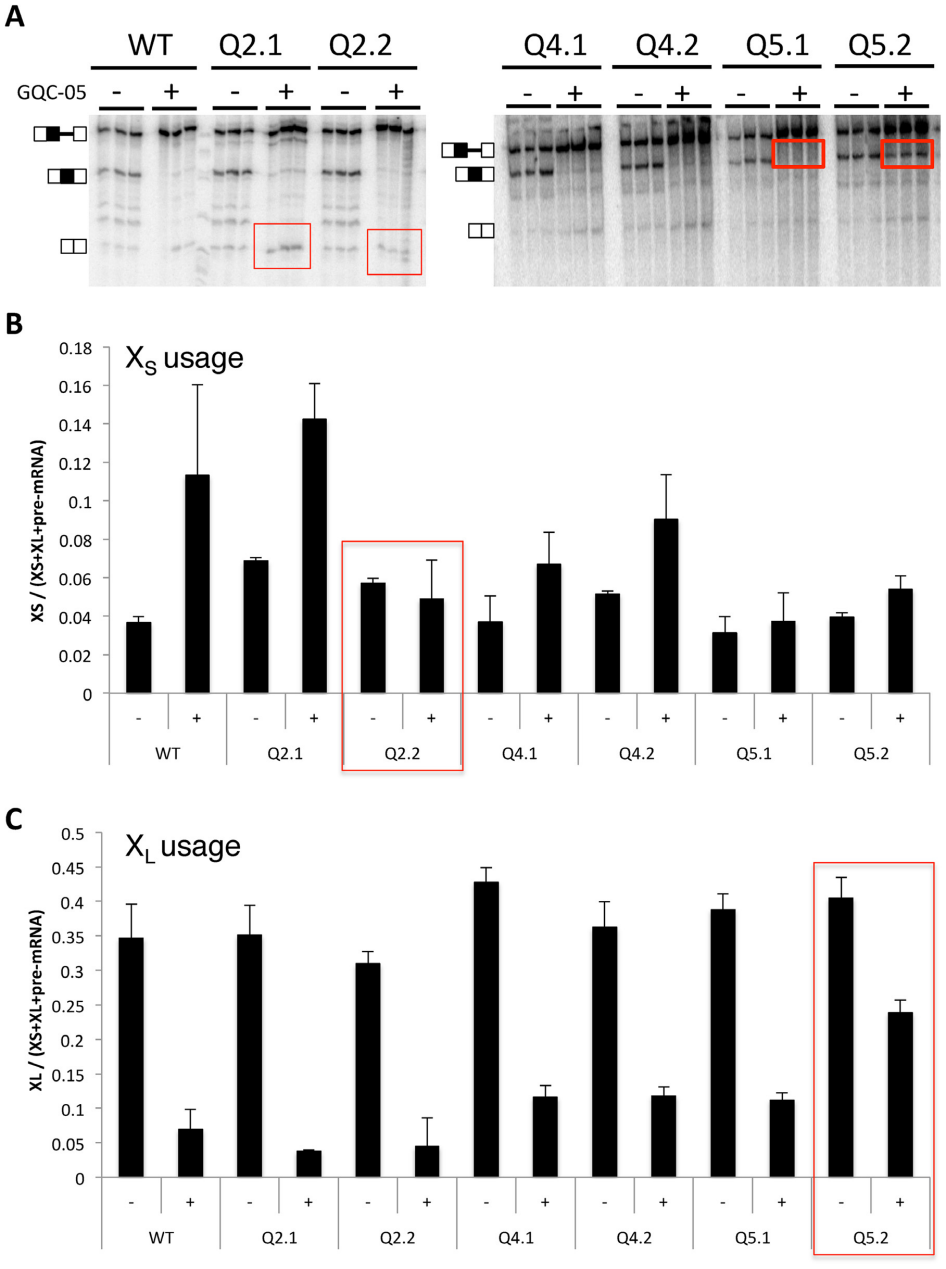
Effects on responses to GQC-05 of mutations that disrupt secondary structures or G4s. (**A**) *In vitro* splicing assays with mutations in the regions of Q2, Q4 and Q5. In Q2.1, 4.1 and 5.1, the mutations were intended to disrupt secondary structure but not the putative G4 (Supplementary Figure S5); in Q2.2, 4.2 and 5.2, the potential secondary structure was restored but the G4-forming potential was reduced. Assays were done in triplicate with GQC-05 present where shown at 40 μM. (**B**) Relative change in each mutant in the use of the X_S_ 5′SS (the bands were quantified and corrected for the radionucleotide content). (**C**) Corresponding changes in the use of the X_L_ 5′SS.

The data above show that various G4 ligands affect the splice sites independently, and that GQC-05 has opposite effects on the use of the two sites. Moreover, it binds G4-containing portions of the RNA and affects the structure of the pre-mRNA in the G4-forming regions Q2 and Q5. Q2 and Q5 are close to the X_S_ and X_L_ sites, respectively (Figures 1 and 5B), and it seemed likely that each G4 would only affect the use of the adjacent site. To test this, the pre-mRNA was mutated at two positions in each of Q2, Q5 and another region, Q4, which does not form a G4 (29). In each case (Q2.1, Q4.1 and Q5.1, as appropriate) CC was changed to AA (Supplementary Figure S7). The strategy (Supplementary Table S4A) was to control for the interconnections between secondary structure and G4 formation by mutating nucleotides that would affect only the secondary structure without significantly affecting the G4-forming potential. Compensating mutations were then made in each case to restore base-pairing but disrupt G4 formation (GG was changed to UU in Q2.2, Q4.2 and Q5.2). To confirm that these mutations affected GQC-05 interactions, the fluorescence emission spectra of the Q2.2 X_S_ and Q5.2 X_L_ domains were compared with the wild-type sequences. The emission peaks that we attributed to interaction with G4s were greatly reduced (Supplementary Figure S8). Splicing assays were then done in the presence or absence of GQC-05 (Figure 6).

Although changing the secondary structure potential in the Qx.1 mutants might affect the equilibrium between secondary structures, protein binding and G4 formation, G4 formation should still be possible in Q2.1 and Q5.1 and they should still respond to G600QC-05. However, the relatively strong stimulation of X_S_ splicing by GQC-05 seen in WT, Q2.1 and Q4.1 was almost eliminated in Q5.1 (Figure 6 and Supplementary Table S4B), notwithstanding the fact that the mutations made were proximal to the X_L_ site. Disrupting the ability to form G4s in mutants Q2.2 and Q5.2 should affect the GQC-05 response at whichever site the G4 regulated. Accordingly, disruption of the potential G4 in mutant Q2.2 prevented the up-regulation of the X_S_ 5′SS by GQC-05, even though X_L_ was downregulated as usual, and the disruption of the potential G4 in mutant Q5.2 substantially reduced the down-regulation of the X_L_ 5′SS by GQC-05. The small effect of GQC-05 on X_S_ 5′SS up-regulation in Q5.2 is consistent with the near-loss of the response in mutant Q5.1 (above). We conclude that the interaction of Q2 and Q5 with GQC-05 affects in each case primarily the adjacent 5′ splice site.

## DISCUSSION

We have shown here that a range of ellipticine and quindoline derivatives exhibit diverse effects on the patterns of 5′SS usage in Bcl-X, while other chemical classes of well-characterized G4 ligands have no effect. The diversity appears to be a result of specific substitutions of the ellipticine core. GQC-05 has opposite and apparently independent effects on the two alternative 5′SS, which appear to be the result of interactions in each case with the adjacent G4. It has a strikingly greater effect than the other derivatives, indicating that it forms quite specific interactions with the G4s.

Given the complexity of the interactions, it is not surprising that a number of potential ligands had no functional effects in our assay and that others affected just one site. Only GQC-05 and closely related molecules showed effects on both 5′ splice sites (Figures 2 and 3). The degree of specificity was even more striking in assays with HeLa cells (Figure 4 and Supplementary Table S3). The relatively stringent requirements for GQC-05 activity suggest that only a minority of RNA G4s will interact with GQC-05 *in vivo* and, moreover, suggest that further modifications to the side chains could be tailored to increase efficacy and specificity in future.

**Figure 7.**
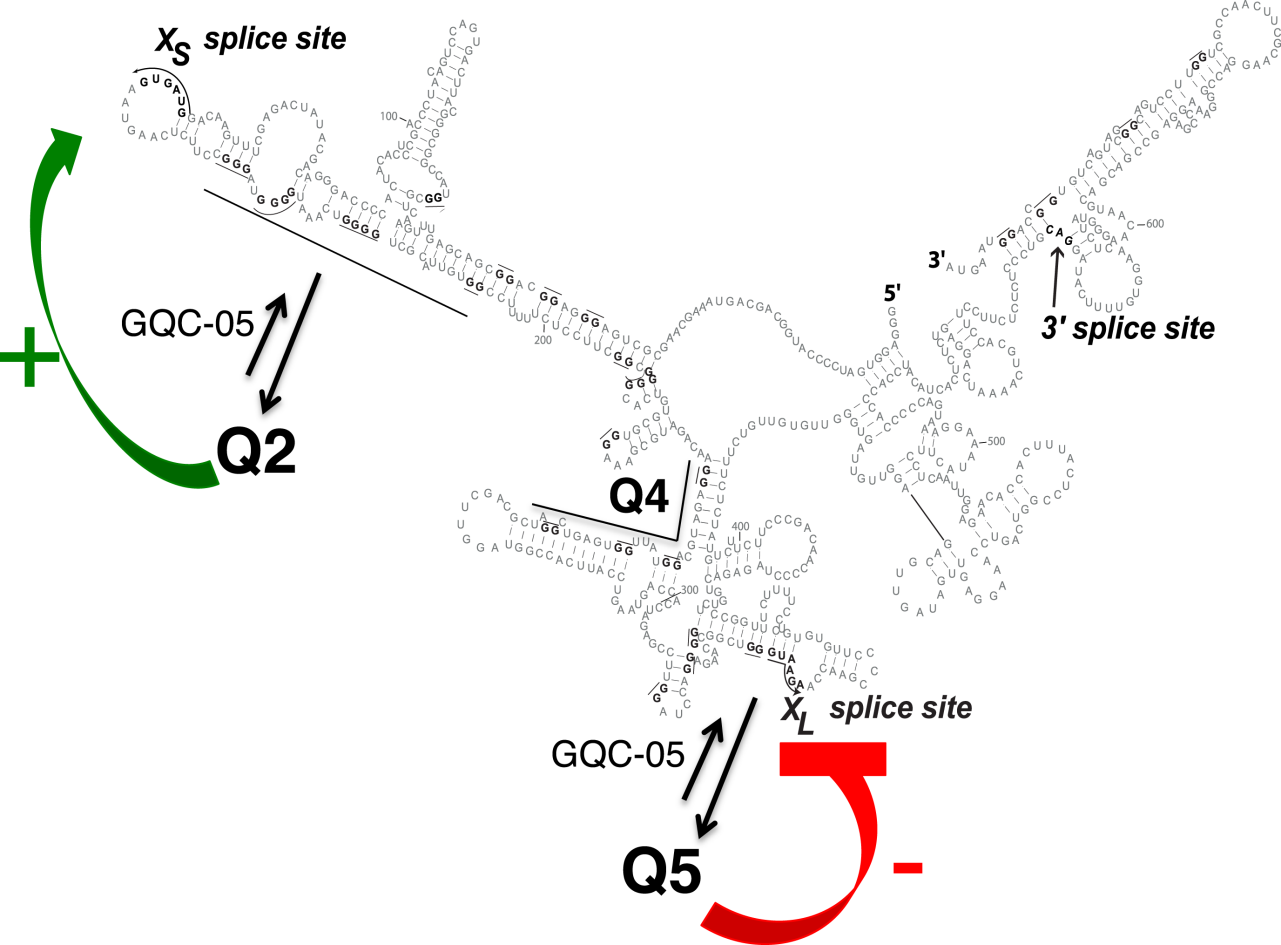
Model for the effects of G4s and GQC-05 on the Bcl-X-681 pre-mRNA. The secondary structure is shown as before, with arrows indicating that the presence of GQC-05 increases the proportion of pre-mRNA adopting the G4 conformations. Further studies will be required to establish whether there is a dynamic flux, forming an equilibrium distribution, or whether the initial condition adopted is stable for the lifetime of the pre-mRNA. The formation of a G4 downstream of the X_S_ 5′SS is proposed to destabilize a stem-loop that inhibits use of the X_S_ splice site, while the formation of a G4 overlapping the X_L_ 5′SS is proposed to directly inhibit use of the 5′SS, possibly by preventing U1 snRNP binding. The mutagenesis data support the proposal for the X_S_ site but the model is incomplete because it does not explain the effects of mutations near X_L_ on X_S_ splicing.

It is not clear to what extent the putative G4-stabilizing molecules are binding pre-formed G4s or inducing their formation. If the G4 itself affects splicing, then the assumption must be that the proportion of molecules in which G4s had formed is quite low in the absence of the stabilizer, as the stabilizer would otherwise have limited additional effects. The other, rarely-considered, possibility is that it is the ligand itself that affects splicing, and that pre-formed G4s recruit it. Our deazaguanine mapping experiments showed that Q2 and Q5 exist to at least some extent in isolated RNA and in nuclear extracts (29), and the fact that values of the index in the Q2 region were all positive in the presence of GQC-05 suggests that the proportion of G4-containing molecules increased in the presence of GQC-05. The mixture of positive and negative scores for Q5 is not inconsistent with the existence of complex secondary structures that are shifted toward the G4 by GQC-05. We conclude that GQC-05 is most likely to act on Bcl-X by increasing the proportion of the molecules forming G4s (Figure 7), but we note that in other cases where stabilizers have been used the structures may not form in the absence of the stabilizer.

There are no general models yet for the mechanisms by which G4s affect splicing. Q2 is to the 3’ side of the XS 5′SS, which is favored by G4 formation. While G4 formation would antagonize binding by hnRNP H, it is unlikely that binding here by hnRNP H would be detrimental. We note instead that the X_S_ site is at the tip of a long and relatively stable stem-loop. Such structures reduce usage of alternative 5′SS (46,47). It is likely that GQC-05 stabilizes a G4 that forms at the expense of the stem, thus increasing the availability and activity of the X_S_ site.

Q5 is just 5′ to the X_L_ 5′SS, which is inhibited by GQC-05 and, by inference, G4 formation. The inhibition might arise because the GG/G motif around the 5′SS (shown by /) is likely to be incorporated into any G4 that might form here. This is likely to impede binding by U1 snRNP. The fact that X_L_ is the dominant splice site under normal circumstances emphasizes that the natural level of G4 formation is low or that the G4 is transient and the U1 snRNP may enter and form an irreversible complex. This is consistent with the relatively small number of differences in Q5 between normal and deazaG-RNA (29) and the relatively large number of differences in Q5 with and without GQC-05 (Figure 5B).

A model assuming that the response to GQC-05 is the result of enhancing G4 formation at the expense of secondary structures makes predictions about the effects of the mutations (QX.1, QX.2) on basal as well as GQC-05-induced splicing patterns. These predictions are shown in Supplementary Table S4A. The effects of mutations in Q2 (Q2.1 and Q2.2) and their responsiveness to GQC-05 are fully in agreement with a model in which stem-loop formation is in competition with G4 formation and suppresses X_S_ 5′SS usage. In line with the reasoning above, which suggests that the X_L_ 5′SS is generally unaffected by either secondary structures or G4s, the effects of mutations in Q5 do not fit the predictions (Supplementary Table S4B). However, the most surprising result was that mutations in Q5.1 prevented the normal increase in usage of the distant X_S_ 5′SS in response to GQC-05. This might be the result of altered patterns in protein binding or of altered long-range secondary structures that are difficult to map. We conclude that the secondary structure-G4 competition model fits the X_S_ site but not the X_L_ site, and further work will be needed to understand this aspect of regulation by a G4. Given the success with Q2, however, it would be interesting to map secondary structures around G4s that have been proposed to affect splicing in other genes to establish whether this might be a general mechanism of action of G4 stabilizers.

Finally, we note that GQC-05 was originally selected as a compound that bound to a DNA G4-forming sequence in the MYC promoter and induced apoptosis in a Burkitt's lymphoma (BL) cell line (39). Another BL cell line in which translocation had removed the G4-forming sequence was less sensitive to GQC-05, from which it was inferred that the MYC promoter was the most sensitive target (39). The IC50 for the second cell line was still around 13 μM, raising the question as to the mechanisms of toxicity. This question is especially interesting given that other G4-containing promoters were insensitive to GQC-05 (39). It is possible that the effects of GQC-05 on the patterns of splicing of Bcl-X and, possibly, other genes might contribute toward this effect. It might be possible to tailor the specificity of G4 ligands that modulate RNA splicing still further to develop new compounds with therapeutic potential.

## Supplementary Material

Supplementary Data
