# Supplementary material for: Specific G-quadruplex ligands modulate the alternative splicing of Bcl-X
Source: Nucleic Acids Res. 2017 Nov 16;46(2):886–96. doi: 10.1093/nar/gkx1122 (PMC5778605; doi:10.1093/nar/gkx1122)
Supplement: Supplementary Data [file gkx1122_supp.zip › nar-01717-f-2017-File003.pdf]

Supplementary Material

Specific G-quadruplex ligands modulate the alternative splicing of Bcl-X

Carika Weldon[1], Justine G. Dacanay[1], Vijay Gokhale[2], P. Venkat L. Boddupally[3], Isabelle Behm-Ansmant[4], Glenn A. Burley[5], Christiane Branlant[4], Laurence H. Hurley[2,6], Cyril Dominguez[1*], Ian C. Eperon[1*]

[1] Leicester Institute of Structural & Chemical Biology and Department of Molecular & Cell Biology, University of Leicester, Leicester, UK

[2] College of Pharmacy and College of Pharmacy and BIO5 Institute, University of Arizona, Tucson, Arizona 85721, United States

[3] Fluoroorganic Division, CSIR-Indian Institute of Chemical Technology, Hyderabad, India-500 007

[4] IMoPA (Ingénierie Moléculaire et Physiopathologie Articulaire), UMR 7365 CNRS-UL, Biopôle de l'Université de Lorraine, 9 Avenue de la Forêt de Haye, 54505 Vandoeuvre-lès-Nancy, France

[5] Department of Pure and Applied Chemistry, University of Strathclyde, UK

[6] Arizona Cancer Center, University of Arizona, Tucson, Arizona 85724, United States

SUPPLEMENTARY MATERIALS AND METHODS

Syntheses and characterization of ellipticines

GSA-1113

To a solution of 9-methoxy-5,11-dimethyl-6*H*-pyrido[4,3-*b*]carbazole (500 mg, 1.81 mmol) in DMF (20 mL) was added sodium hydride (173 mg, 7.24 mmol) at 0 ^{0}C over a period of 10 min. then the reaction mixture was stirred at the same temperature for 1 h. To this reaction mixture, dimethylaminoethylbromide hydrobromide (632 mg, 2.71 mmol) was added and the stirring was continued for overnight at room temperature. After completion of the reaction, as indicated by TLC (10 % MeOH in DCM) the reaction mixture was poured on to cold water and neutralized with ammonium chloride solution. Aqueous layer was extracted with ethyl acetate (2x 100 mL), the combined organic layer was washed with water (2 x 200 mL), dried over anhydrous sodium sulfate and finally distilled under vacuum to get crude product. Then the crude product was purified on Biotage column purification system using 2-10 % MeOH in DCM as a gradient solvent system to yield 60 mg of GSA1113.

^{1}H-NMR ($CDCl_3$, 300 MHz): δ 9.70 (s, 1H, ArH), 8.51 (d, *J* = 6.0 Hz, 1H, ArH), 8.00-7.87 (m, 2H, ArH), 7.38 (d, *J* = 8.7 Hz, 1H, ArH), 7.22 (dd, *J* = 2.1 & 8.7 Hz, 1H, ArH), 4.63 (t, *J* = 7.8 Hz, 2H), 3.98 (s, 3H), 3.24 (s, 3H), 3.01 (s, 3H), 2.75 (t, *J* = 7.9 Hz, 2H), 2.40 (s, 6H).

^{13}C-NMR ($CDCl_3$): 154.41, 150.18, 142.03, 141.46, 139.76, 134.80, 129.49, 125.28, 124.84, 116.30, 114.88, 109.44, 108.72, 58.64, 56.69, 46.44, 45.13, 15.10, 13.93.

HPLC Purity: Zorbax SB C18, 4.6 x150 mm, 3.5 u, Mobile phase: 30/70/0.25, MeOH: water: HCOOH, Flow rate: 0.5 mL/min. 100 % (R.T. : 6.752 min)

HRMS: Found = 348.2065 (MH^+) (Theoretically = 348.2070) Error = 1.4 ppm

GSA-1126

5,11-Dimethyl-6H-pyrido[4,3-b]carbazol-9-ol (558 mg, 2.12 mmol) was dissolved in *tert.* butanol and ethanol (15 mL + 15 mL) solvent and potassium. *tert.* butoxide (715 mg, 6.38 mmol) was added to that mixture. After 1 hr, *N,N*-diethylamino ethyl bromide hydrobromide (720 mg, 2.76 mmol) was added and the reaction mixture was refluxed at 80^{0}C for overnight. After completion of the reaction, the mixture was poured on to ice cold water and neutralized with ammonium chloride. The aqueous layer was extracted with ethyl acetate. The ethyl acetate layers were combined, dried with anhydrous sodium sulfate and concentrated. This crude material was purified by Biotage column chromatography using 15% methanol in $CHCl_3$ as an eluent to afford the pure product (80 mg, 10.41 %) as yellow solid.

^{1}H-NMR (DMSO, 300 MHz): δ 11.20 (s, 1H, NH), 9.67(s, 1H, ArH), 8.40 (d, J = 5.7 Hz, 1H, ArH), 7.93-7.83 (m, 2H, ArH), 7.52-7.43 (m, 1H, ArH), 7.22-7.14 (m, 1H, ArH), 4.16 (t, J = 4.8 Hz, 2H), 3.24 (s, 3H), 2.84 (t, J = 4.8 Hz, 2H), 2.75 (s, 3H), 2.68-2.54 (m, 4H), 1.08-0.94 (m, 6H).
^{13}C-NMR (DMSO, 75 MHz): 153.17, 150.60, 142.05, 141.26, 138.20, 133.17, 129.04, 124.40, 124.25, 122.53, 116.73, 116.62, 111.98, 109.57, 108.72, 68.15, 52.49, 47.91, 15.13 12.74.
HPLC Purity: Column: Zorbax SB C18, Mobile phase: 35/65/0.25. MeOH: water: formic acid, Retention time: 7.42. Purity: 98.11 %
HRMS: Found 362.2225 (Theoretically = 362.2232) Error = 0.6 ppm

GSA-1133

5,11-Dimethyl-6H-pyrido[4,3-b]carbazol-9-ol (500 mg, 1.90 mmol) was dissolved in *tert.* butanol and ethanol (15 mL + 15 mL) solvent and potassium. *tert.* butoxide (641 mg, 5.72 mmol) was added to that mixture. After 1 hr, 1-(2-bromoethyl) pyrrolidine hydrobromide (642 mg, 2.48 mmol) was added and the reaction mixture was reacted at room temperature for 4 h. After completion of the reaction, the mixture was poured on to ice cold water and neutralized with ammonium chloride. The aq. layer was extracted with ethyl acetate. The ethyl acetate layers were combined, dried with anhydrous sodium sulfate and concentrated. This crude material was purified by Biotage column chromatography using 8% methanol in $CHCl_3$ as an eluent to afford the pure product (60 mg, 8.7 %) as yellow solid.

^{1}H-NMR (DMSO, 300 MHz): δ 11.22 (s, 1H, NH), 9.68 (s, 1H, ArH), 8.40 (d, J = 6.0 Hz, 1H, ArH), 7.94-7.84 (m, 2H, ArH), 7.47 (d, J = 8.7 Hz, 1H, ArH), 7.19 (dd, J = 8.7 Hz, 1H, ArH), 4.21 (t, J = 6.0 Hz, 2H), 3.25 (s, 3H), 2.88 (t, J = 6.0 Hz, 2H), 2.76 (s, 3H), 2.65-2.54 (m, 4H), 1.65-1.77 (m, 4H).
^{13}C-NMR (DMSO, 75 MHz): 153.14, 150.62, 142.07, 141.27, 138.23, 133.19, 129.09, 124.43, 124.27, 122.55, 116.66, 111.99, 109.61, 108.74, 68.42, 55.49, 54.94, 24.03, 15.15, 12.77.
HPLC Purity: Zorbax SB C18, Mobile phase: 35/65/0.25. MeOH: water: formic acid, Retention time: 6.32. Purity: 100 %
HRMS: Found 360.2070(Theoretically = 360.2076) Error = 0.3 ppm

GSA-1135

5, 11-Dimethyl-6H-pyrido [4, 3-b] carbazol-9-ol (500 mg, 1.90 mmol) was dissolved in *tert.* butanol and ethanol (15 mL + 15 mL) solvent and potassium. *tert.* butoxide (641 mg, 5.72 mmol) was added to that mixture. After 1 hr 1-(2-chloroethyl) piperidine hydrochloride (456 mg, 2.48 mmol) was added and the

reaction mixture was refluxed at 80^{0}C for overnight. After completion of the reaction, the mixture was poured on to ice cold water and neutralized with ammonium chloride. The aq. layer was extracted with ethyl acetate. The ethyl acetate layers were combined, dried with anhydrous sodium sulfate and concentrated. This crude material was purified by Biotage column chromatography using 8% methanol in $CHCl_3$ as an eluent to afford the pure product (50 mg, 7.0 %) as yellow solid.

^{1}H-NMR (DMSO, 300 MHz): δ 11.21 (s, 1H, NH), 9.68 (s, 1H, ArH), 8.40 (d, J = 5.4 Hz, 1H, ArH), 7.94-7.84 (m, 2H, ArH), 7.46 (d, J = 8.7 Hz, 1H, ArH), 7.19 (d, J = 8.7 Hz, 1H, ArH), 4.21 (t, J = 5.4 Hz, 2H), 3.25 (s, 3H), 2.85-2.70 (m, 5H), 2.57-2.44 (m, 4H), 1.60-1.48 (m, 4H), 1.47-1.35 (m, 2H).

^{13}C-NMR (DMSO, 75 MHz): 153.14, 150.61, 142.06, 141.27, 138.23, 133.19, 129.08, 124.41, 124.27, 122.54, 116.76, 116.64, 111.98, 109.64, 108.73, 67.30, 58.51, 55.35, 26.40, 24.76, 15.16, 12.77.

HPLC Purity: Zorbax SB C18, Mobile phase: 40/60/0.25. MeOH: water: formic acid, Retention time: 5.60. Purity: 98.45 %

HRMS: Found 374.2226 (Theoretically = 374.2232) Error = -1.5 ppm

SUPPLEMENTARY TABLES

Structure class	Name	[G4L] (µM)	$X_S/(X_S+X_L)$	*P* value	Relative level of X_S	Relative level of X_L
Porphyrin	TMPyP4	0	0.15 ±0.02		-	-
		5	0.33 ±0.02	0.0003	0.63	0.24
		10	0.28 ±0.05	0.02	0.49	0.23
		25	N/A		0	0
Fluoro-quinolone	Quarfloxin	0	0.15 ±0.02		-	-
		1	0.12 ±0.01	0.03	1	1
		10	0.12 ±0.004	0.03	1	1
		40	0.14 ±0.01	0.2	1	1
Phthalocyanine	Zn-DIGP	0	0.34 ±0.06		-	-
		1	0.34 ±0.07	0.99	0.75	0.77
		5	0	0.0008	0	0.26
		10	N/A		0	0
Benzothiazole	Thioflavin T (ThT)	0	0.14 ±0.01		-	-
		1	0.14 ±0.06	0.97	1	1
		10	0.17 ±0.01	0.06	1	1
		50	0.22±0.02	0.008	0.82	0.50
Quinolinyl-pyridine-dicarboxamide	Pyridostatin (PDS)	0	0.36 ±0.05		-	-
		1	0.30 ±0.01	0.1	0.61	0.80
		5	0	0.0002	0	0.24
		10	N/A		0	0
Quinolinyl-pyridine-dicarboxamide	Carboxy-pyridostatin (cPDS)	0	0.36 ±0.5		-	-
		1	0.32 ±0.01	0.3	1	1
		5	0.33 ±0.11	0.7	1	1
		10	0.21 ±0.08	0.05	0.30	0.62
Naphthyridine	3AQN	0	0.17 ±0.03		-	-
		1	0.19 ±0.03	0.5	1	1
		5	0.17 ±0.04	0.8	0.46	0.44
		10	N/A	0.4	0	0
Naphthyridine	6AQN	0	0.17 ±0.03		-	-
		1	0.15 ±0.01	0.5	1	1
		5	0.22 ±0.03	0.1	1	0.62
		10	0.20 ±0.03	0.2	0.52	0.40
Bisquinolinium pyridines	360A	0	0.17 ±0.03		-	-
		1	0.14 ±0.02	0.3	1	1
		5	0.17 ±0.02	0.9	1	1
		10	0.19 ±0.01	0.3	0.65	0.56
Ellipticine	GQC-05	0	0.15 ±0.01		-	-
		1	0.10 ±0.01	0.004	1	1
		10	0.28 ±0.07	0.04	1.82	1
		40	0.80 ±0.02	0.000001	2.86	0.13
Quindoline	SYUIQ-5	0	0.23 ±0.03		-	-
		40	0.41 ± 0.05	0.006	1.82	1
11-piperazinyl-quindolines	GSA-0902	0	0.17 ±0.04		-	-
		1	0.11 ±0.02	0.08	0.20	1
		10	0.06 ±0.004	0.01	0.34	1
		40	0.04 ±0.01	0.006	0.18	1

Supplementary Table 1. Effects of a variety of G4 ligands on the splicing of Bcl-X-681 *in vitro*. The chemical class of the ligand, its specific name and its concentrations in the splicing reaction are shown. The yields of splicing were calculated as in Materials and Methods. $X_S/(X_S+X_L)$ shows the proportion of the X_S isoform in the mRNA. The significance of the difference in the mean value from the mean of the control reaction done in the same experiment (boxed) was tested using Student's *t* test (*P*). The relative level is the yield of each isoform compared to the control.

Name	[G4L] µM	$X_S/(X_S+X_L)$	*P* value	Relative level of X_S	Relative level of X_L
-	0	0.23 ± 0.03		-	-
quindoline	40	0.30 ± 0.01	0.02	1.59	1
SYUIQ-5	40	0.41 ± 0.05	0.006	1.82	1
-	0	0.21 ± 0.01		-	-
	1	0.16 ± 0.04	0.09	0.72	1
GSA-0819	10	0.14 ± 0.01	0.0006	0.69	1
	40	0.22 ± 0.02	0.5	1	1
	1	0.18 ± 0.06	0.4	1	1
GSA-0820	10	0.08 ± 0.01	0.0001	0.33	1
	40	0.06 ± 0.01	0.0001	0.22	1
	1	0.14 ± 0.01	0.003	1	1
GSA-0825	10	0.18 ± 0.04	0.2	1	1
	40	0.15 ± 0.01	0.004	1	1
-	0	0.17 ± 0.04		-	-
	1	0.11 ± 0.02	0.08	0.67	1
GSA-0902	10	0.06 ± 0.004	0.01	0.34	1
	40	0.04 ± 0.01	0.007	0.18	1
	1	0.12 ± 0.01	0.1	0.83	1
GSA-0903	10	0.13 ± 0.01	0.1	0.83	1
	40	0.13 ± 0.01	0.2	0.83	1
	1	0.12 ± 0.01	0.1	0.78	1
GSA-0924	10	0.12 ± 0.01	0.1	0.75	1
	40	0.11 ± 0.01	0.08	0.63	1
-	0	0.19 ± 0.01		-	-
	1	0.14 ± 0.005	0.001	0.73	1
GSA-0925	10	0.11 ± 0.02	0.003	0.52	1
	40	0.09 ± 0.03	0.004	0.45	1
	1	0.12 ± 0.01	0.0006	0.56	1
GSA-0927	10	0.12 ± 0.01	0.001	0.61	1
	40	0.10 ± 0.03	0.006	0.53	1
	1	0.13 ± 0.02	0.005	0.65	1
GSA-0932	10	0.11 ± 0.03	0.006	0.54	1
	40	0.10 ± 0.03	0.005	0.48	1
-	0	0.15 ± 0.01		-	-
	1	0.14 ± 0.003	0.05	0.88	1
GSA-0933	10	0.10 ± 0.03	0.03	0.65	1
	40	0.15 ± 0.01	0.5	1	1
	1	0.12 ± 0.01	0.02	0.75	1
GSA-1013	10	0.12 ± 0.01	0.009	0.81	1
	40	0.14 ± 0.01	0.1	1	1
	1	0.14 ± 0.02	0.3	0.85	1
GSA-1015	10	0.13 ± 0.01	0.07	0.81	1
	40	0.13 ± 0.01	0.06	0.76	1

Supplementary Table 2. Effects of quindolines on the splicing of Bcl-X-681 *in vitro*. The values shown are as described in the legend to Supplementary Table 1.

Compound	$X_S/(X_S+X_L)$ (DMSO control)	Standard deviation	$X_S/(X_S+X_L)$ (Treated)	Standard deviation (Treated)	*P* value
GQC-05	0.173	0.008	0.543	0.027	<0.0001*
GSA-1112	0.169	0.018	0.226	0.056	0.236
GSA-1113	0.174	0.017	0.264	0.024	0.011
GSA-1125	0.161	0.016	0.392	0.088	0.022
GSA-1126	0.172	0.023	0.255	0.005	0.007*
GSA-1133	0.098	0.028	0.255	0.032	0.006*
GSA-1135	0.112	0.024	0.195	0.045	0.081
Quindoline	0.092	0.007	0.138	0.031	0.107
GSA-0819	0.111	0.015	0.125	0.011	0.355
GSA-0820	0.096	0.003	0.173	0.015	0.002*
GSA-0825	0.106	0.018	0.188	0.035	0.044
GSA-0902	0.062	0.020	0.062	0.005	0.997
GSA-0903	0.128	0.007	0.194	0.014	0.004*
GSA-0924	0.115	0.009	0.110	0.006	0.519
GSA-0925	0.055	0.006	0.167	0.105	0.208
GSA-0927	0.069	0.009	0.115	0.021	0.048
GSA-0932	0.123	0.023	0.098	0.013	0.259
GSA-0933	0.119	0.020	0.174	0.006	0.018
GSA-1013	0.081	0.009	0.110	0.009	0.038
GSA-1015	0.080	0.011	0.127	0.005	0.005*
SYUIQ-5	0.089	0.009	0.080	0.009	0.350
Quarfloxin	0.111	0.016	0.122	0.007	0.418
ThT	0.200	0.030	0.218	0.062	0.733
Zn-DIGP	0.048	0.010	0.043	0.010	0.636
TmPyP4	0.084	0.012	0.065	0.011	0.179
PDS	0.103	0.014	0.122	0.025	0.405
cPDS	0.103	0.019	0.109	0.008	0.687
3AQN	0.039	0.009	0.036	0.014	0.800
6AQN	0.046	0.013	0.044	0.007	0.840
360A	0.231	0.030	0.176	0.032	0.147

Supplementary Table 3. Effects of G4 ligands on the splicing of Bcl-X in HeLa cells. The significance of the effects was tested using Student's *t* test (*P*). An asterisk indicates $P < 0.01$. Only GQC-05 and GSA-1133 show significant changes with a greater than two-fold increase in X_S.

A

	No GQC-05		+ GQC-05	
**Pre-mRNA**	**X_S**	**X_L**	**X_S**	**X_L**
	**Change relative to Bcl-X-681**		**Change caused by GQC-05**	
**Q2.1**	Increase	No change	Increase	Decrease
**Q4.1**	No change	No change	Increase	Decrease
**Q5.1**	No change	Decrease	Increase	Decrease
	**Change relative to QN.1**		**Change caused by GQC-05**	
**Q2.2**	Decrease	No change	No or less increase	Decrease
**Q4.2**	No change	No change	Increase	Decrease
**Q5.2**	No change	Increase	Increase	No or less decrease

B

	No GQC-05				+ GQC-05			
	**Splice site usage (%)**		***t* test**		**Splice site usage (%)**		***t* test vs no GQC-05**	
**Pre-mRNA**	**X_S**	**X_L**	***p*X_S**	***p*X_L**	**X_S**	**X_L**	***p*X_S**	***p*X_L**
**Bcl-X-681**	3.7	34.7			11.3	7.0	0.048	0.001
			***t* test vs Bcl-X-681**					
**Q2.1**	6.9	35.1	0.000	0.914	14.2	3.8	0.001	0.000
**Q4.1**	3.7	42.8	0.948	0.058	6.7	11.6	0.036	0.000
**Q5.1**	3.1	38.8	0.379	0.257	3.7	11.2	0.288	0.000
			***t* test vs QN.1**					
**Q2.2**	5.7	30.9	0.001	0.198	4.9	4.5	0.521	0.000
**Q4.2**	5.2	36.2	0.138	0.058	9.0	11.8	0.023	0.000
**Q5.2**	3.9	40.5	0.188	0.477	5.4	23.8	0.012	0.001

Supplementary Table 4. Predicted and measured effects on splicing of mutations in the potential quadruplex-forming regions of Bcl-X. (A) Predictions for the behaviour of the X_S and X_L splice sites were based on the model described in the Discussion, i.e., that secondary structure occludes the X_S 5'SS but Q2 G4 formation would make it more accessible, and that Q5 G4 formation would interfere with usage of the X_L 5'SS. Q4 is a control, a potential quadruplex that does not appear to form. Q2.1, Q4.1 and Q5.1 are mutations that would destabilize secondary structures without affecting the quadruplex, and their effects are judged relative to the WT sequence; Q2.2, 4.2 and 5.2 are second-site mutations that restore base-pairing but would disrupt the G4, and their effects are predicted in comparison to the initial mutations. The effects of GQC-05 in all cases are predicted relative to the same sequence in the absence of GQC-05. (B) Outcomes of splicing assays *in vitro* (Figure 6). In the absence of GQC-05, the t-tests examine the null hypotheses that the mutants Q2.1, Q4.1 and Q5.1 splice like the non-mutant Bcl-X-681 and that mutants Q2.2, Q4.2 and Q5.2 splice like Q2.1, Q4.1 and Q5.1, respectively. In the presence of GQC-05, the null hypothesis is that GQC-05 has no effect on splicing.

SUPPLEMENTARY DATA

Effects of GQC-05 at 3 concentrations (1, 5, and 10 µM) on Bcl-X footprinting. This Table is deposited as a separate file because of its size. It contains an analysis of the data contained in gels displayed in Supplementary Figure 4 of Weldon *et al.*, Nat. Chem. Biol. 13, 18-21 (2017), which contained previously unanalysed lanes in which GQC-05 had been added to normal and deazaguanine-containing RNA prior to footprinting using T1, T2 and V1 nucleases.

Weldon et al. Supplementary Figure S1

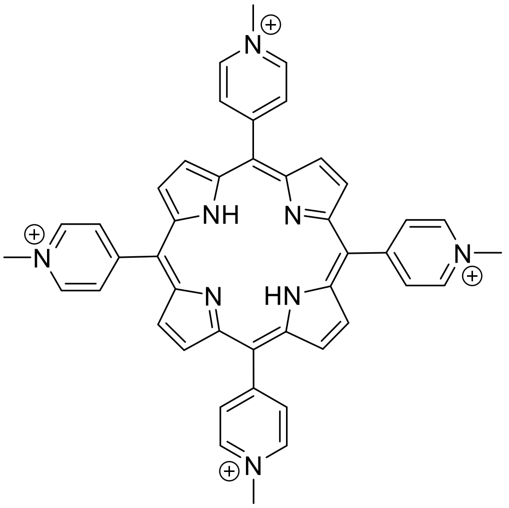

TMPyP4

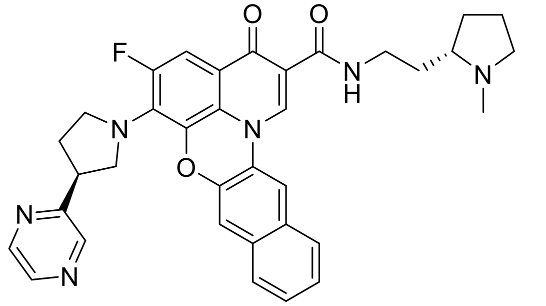

Quarfloxin

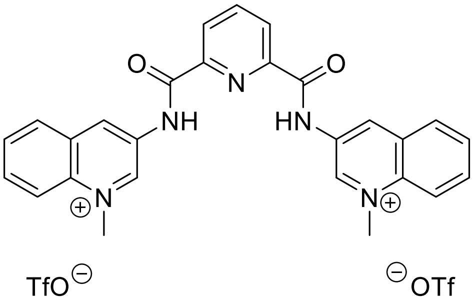

360A

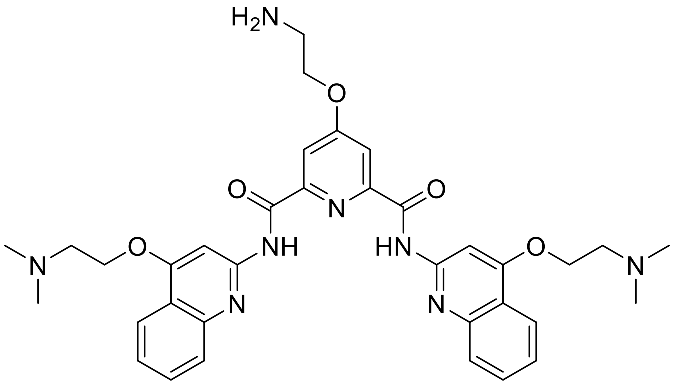

Pyridostatin (PDS)

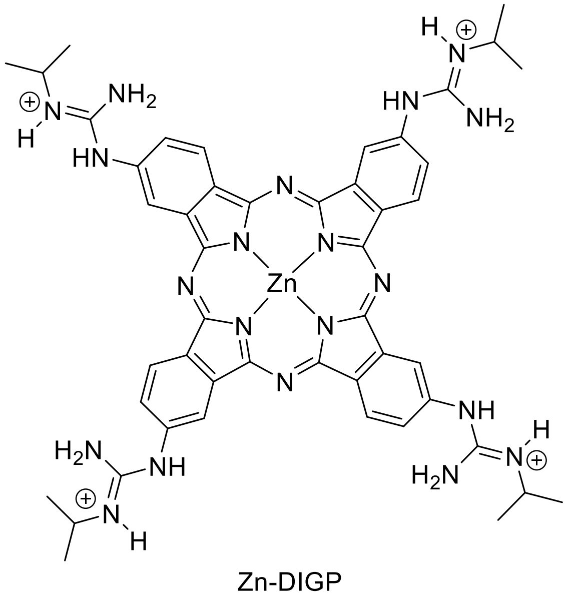

Zn-DIGP

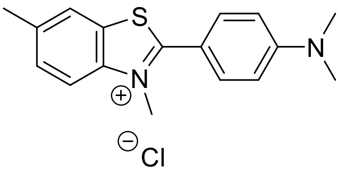

Thioflavin T

Ellipticine Series

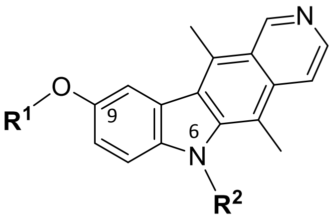

	R1	R2
GQC-05	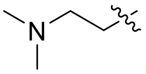	H
GSA-1112	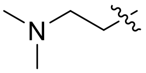	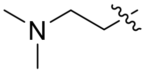
GSA-1113	Me	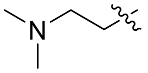
GSA-1125	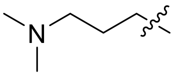	H
GSA-1126	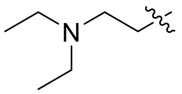	H
GSA-1133	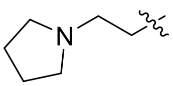	H
GSA-1135	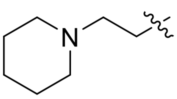	H

Quindoline Series

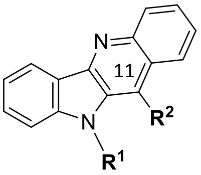

	**R1**	**R2**
Quindoline	H	H
SYUIQ-5	H	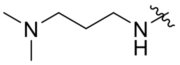
GSA-0819	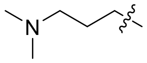	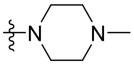
GSA-0820	H	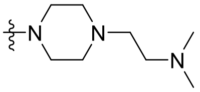
GSA-0902	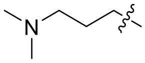	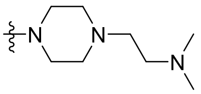
GSA-0924	H	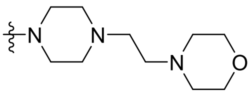
GSA-0933	H	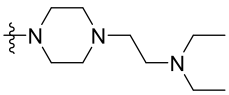
GSA-1013	H	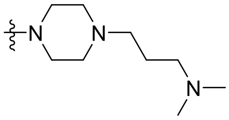
GSA-1015	H	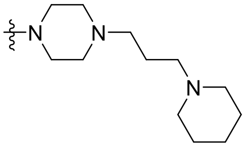

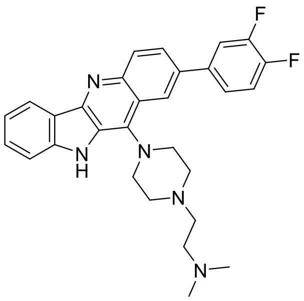

GSA-0925

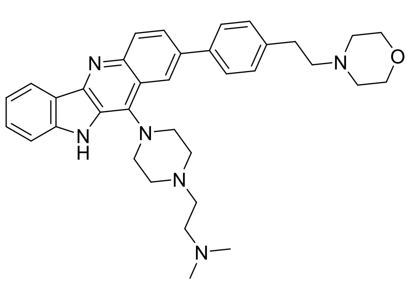

GSA-0927

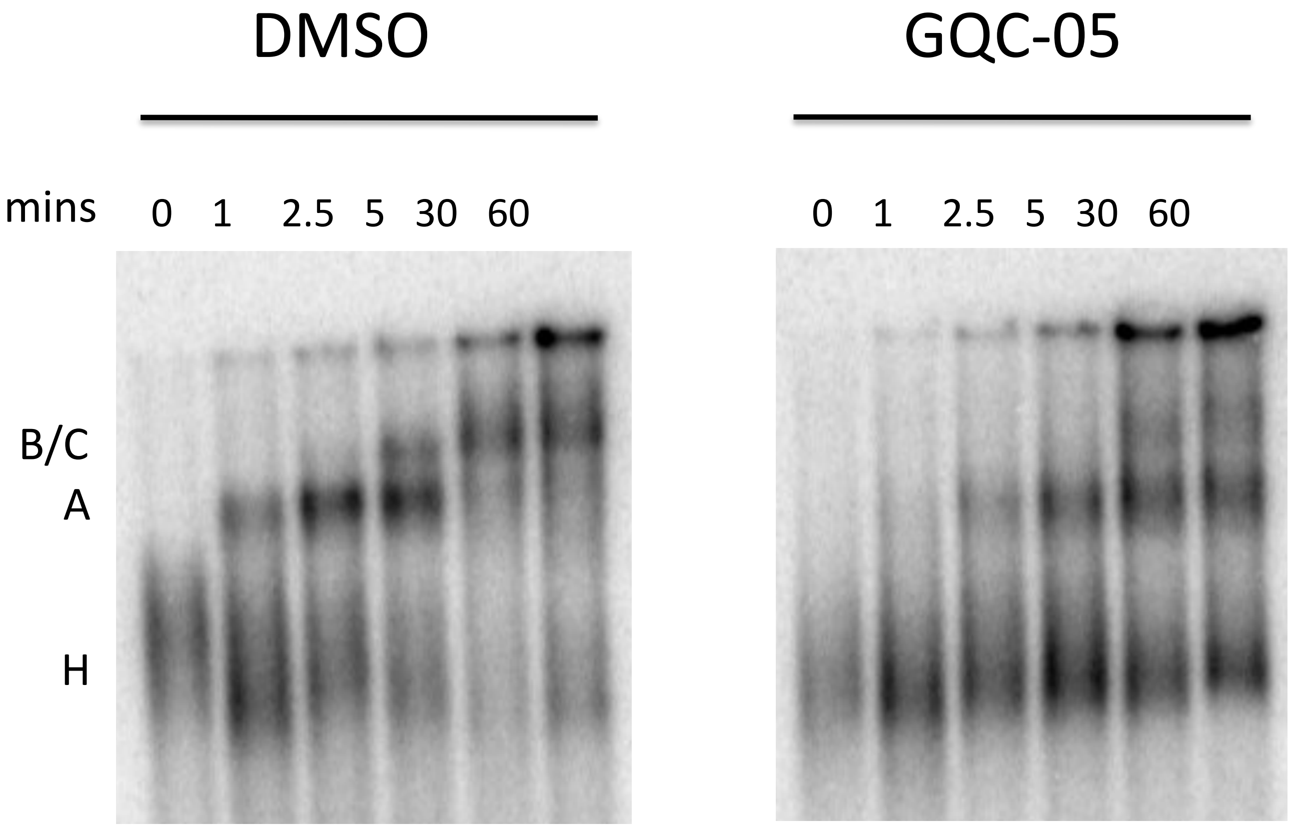
DMSO
GQC-05
mins
0 1 2.5 5 30 60
0 1 2.5 5 30 60
B/C
A
H

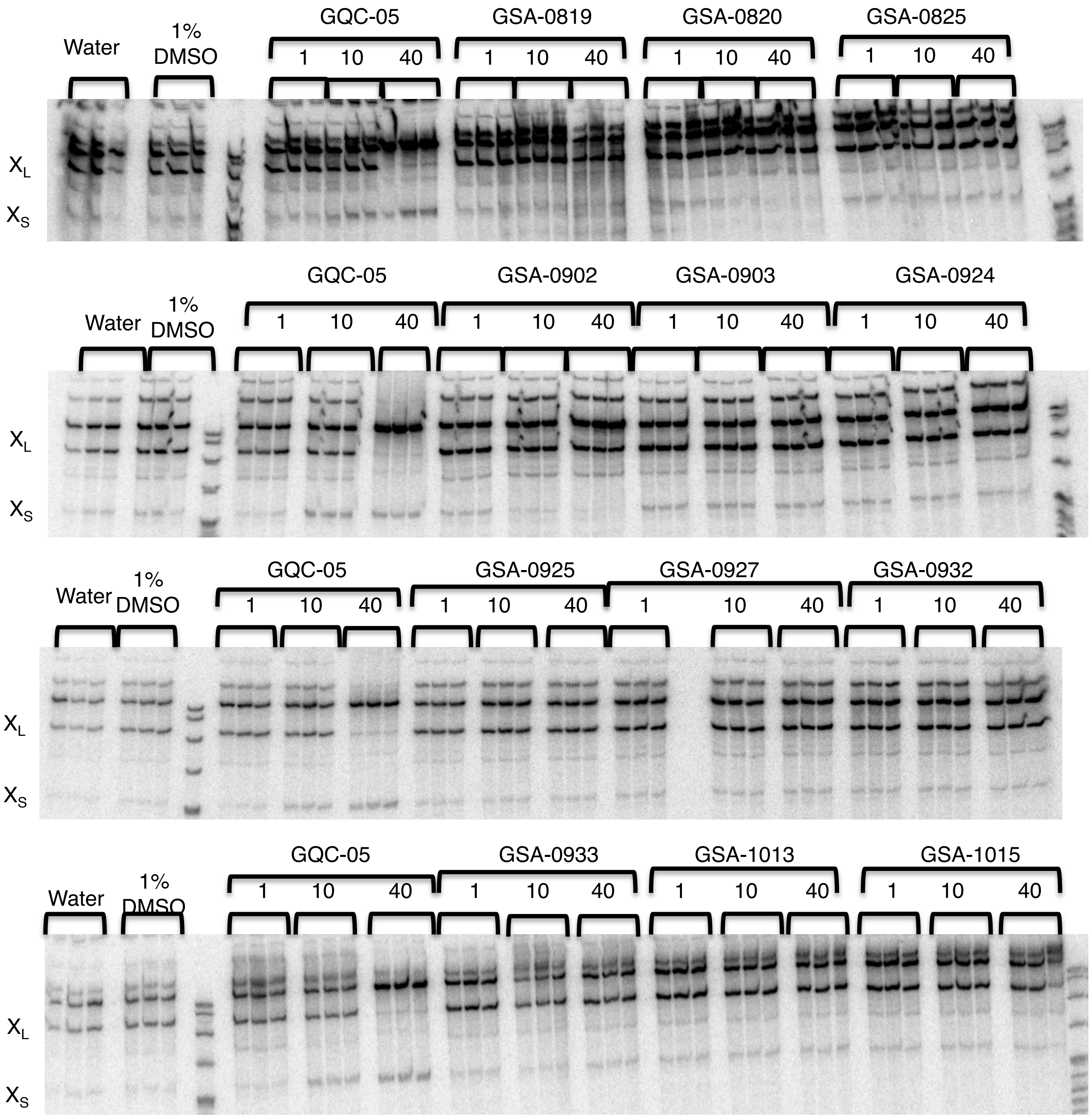

Water
1% DMSO
GQC-05
GSA-0819
GSA-0820
GSA-0825
1 10 40
X_L
X_S
Water
1% DMSO
GQC-05
GSA-0902
GSA-0903
GSA-0924
1 10 40
X_L
X_S
Water
1% DMSO
GQC-05
GSA-0925
GSA-0927
GSA-0932
1 10 40
X_L
X_S
Water
1% DMSO
GQC-05
GSA-0933
GSA-1013
GSA-1015
1 10 40
X_L
X_S

Weldon et al. Supplementary Figure S6

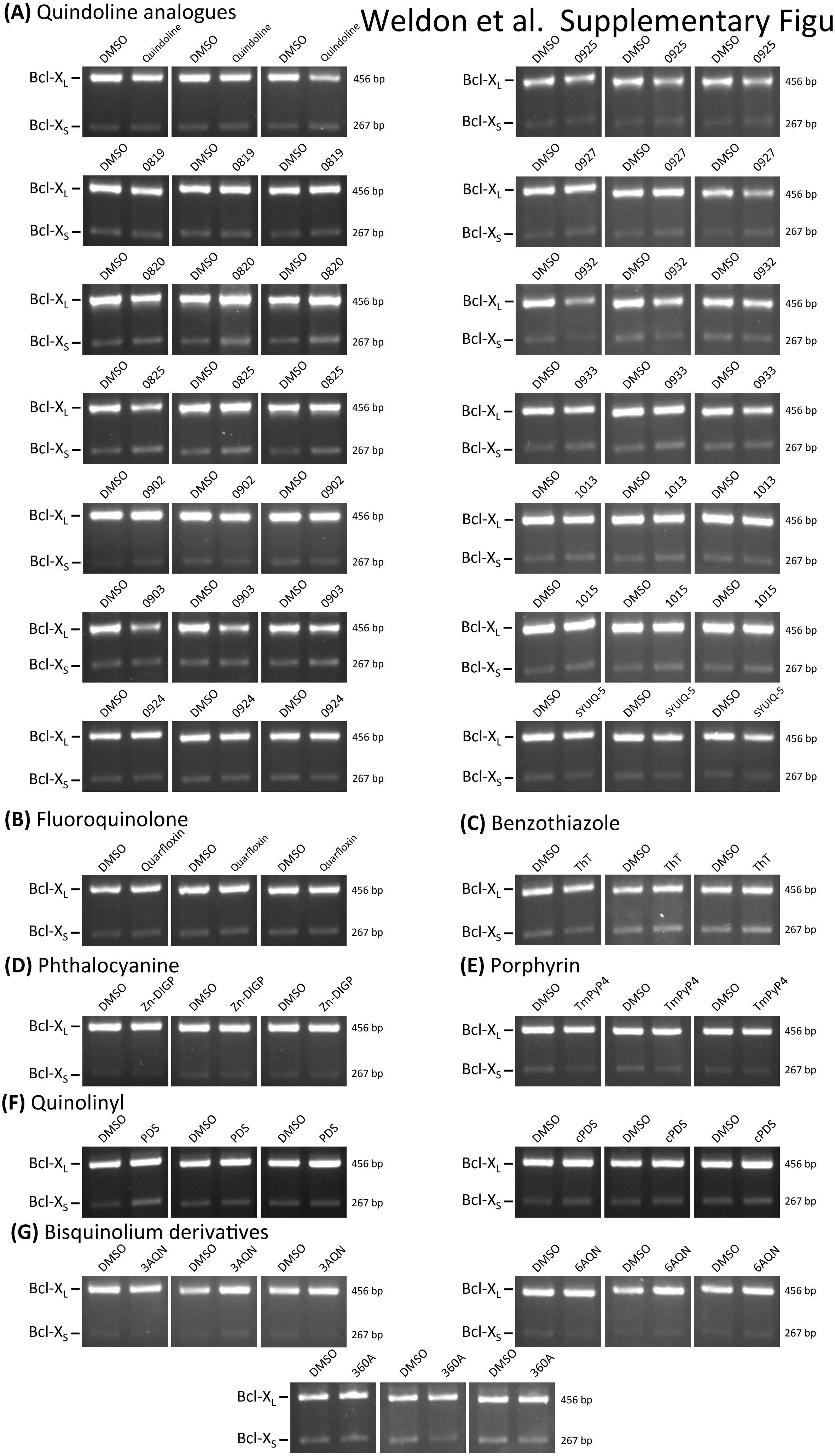

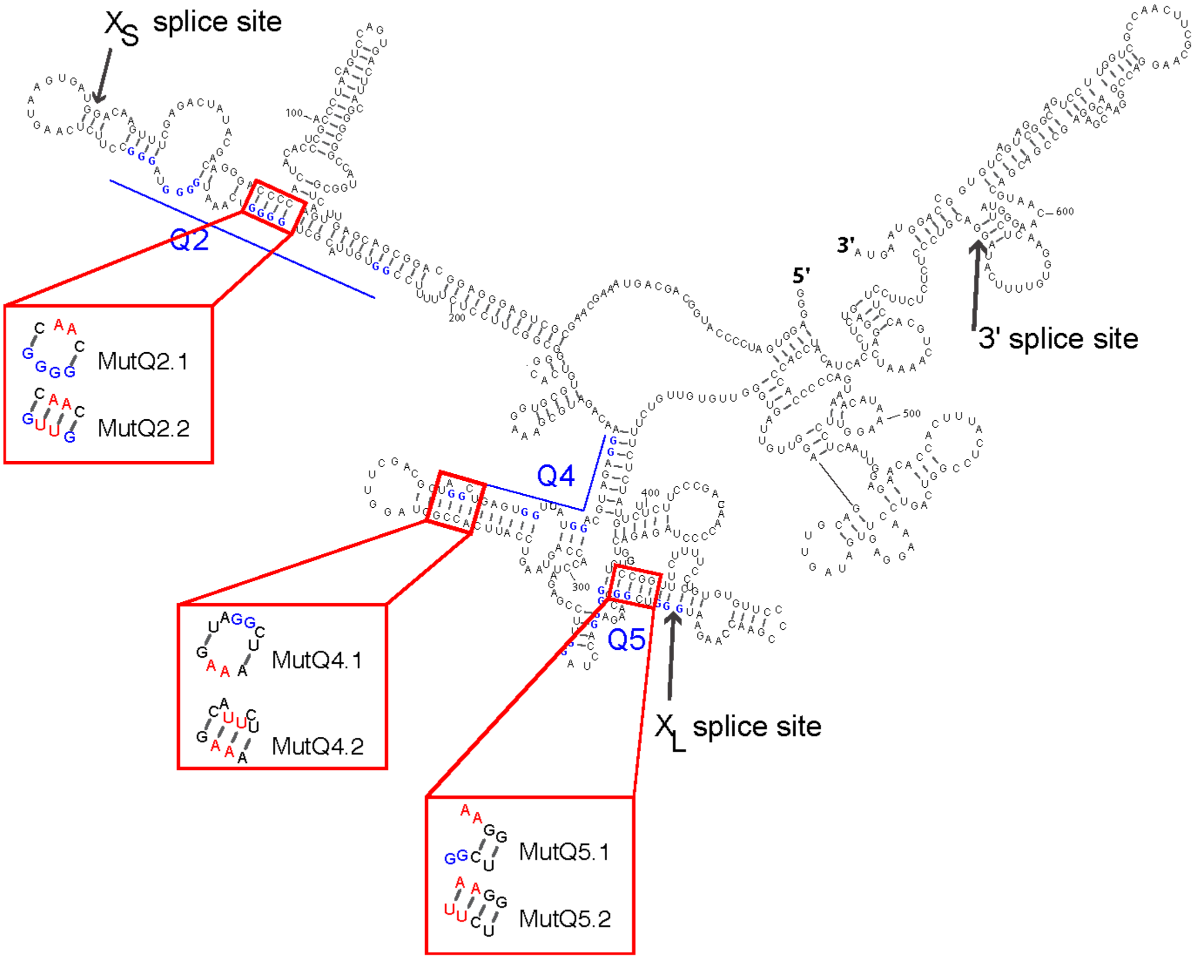

X_S splice site
Q2
Q4
Q5
5'
3'
3' splice site
X_L splice site
100
200
300
400
500
600
MutQ2.1
MutQ2.2
MutQ4.1
MutQ4.2
MutQ5.1
MutQ5.2

A

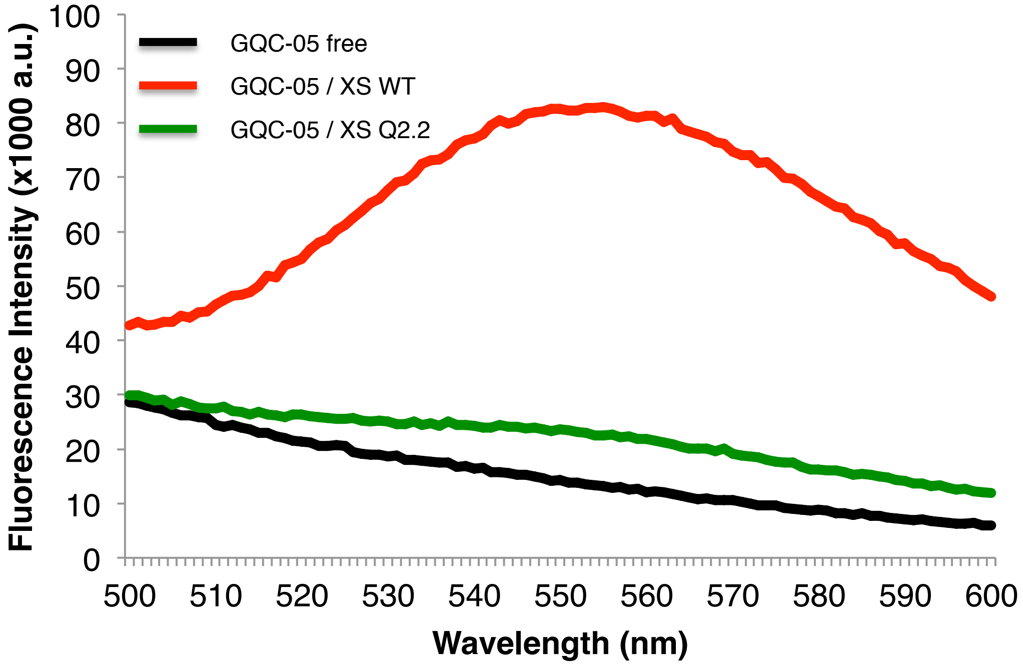

Fluorescence Intensity (x1000 a.u.)
Wavelength (nm)
GQC-05 free
GQC-05 / XS WT
GQC-05 / XS Q2.2
0
10
20
30
40
50
60
70
80
90
100
500
510
520
530
540
550
560
570
580
590
600

B

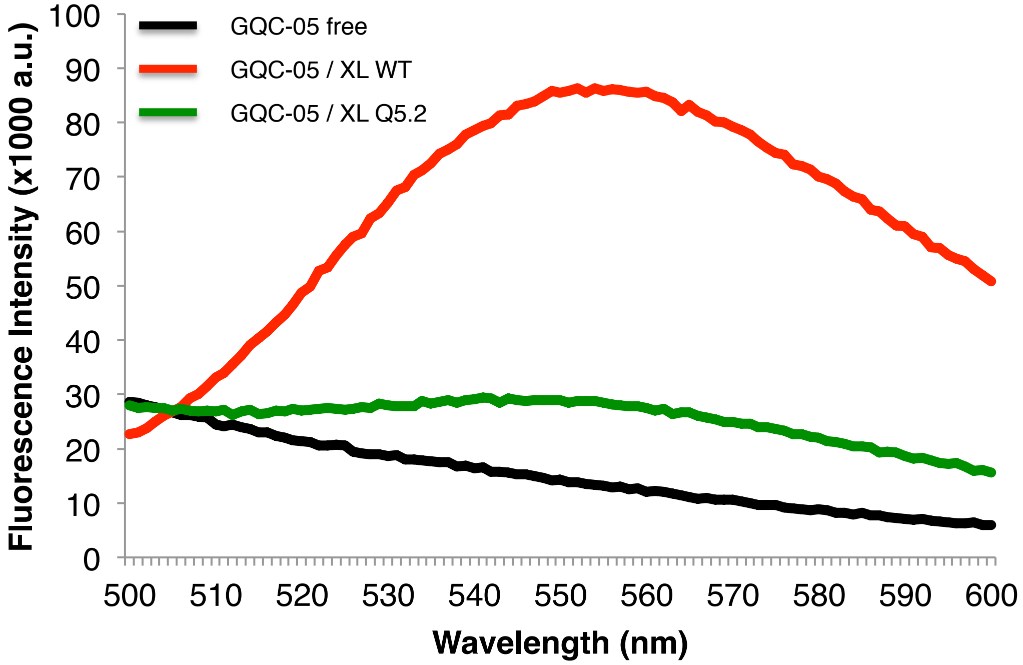

Fluorescence Intensity (x1000 a.u.)
Wavelength (nm)
GQC-05 free
GQC-05 / XL WT
GQC-05 / XL Q5.2
0
10
20
30
40
50
60
70
80
90
100
500
510
520
530
540
550
560
570
580
590
600

LEGENDS FOR SUPPLEMENTARY FIGURES

Supplementary Figure S1. Diagrammatic structures of six classes of G4-ligand.

Supplementary Figure S2. Diagrams showing the structures of GQC-05 and other ellipticines tested.

Supplementary Figure S3. Diagrams showing the structures of quindolines tested.

Supplementary Figure S4. Time courses of splicing complex assembly. Splicing reactions were incubated for the times shown, treated with heparin, then analysed by electrophoresis on native agarose gels. The reactions contained GQC-05 or an appropriate concentration of DMSO, the solvent for GQC-05.

Supplementary Figure S5. *In vitro* splicing of Bcl-X-681 in the presence of quindolines. Splicing assays were done in triplicate with the designated ligands at the concentrations shown (μM). The ligands were dissolved in 10% DMSO, and controls included samples done in the presence of DMSO at the same final concentration of 1%.

Supplementary Figure S6. Effects of G4 ligands on splicing of endogenous Bcl-X transcripts in HeLa cells. Three wells of cells were incubated in each case with the compound, dissolved in DMSO, at 10 μM for 4 h, together with three wells that were treated with the DMSO only. Incubation was followed by isolation of the RNA and amplification by reverse transcriptase-PCR. The products were separated by agarose gel electrophoresis and detected by ethidium bromide. Reactions done in parallel with reaction mixtures lacking reverse transcriptase were blank and have been omitted.

Supplementary Figure S7. Secondary structure of Bcl-X-681 pre-mRNA, showing the mutations introduced in the Q2, Q4 and Q5 regions.

Supplementary Figure S8. Fluorescence emission spectra of GQC-05 in the presence of domains of Bcl-X-681 carrying mutations intended to disrupt the potential to form G4s. RNA was incubated at 1 μM with GQC-05 at 20 μM. Excitation was at 321 nm. (A) Spectra of the X_S domain, wild-type (red) and mutant Q2.2 (green). Free GQC-05 is black. (B) Spectra of the X_L domain, wild-type (red)

and mutant Q5.2 (green). Free GQC-05 is black.
