# Supplementary material for: Specific G-quadruplex ligands modulate the alternative splicing of Bcl-X
Source: Nucleic Acids Res. 2017 Nov 16;46(2):886–96. doi: 10.1093/nar/gkx1122 (PMC5778605; doi:10.1093/nar/gkx1122)
Supplement: Supplementary Data [file gkx1122_supp.zip › nar-01717-f-2017-File004.pdf]

Supplementary Data: Effect of GQC-05 at 3 concentrations (1, 5, and 10 μM) on Bcl-x footprinting
Analysis of the data contained in gels displayed in Supplementary Figure 4 of Weldon et al. (2017), Nat. Chem. Biol. 13, 18-20.

Index is calculated as (V1(10μM GQC-05) / V1(no GQC-05)) / (T2(10μM GQC-05) / T2(no GQC-05))

Index values above and below the mean log(Index) ± 2SD are highlighted in Figure 5B

																		Index	Log(Index)
		Tris buffer				T1				T2				V1					
GQC-05 c°		-	1	5	10	-	1	5	10	-	1	5	10	-	1	5	10		
1	G	1.00	1.07	1.10	1.17	0.39	0.35	0.47	0.25	0.19	0.66	0.49	0.44	0.56	0.27	0.71	0.77	0.61	-0.21
2	G																	-	-
3	G	1.00	0.59	1.06	2.11	1.03	1.66	1.01	0.97	0.25	0.99	1.02	0.85	-0.30	0.75	-0.05	-0.31	0.31	-0.52
4	A																	-	-
5	G																	-	-
6	G	1.00	0.00	0.89	0.52	0.77	2.65	2.19	1.91	1.27	1.76	1.24	0.41	0.70	1.75	0.82	0.23	1.01	0.00
7	T	1.00	1.12	0.39	0.71	2.26	1.78	2.20	1.47	0.90	1.11	0.71	0.69	0.53	0.68	0.74	0.62	1.53	0.19
8	G	1.00	0.79	0.96	0.60	0.43	2.16	2.80	1.75	1.31	2.76	1.85	1.72	0.83	0.32	0.92	0.91	0.84	-0.08
9	A	1.00	0.50	0.67	0.71	5.69	5.35	2.74	2.13	0.88	0.76	1.41	0.55	0.49	0.40	0.39	0.49	1.61	0.21
10	T	1.00	0.69	0.58	0.46	0.00	0.18	0.58	1.24	0.97	1.39	0.70	0.65	0.50	0.25	0.35	0.46	1.36	0.14
11	C	1.00	0.74	0.69	0.49	0.25	0.60	0.58	0.53	0.32	0.58	0.85	0.71	0.42	0.51	0.56	0.63	0.68	-0.17
12	C	1.00	0.44	1.11	0.71	0.35	1.12	1.01	0.89	0.30	0.89	0.49	0.36	0.93	0.74	0.87	0.93	0.82	-0.09
13	C	1.00	1.28	1.12	1.75	1.10	0.47	0.90	0.38	0.20	0.28	0.19	0.19	0.80	0.33	0.55	0.27	0.34	-0.46
14	C	1.00	1.18	0.85	1.25	0.44	0.61	0.31	0.53	1.18	1.19	1.20	1.05	0.49	0.27	0.87	1.08	2.49	0.40
15	A	1.00	0.00	1.01	0.69	0.60	0.65	0.51	0.88	0.20	0.27	3.30	1.03	0.66	0.96	1.28	1.07	0.31	-0.50
16	T	1.00	0.91	0.94	0.55	0.79	1.09	0.95	0.77	0.51	1.18	1.26	1.15	0.37	1.07	0.87	0.46	0.55	-0.26
17	G	1.00	0.08	1.35	0.78	1.52	2.29	2.36	1.80	1.31	2.16	1.09	2.32	0.60	1.28	1.26	1.50	1.42	0.15
18	G	1.00	1.06	0.91	0.73	1.19	1.16	1.28	0.61	0.71	1.04	1.16	1.32	0.70	0.84	0.63	0.75	0.58	-0.24
19	C	1.00	0.47	0.99	1.25	2.31	2.17	1.43	1.21	0.48	0.86	0.78	0.70	0.62	0.24	0.69	0.64	0.71	-0.15
20	A	1.00	0.87	1.11	1.27	0.59	0.43	0.40	0.40	1.79	0.82	0.83	0.90	0.60	0.39	0.75	0.77	2.54	0.40
21	G	1.00	2.83	1.11	0.36	1.08	1.81	2.13	1.22	1.74	3.22	2.89	3.00	1.73	1.65	2.39	1.92	0.65	-0.19
22	C	1.00	1.2	1.2	1.4	2.06	1.85	1.31	1.10	1.26	1.54	1.78	0.94	0.9	1.0	0.6	0.6	0.84	-0.08
23	A	1.00	1.52	1.09	0.90	0.50	0.68	0.57	0.54	1.83	1.14	1.04	1.09	1.29	0.76	1.31	1.20	1.56	0.19
24	G	1.00	0.18	1.05	0.89	3.45	2.21	3.44	1.72	2.80	3.85	3.48	2.83	2.25	1.65	2.58	2.49	1.10	0.04
25	T	1.00	0.86	0.99	0.97	2.19	2.54	1.52	1.46	0.59	1.05	0.99	0.82	0.29	0.45	0.57	0.35	0.85	-0.07
26	A	1.00	1.68	1.26	1.17	1.03	0.62	0.87	0.97	2.89	1.68	1.16	1.29	1.21	0.73	1.08	1.59	2.92	0.47
27	A	1.00	0.63	1.21	0.87	0.96	1.16	1.15	1.14	5.99	8.39	9.38	8.98	1.49	1.46	1.31	1.41	0.63	-0.20
28	A	1.00	0.20	0.54	0.55	0.49	1.24	1.11	1.22	3.59	4.60	4.40	3.85	0.96	0.85	0.54	0.59	0.58	-0.24
29	G	1.00	1.18	1.21	1.03	6.92	2.88	5.50	2.51	6.57	12.60	8.16	9.27	0.97	0.94	1.55	1.33	0.97	-0.01
30	C	1.00	1.55	1.10	1.42	1.28	1.49	1.01	0.87	0.78	0.34	1.16	0.21	0.32	0.34	0.29	0.20	2.19	0.34
31	A	1.00	1.60	1.03	0.83	1.06	0.52	0.63	0.70	3.35	1.21	1.42	1.11	1.08	0.52	1.35	1.40	3.93	0.59
32	A	1.00	0.59	0.82	0.93	0.30	0.52	0.40	0.58	1.28	1.69	1.69	2.38	0.98	0.56	1.31	1.09	0.60	-0.22
33	G	1.00	0.12	0.60	0.16	3.86	2.47	3.80	2.55	2.69	3.80	5.16	6.78	0.44	0.89	0.16	0.35	0.32	-0.50
34	C	1.00	0.51	0.68	0.78	3.02	2.65	2.35	1.97	0.42	0.65	0.65	0.66	0.71	0.75	0.66	0.72	0.63	-0.20
35	G	1.00	0.70	0.72	0.91	0.50	0.67	0.45	0.61	0.48	0.58	0.63	0.37	0.45	0.35	0.54	0.61	1.81	0.26
36	C	1.00	0.73	0.79	0.72	0.97	0.99	0.71	0.81	0.43	0.62	0.39	0.36	1.18	1.05	1.08	1.00	1.03	0.01
37	T	1.00	0.61	1.05	1.00	0.42	0.54	0.79	0.66	0.42	0.71	0.56	0.67	0.66	0.81	0.74	0.72	0.69	-0.16
38	G	1.00	0.97	0.79	1.04	0.69	0.57	0.47	0.59	0.61	0.63	0.64	0.54	0.69	0.61	0.84	1.06	1.75	0.24
39	A	1.00	0.81	0.88	0.85	0.71	1.02	0.98	0.93	0.52	0.54	0.31	0.35	0.78	0.45	0.73	0.66	1.25	0.10
40	G	1.00	0.69	0.81	0.65	0.34	0.63	0.63	0.58	1.00	1.19	1.37	1.29	0.44	0.53	0.60	0.53	0.93	-0.03
41	G	1.00	0.64	0.94	1.30	0.87	1.11	1.16	1.07	0.83	1.02	0.94	0.68	0.91	1.05	0.77	1.04	1.41	0.15
42	G	1.00	0.82	0.98	0.95	0.73	0.69	1.04	0.61	0.59	0.69	0.50	0.66	0.39	0.37	0.52	0.58	1.31	0.12
43	A	1.00	1.20	1.04	1.29	0.59	0.60	0.43	0.43	0.36	0.49	0.46	0.41	0.40	0.18	0.54	0.60	1.30	0.12
44	G	1.00	0.78	1.27	1.68	0.78	0.75	1.24	0.65	1.06	1.11	0.99	0.93	0.74	0.72	0.92	1.00	1.53	0.19
45	G	1.00	1.14	1.05	1.19	0.67	0.61	0.90	0.63	0.47	0.70	0.62	0.56	0.42	0.32	0.63	0.61	1.23	0.09
46	C	1.00	0.89	1.40	0.98	2.22	2.49	1.93	1.81	1.33	1.56	1.14	1.26	0.62	0.59	0.99	0.94	1.61	0.21
47	A	1.00	1.31	0.97	1.13	0.62	0.43	0.29	0.39	1.55	0.67	0.66	0.76	0.63	0.33	0.68	0.73	2.37	0.38
48	G	1.00	0.45	0.74	0.79	0.46	0.74	0.88	0.93	0.75	1.76	1.56	1.59	2.34	2.26	2.32	2.09	0.42	-0.38
49	G	1.00	1.12	1.44	0.87	1.44	1.31	1.27	1.58	0.77	1.17	0.65	0.65	2.32	2.44	2.78	2.57	1.29	0.11
50	C	1.0	0.8	0.9	1.0	0.82	0.95	0.77	0.87	0.55	0.77	0.85	0.84	0.92	0.80	1.06	1.21	0.86	-0.07
51	G	1.00	1.08	1.20	1.47	0.47	0.28	0.41	0.38	0.55	0.54	0.52	0.52	1.01	0.79	1.24	1.13	1.18	0.07
52	A	1.00	0.32	0.64	0.62	2.14	2.53	1.77	1.50	0.78	1.28	0.76	1.15	2.02	2.06	2.86	3.13	1.05	0.02
53	C	1.00	1.52	0.97	0.96	0.91	1.28	0.93	1.28	1.31	1.30	1.45	1.72	1.85	1.68	1.68	1.44	0.59	-0.23
54	G	1.00	1.21	1.06	1.34	1.18	1.29	2.14	1.66	1.24	1.09	0.82	1.08	1.82	1.62	2.39	2.02	1.27	0.10
55	A	1.00	0.96	1.00	0.96	0.80	1.17	0.36	0.63	0.60	0.91	0.65	0.77	1.09	0.87	1.06	1.00	0.71	-0.15
56	G	1.00	0.84	1.02	1.12	1.52	0.83	2.07	1.35	3.11	3.12	2.86	3.29	0.79	0.72	0.78	0.81	0.97	-0.01
57	T	1.00	1.24	1.08	1.24	2.92	3.96	2.08	2.06	0.82	0.99	0.70	0.94	0.75	0.71	0.85	0.84	0.99	-0.01
58	T	1.00	0.93	1.01	1.03	1.15	1.74	1.63	1.85	2.49	2.92	2.25	2.65	1.25	1.18	1.23	1.26	0.95	-0.02
59	T	1.00	2.68	2.22	2.96	2.77	4.52	5.45	4.32	5.64	6.28	4.62	5.71	5.47	6.06	7.64	5.85	1.06	0.02
60	G	1.00	0.84	0.93	0.72	0.41	0.54	0.78	0.65	1.16	1.28	0.95	1.08	0.68	0.46	0.53	0.66	1.05	0.02
61	A	1.00	2.20	2.39	5.32	67.29	71.24	48.40	58.51	15.44	18.57	16.92	25.12	10.78	10.73	10.10	9.43	0.54	-0.27
62	A	1.00	0.60	0.97	1.00	0.46	0.43	0.59	1.07	1.52	1.72	1.59	1.93	0.69	0.68	0.77	0.79	0.91	-0.04
63	C	1.00	1.16	1.47	1.09	1.37	2.04	1.06	2.11	2.63	2.99	2.28	2.78	3.09	2.48	3.16	1.82	0.56	-0.25
64	T	1.00	0.82	0.76	0.62	0.27	0.55	0.44	0.35	0.33	0.32	0.27	0.32	0.49	0.50	0.60	0.78	1.65	0.22
65	G	1.00	0.73	1.40	1.12	3.87	3.67	3.87	3.22	2.30	2.94	2.24	2.67	2.91	2.59	3.48	3.48	1.03	0.01

66	C	1.00	1.80	2.74	2.76	1.64	1.79	3.34	2.21	0.82	0.76	0.59	0.89	1.68	1.24	2.26	2.12	1.16	0.06
67	G	1.00	0.87	0.92	0.64	0.73	0.76	0.63	0.57	0.93	0.97	0.90	0.92	0.56	0.30	0.51	0.65	1.18	0.07
68	G	1.00	1.17	1.21	1.29	1.96	2.33	2.88	2.21	1.48	1.68	1.50	1.73	1.01	0.94	1.19	1.14	0.96	-0.02
69	T	1.00	1.24	1.00	1.05	6.06	5.06	3.50	3.93	1.60	1.65	1.38	1.71	1.04	0.93	0.84	0.98	0.89	-0.05
70	A	1.00	1.22	1.46	1.15	0.61	0.62	0.50	0.80	1.90	1.41	1.26	1.41	0.67	0.55	0.66	0.82	1.65	0.22
71	C	1.00	1.08	0.91	0.93	0.49	0.50	1.05	0.93	4.41	4.42	4.32	4.21	1.07	0.98	0.81	1.05	1.03	0.01
72	C	1.00	1.16	0.97	0.86	0.58	0.57	0.89	0.92	1.14	0.77	0.76	0.87	0.90	0.84	0.76	0.97	1.40	0.15
73	G	1.00	0.72	0.95	1.06	0.64	0.44	0.33	0.41	0.66	0.64	0.64	0.61	0.55	0.48	0.62	0.72	1.39	0.14
74	G	1.00	1.38	1.32	1.19	1.41	1.92	3.93	4.02	1.26	1.21	1.21	1.27	1.26	1.45	1.19	1.38	1.08	0.03
75	C	1.00	0.72	0.75	0.92	0.62	0.54	0.32	0.55	0.29	0.29	0.34	0.33	0.34	0.31	0.39	0.40	1.03	0.01
76	G	1.00	0.89	1.06	0.92	0.61	0.75	0.72	0.79	0.82	0.84	0.88	0.97	1.15	1.24	1.08	1.06	0.77	-0.11
77	G	1.00	0.96	0.72	0.79	1.13	0.97	0.77	0.94	1.06	0.96	1.13	1.21	1.93	2.19	1.67	1.53	0.70	-0.16
78	G	1.00	0.78	0.92	0.75	1.04	1.41	1.85	1.09	1.01	0.95	1.00	1.05	0.46	0.58	0.55	0.59	1.23	0.09
79	C	1.00	1.27	0.82	1.05	0.99	1.19	1.05	0.82	1.12	1.19	1.38	1.38	0.71	0.56	0.91	0.80	0.91	-0.04
80	A	1.00	1.25	0.98	0.98	0.80	1.04	0.86	0.81	1.02	0.67	0.72	0.77	0.52	0.41	0.71	0.65	1.67	0.22
81	T	1.00	0.95	0.94	0.89	0.60	0.35	0.31	0.72	1.25	1.10	1.27	1.25	1.61	1.91	1.76	1.30	0.80	-0.10
82	T	1.00	0.84	0.99	0.89	0.60	0.65	0.58	0.86	0.96	0.91	1.18	1.34	1.22	1.40	1.17	1.08	0.63	-0.20
83	C	1.00	1.36	1.45	1.29	0.62	0.65	0.68	1.02	1.59	1.44	1.78	1.73	6.21	7.53	4.82	4.29	0.64	-0.20
84	A	1.00	1.14	1.10	0.73	0.56	0.34	0.62	0.59	1.09	0.35	0.38	0.49	0.59	0.47	0.47	0.56	2.09	0.32
85	G																	-	-
86	T	1.00	1.42	0.95	0.55	4.45	4.88	4.24	5.27	0.61	0.38	0.63	0.80	2.01	2.63	1.42	1.48	0.56	-0.25
87	G	1.00	0.88	1.05	0.76	0.31	0.23	0.46	0.29	1.39	1.05	1.36	1.86	0.80	0.87	0.65	0.79	0.74	-0.13
88	A	1.00	0.77	0.88	1.03	6.17	6.24	5.76	8.50	0.68	0.63	0.88	0.65	1.07	1.39	0.78	1.08	1.07	0.03
89	C	1.00	0.72	0.98	0.87	0.57	0.65	0.27	0.46	1.63	1.50	1.60	1.68	0.80	1.13	0.81	0.80	0.97	-0.01
90	C	1.00	1.28	0.98	0.79	0.47	0.31	0.94	0.93	1.33	0.99	1.03	1.27	8.11	10.53	6.77	5.91	0.77	-0.12
91	T	1.00	0.84	1.02	1.09	0.74	0.52	0.33	0.31	0.39	0.41	0.54	0.51	3.13	4.31	2.66	2.56	0.61	-0.21
92	G	1.00	0.71	0.92	0.84	1.19	0.90	1.31	1.77	0.45	0.65	0.57	0.52	0.84	1.10	0.62	0.67	0.68	-0.16
93	A																	-	-
94	C	1.00	1.32	1.06	1.17	1.12	0.97	1.13	1.18	0.91	0.88	1.06	1.19	1.65	1.07	1.94	1.56	0.73	-0.14
95	A	1.00	0.39	0.41	0.21	0.02	-0.10	0.10	0.11	0.31	1.13	0.70	0.38	-0.54	-0.57	-0.73	-0.55	0.84	-0.08
96	T	1.00	1.08	1.25	1.14	1.05	1.19	0.96	1.27	1.42	0.93	0.94	1.18	1.36	1.14	1.74	1.66	1.47	0.17
97	C	1.00	1.19	1.10	1.15	1.20	1.05	1.15	0.93	0.83	0.53	0.56	0.32	0.73	1.29	0.85	0.81	2.92	0.47
98	C	1.00	1.12	1.01	1.03	0.98	0.73	0.56	0.64	0.70	0.74	0.65	0.73	0.54	0.48	0.87	1.09	1.93	0.29
99	C	1.00	0.98	1.05	1.24	0.73	0.64	0.54	0.59	0.92	0.77	0.74	0.70	0.80	0.32	0.85	0.85	1.40	0.14
100	A	1.00	0.83	0.79	0.80	0.53	0.34	0.59	0.63	0.46	0.58	0.45	0.68	0.66	0.35	0.83	0.84	0.87	-0.06
101	G	1.00	1.05	0.84	0.69	0.65	0.89	0.73	0.57	0.88	0.55	0.83	0.84	1.39	0.98	1.82	1.72	1.30	0.11
102	C	1.00	0.84	1.39	1.06	0.98	1.09	1.04	0.78	0.75	0.37	1.21	0.77	1.11	1.25	0.87	1.42	1.25	0.10
103	T	1.00	0.71	0.98	0.53	0.56	0.35	0.87	1.04	0.62	0.67	0.65	0.48	0.70	0.39	0.93	0.67	1.25	0.10
104	C	1.00	1.65	1.12	1.93	0.67	1.04	0.92	1.33	1.45	1.50	1.06	0.67	3.76	2.33	6.10	3.33	1.92	0.28
105	C	1.00	1.47	0.87	1.54	0.93	0.79	0.45	0.87	0.88	1.25	1.31	1.33	1.34	0.92	1.26	1.47	0.72	-0.14
106	A	1.00	1.11	0.91	0.97	0.75	0.69	0.66	0.93	1.78	1.00	1.19	0.96	0.89	0.73	1.15	2.08	4.33	0.64
107	C	1.00	0.65	0.69	0.68	0.65	0.42	0.56	0.28	0.33	0.33	0.26	0.33	0.35	0.35	0.42	0.15	0.41	-0.38
108	A	1.00	1.11	0.82	1.00	0.59	0.97	0.65	0.91	0.82	0.90	1.13	1.15	1.25	0.47	1.47	1.77	1.01	0.00
109	T	1.00	0.76	0.93	0.69	0.64	0.26	0.61	0.47	0.52	0.44	0.51	0.34	0.39	0.45	0.41	0.43	1.71	0.23
110	C	1.00	1.00	0.89	0.98	0.77	0.72	0.83	0.63	0.65	0.31	0.43	0.57	0.66	0.64	0.72	0.63	1.10	0.04
111	A	1.00	0.73	0.76	0.83	0.60	0.64	0.53	0.47	0.66	0.53	0.57	0.61	0.55	0.51	0.65	0.86	1.68	0.23
112	C	1.00	1.16	1.07	1.05	0.68	0.84	0.85	1.39	0.52	0.87	0.86	0.89	0.85	0.71	0.54	1.03	0.70	-0.15
113	C	1.00	1.25	1.48	1.56	1.50	1.10	1.33	0.88	1.16	0.93	1.06	0.93	0.56	0.70	1.31	0.95	2.12	0.33
114	C	1.00	0.70	0.85	1.01	0.78	0.52	0.66	0.59	0.72	0.67	0.65	0.64	0.69	0.39	0.94	0.81	1.31	0.12
115	C	1.00	0.81	0.76	0.75	0.67	0.50	0.66	0.83	0.48	0.48	0.58	0.38	0.73	0.59	0.88	0.78	1.37	0.14
116	A	1.00	0.78	0.83	0.58	0.59	0.75	0.70	0.82	0.93	0.58	0.53	0.63	0.86	0.51	0.91	0.96	1.64	0.22
117	G	1.00	1.63	1.24	1.37	1.01	1.28	1.16	0.67	3.11	3.29	4.05	4.71	1.12	1.20	1.49	1.26	0.74	-0.13
118	G	1.00	1.29	1.06	1.33	2.62	2.91	2.56	2.39	0.90	0.84	0.74	1.05	0.54	0.98	0.37	0.77	1.23	0.09
119	G	1.00	1.14	0.99	0.99	1.47	1.77	1.66	1.44	0.90	0.86	1.03	0.77	0.49	0.49	0.60	0.65	1.53	0.19
120	A	1.00	0.68	0.81	0.97	0.97	0.79	0.81	0.82	0.32	0.37	0.46	0.36	0.47	0.25	0.75	0.80	1.49	0.17
121	C	1.00	0.85	1.16	0.95	0.80	0.82	0.93	0.94	0.92	1.01	1.33	1.10	1.32	0.87	1.43	1.05	0.66	-0.18
122	A	1.00	0.70	0.62	0.75	0.17	0.18	0.20	0.23	1.32	0.44	0.60	0.56	0.82	0.41	0.85	1.00	2.90	0.46
123	G	1.00	1.55	1.43	1.16	1.05	1.43	0.82	1.09	3.01	3.58	4.37	4.49	1.16	1.57	1.24	1.09	0.63	-0.20
124	C	1.00	1.0	0.8	0.9	4.52	4.30	4.10	3.33	0.36	0.78	0.49	0.74	0.69	0.46	0.92	0.78	0.56	-0.25
125	A	1.00	0.66	0.59	0.50	0.22	0.13	0.19	0.26	0.99	0.37	0.41	0.49	0.41	0.29	0.50	0.70	3.48	0.54
126	T	1.00	1.05	0.87	0.89	0.64	0.75	0.64	1.04	1.13	1.56	1.65	1.58	1.69	1.08	1.63	1.03	0.44	-0.36
127	A	1.00	1.03	1.40	1.55	0.98	0.78	0.83	0.91	1.50	0.78	1.04	0.96	0.71	0.68	1.17	1.46	3.19	0.50
128	T	1.00	1.37	1.28	1.16	1.17	1.14	0.98	0.96	2.84	3.14	3.44	3.41	0.77	1.18	1.00	0.77	0.84	-0.08
129	C	1.00	0.94	1.18	0.71	0.67	0.83	1.01	0.99	0.85	1.00	1.11	1.04	0.86	0.96	0.95	0.96	0.90	-0.05
130	A	1.00	0.50	0.51	0.61	0.35	0.28	0.37	0.28	0.87	0.36	0.41	0.41	0.46	0.20	0.59	0.64	2.98	0.47
131	G	1.00	1.32	1.17	1.21	0.71	1.12	0.84	0.71	3.00	3.08	3.42	3.60	1.11	1.05	1.51	1.34	1.01	0.00
132	A	1.00	0.87	1.42	1.34	6.62	6.14	5.49	6.06	0.76	0.87	0.77	1.16	0.98	1.39	1.39	1.03	0.68	-0.17
133	G	1.00	1.06	1.13	0.92	0.38	0.71	0.72	1.46	2.07	1.98	2.20	1.93	0.99	1.16	1.03	0.81	0.88	-0.06
134	C	1.00	0.79	0.87	1.10	1.28	1.02	0.92	0.63	0.49	0.53	0.58	0.54	0.48	0.47	0.66	0.77	1.44	0.16
135	T	1.00	0.79	0.95	1.13	0.78	0.91	0.84	1.00	0.91	1.02	0.85	0.80	1.31	0.86	1.63	1.40	1.20	0.08
136	T	1.00	0.98	0.97	1.12	0.92	0.68	0.83	1.04	1.23	1.10	1.03	1.13	3.64	2.23	4.20	3.50	1.05	0.02
137	T	1.00	1.32	1.03	1.22	1.05	0.96	1.11	1.08	1.14	0.65	0.95	1.18	0.78	1.10	0.85	0.72	0.90	-0.05
138	G	1.00	1.06	0.97	1.12	0.77	0.87	0.96	0.73	1.76	1.88	2.26	2.21	0.73	0.74	0.78	0.79	0.86	-0.06
139	A	1.00	1.07	0.90	1.15	4.94	5.14	5.01	4.68	1.13	1.28	1.19	1.21	0.61	0.82	0.81	0.80	1.22	0.09
140	A	1.00	0.93	0.89	1.39	1.09	0.51	0.88	0.81	1.54	1.68	1.90	1.95	0.68	0.92	0.94	0.79	0.92	-0.04
141	C	1.00	1.11	1.29	1.33	0.70	0.57	0.87	0.84	1.58	1.69	1.65	1.86	0.88	1.05	1.10	1.03	0.99	0.00

142	A	1.00	0.62	0.72	0.63	0.40	0.32	0.37	0.32	1.10	0.31	0.38	0.42	0.42	0.31	0.43	0.61	3.76	0.57
143	G	1.00	1.11	1.19	1.28	1.11	1.02	0.84	1.05	1.34	1.31	1.59	1.70	4.08	2.73	4.04	2.93	0.56	-0.25
144	G	1.00	1.06	1.09	1.42	1.61	1.86	1.80	1.49	0.95	1.49	1.40	1.39	0.65	0.96	0.65	0.68	0.72	-0.15
145	T	1.00	1.13	1.05	1.69	2.15	1.94	2.00	1.97	0.99	0.82	0.74	0.76	0.73	0.82	0.83	0.77	1.38	0.14
146	A	1.00	0.79	0.88	0.97	0.68	0.54	0.58	0.63	1.49	0.63	0.66	0.69	0.88	0.73	0.94	1.20	2.94	0.47
147	G	1.00	0.92	1.19	1.39	0.86	0.68	0.82	0.94	1.88	2.01	2.33	2.40	1.23	1.35	0.99	1.12	0.71	-0.15
148	T	1.00	1.02	0.89	1.25	1.97	2.12	2.24	1.75	0.74	0.76	0.78	0.75	0.76	0.81	0.67	0.69	0.90	-0.04
149	G	1.00	1.03	0.92	1.27	0.72	0.60	0.75	0.73	1.02	0.99	0.97	1.01	1.46	1.07	1.26	0.99	0.68	-0.17
150	A	1.00	1.04	1.01	1.30	1.91	1.88	1.60	1.51	0.74	0.76	0.66	0.78	1.01	0.93	0.97	0.98	0.92	-0.03
151	A	1.00	1.21	1.12	1.40	0.92	0.90	1.01	1.04	1.24	1.34	1.29	1.38	0.70	0.87	0.67	0.78	0.99	0.00
152	T	1.0	1.0	1.0	1.0	0.83	0.77	0.73	0.93	1.21	1.20	1.23	1.26	1.77	1.27	1.28	1.26	0.69	-0.16
153	G	1.00	0.88	1.15	1.32	0.75	0.68	0.72	0.73	1.19	1.39	1.53	1.42	1.30	1.05	1.23	1.18	0.76	-0.12
154	A	1.00	0.88	0.98	1.06	3.02	3.16	2.45	2.29	0.66	0.62	0.69	0.77	0.70	0.70	0.77	0.76	0.95	-0.02
155	A	1.00	0.95	1.20	1.23	0.97	0.85	0.70	0.91	3.74	4.72	5.02	4.78	0.74	0.76	0.77	0.83	0.88	-0.06
156	C	1.00	1.07	1.11	1.11	0.78	0.61	0.94	0.99	2.44	2.88	2.89	2.70	0.83	0.91	0.76	0.79	0.86	-0.07
157	T	1.00	0.92	0.99	0.99	0.92	0.66	0.92	0.93	0.74	0.82	0.82	0.73	0.78	0.63	0.69	0.78	1.01	0.01
158	C	1.00	1.05	1.13	1.13	0.93	0.91	1.06	1.05	0.82	0.83	0.79	0.82	1.37	1.31	1.37	1.48	1.07	0.03
159	T	1.00	0.98	0.86	0.91	0.76	0.62	0.70	0.74	0.65	0.74	0.74	0.74	0.58	0.64	0.70	0.72	1.08	0.03
160	T	1.00	0.82	0.97	1.03	0.82	0.94	0.84	0.92	1.16	1.33	1.34	1.29	0.78	0.73	0.63	0.77	0.88	-0.05
161	C	1.00	1.16	1.26	1.56	1.13	1.02	1.02	0.96	0.69	0.84	0.84	0.95	0.87	0.90	0.61	0.80	0.66	-0.18
162	C	1.00	0.87	1.21	0.65	0.77	0.57	0.67	0.67	1.03	0.76	0.68	0.53	1.21	0.98	1.04	1.05	1.69	0.23
163	G	1.00	0.83	0.89	1.03	0.99	0.87	1.09	1.20	0.85	1.14	1.13	1.34	3.23	3.04	3.17	3.05	0.60	-0.22
164	G	1.00	0.95	1.15	1.06	1.12	1.10	1.17	1.26	0.91	1.33	1.22	1.16	2.43	2.06	2.03	1.87	0.60	-0.22
165	G	1.00	0.96	1.13	0.98	1.32	1.25	1.08	1.19	0.89	1.18	1.05	1.05	0.74	0.61	0.71	0.79	0.90	-0.05
166	A	1.00	0.91	1.07	1.05	2.30	2.38	1.95	1.97	0.69	0.93	0.81	0.86	0.76	0.70	0.57	0.72	0.77	-0.11
167	T	1.00	1.33	1.30	0.76	0.64	0.56	0.47	0.53	0.74	1.05	0.99	0.85	0.45	0.53	0.45	0.55	1.06	0.03
168	G	1.00	0.99	0.97	1.25	1.16	1.17	1.19	1.23	1.27	1.90	1.46	1.45	1.25	1.42	1.24	1.32	0.93	-0.03
169	G	1.00	1.39	1.24	1.46	2.06	2.04	1.87	1.70	1.58	2.34	1.95	1.65	0.99	0.90	0.79	0.93	0.89	-0.05
170	G	1.00	1.16	1.07	1.17	1.64	1.71	1.46	1.37	1.28	1.91	1.67	1.36	0.83	0.77	0.75	0.84	0.95	-0.02
171	G	1.00	0.84	0.86	0.87	1.50	1.82	1.49	1.21	1.29	1.95	1.51	1.17	0.71	0.60	0.72	0.85	1.32	0.12
172	T	1.00	0.84	0.97	0.87	1.04	1.12	0.91	0.78	0.55	0.72	0.66	0.61	0.64	0.41	0.72	0.84	1.18	0.07
173	A	1.00	0.95	0.93	0.82	0.56	0.53	0.57	0.57	0.82	0.71	0.69	0.62	0.70	0.58	0.70	0.78	1.48	0.17
174	A	1.00	1.11	1.25	1.18	1.01	0.87	1.06	1.13	1.40	1.79	1.67	1.38	1.10	1.42	1.03	1.04	0.96	-0.02
175	A	1.00	1.19	1.06	1.31	1.01	1.00	1.08	1.13	1.13	1.27	1.39	1.33	0.83	0.94	0.76	0.90	0.91	-0.04
176	C	1.00	1.08	1.06	1.23	0.94	0.87	0.99	0.95	1.19	1.28	1.37	1.28	0.80	0.86	0.69	0.83	0.96	-0.02
177	T	1.00	1.10	1.15	1.23	0.86	0.83	1.02	0.95	0.87	0.77	0.79	0.85	0.75	0.82	0.91	0.86	1.17	0.07
178	G	1.00	0.94	1.09	1.10	0.75	0.74	0.82	0.82	0.61	0.60	0.68	0.59	0.68	0.64	0.76	0.74	1.14	0.06
179	G	1.00	0.74	0.83	0.71	0.94	1.04	0.95	1.12	0.63	0.71	0.83	0.83	0.84	0.63	0.89	0.80	0.73	-0.14
180	G	1.00	1.48	1.45	1.55	1.44	1.49	1.56	1.72	1.30	1.34	1.26	1.41	1.12	1.57	1.43	1.17	0.96	-0.02
181	G	1.00	1.01	1.05	1.03	0.79	0.87	0.81	0.81	0.71	0.84	0.86	0.83	1.13	1.28	1.17	1.10	0.84	-0.08
182	T	1.00	0.91	0.95	0.87	0.71	0.83	0.74	0.70	0.67	0.78	0.70	0.69	0.68	0.47	0.74	0.80	1.16	0.06
183	C	1.00	0.92	1.04	0.86	0.64	0.69	0.76	0.71	0.65	0.74	0.71	0.64	2.03	2.96	1.90	1.73	0.87	-0.06
184	G	1.00	1.17	1.07	1.08	0.65	0.75	0.66	0.69	0.85	1.02	0.93	0.87	2.32	3.21	1.90	1.64	0.69	-0.16
185	C	1.00	1.73	1.00	1.16	3.66	3.72	4.22	3.66	0.85	1.12	1.03	1.17	4.67	7.24	3.78	2.83	0.44	-0.36
186	A	1.00	1.21	0.91	0.88	0.56	0.49	0.56	0.51	1.29	0.59	0.53	0.58	0.59	0.45	0.54	0.71	2.67	0.43
187	T	1.00	1.46	1.03	1.38	0.90	1.02	1.17	1.17	3.37	4.24	4.12	4.34	3.08	4.79	2.64	2.30	0.58	-0.24
188	T	1.00	1.18	1.10	1.03	0.80	0.91	0.92	1.05	1.98	2.23	2.33	2.74	1.52	2.23	1.14	1.04	0.50	-0.30
189	G	1.00	1.11	0.98	1.29	0.72	0.85	0.79	0.84	1.72	1.78	1.82	2.14	1.01	1.27	0.81	0.77	0.61	-0.21
190	T	1.00	1.02	1.22	0.99	3.18	3.36	3.72	3.84	0.66	0.57	0.69	0.68	1.46	1.89	1.10	0.96	0.65	-0.19
191	G	1.00	1.07	1.04	1.08	0.49	0.48	0.57	0.54	1.11	0.99	1.06	1.19	1.41	1.69	1.09	1.00	0.66	-0.18
192	G	1.00	1.10	1.23	1.16	1.98	2.15	2.24	2.75	1.49	1.72	1.84	2.14	1.41	1.76	1.21	1.22	0.60	-0.22
193	C	1.00	0.82	0.96	0.67	1.34	1.54	1.48	1.85	0.65	0.75	0.80	0.81	0.98	0.69	0.90	1.00	0.82	-0.09
194	C	1.00	1.17	1.25	1.15	0.87	0.90	0.83	0.83	0.63	0.82	0.78	0.73	1.10	1.19	0.97	1.06	0.82	-0.09
195	T	1.00	1.27	1.22	1.22	1.28	1.27	1.42	1.25	1.15	1.20	1.39	1.18	1.14	1.04	1.12	1.28	1.10	0.04
196	T	1.00	1.39	1.48	1.33	0.92	0.92	1.07	0.99	1.01	0.98	1.36	1.06	1.04	0.91	0.95	1.14	1.05	0.02
197	T	1.00	1.10	0.97	0.98	0.93	0.80	0.98	0.97	0.83	0.99	1.10	1.02	0.80	0.70	0.78	0.85	0.86	-0.06
198	T	1.00	1.06	0.90	0.92	0.91	0.86	0.92	0.91	0.98	1.20	1.11	1.10	0.80	0.71	0.69	0.70	0.79	-0.10
199	T	1.00	0.81	0.93	0.94	0.89	0.85	0.85	0.80	0.96	1.08	1.04	1.02	0.80	0.67	0.66	0.67	0.78	-0.11
200	C	1.00	0.90	0.89	0.88	0.94	0.82	0.88	0.92	0.92	1.02	0.82	0.97	1.12	0.95	0.87	0.88	0.74	-0.13
201	T	1.00	0.88	1.14	1.12	1.27	1.12	1.30	1.11	1.30	1.52	1.13	1.27	1.12	1.08	0.97	1.04	0.95	-0.02
202	C	1.00	0.97	3.25	2.82	3.84	3.72	2.77	3.83	3.77	3.57	3.40	2.52	5.26	6.22	5.47	5.43	1.55	0.19
203	C	1.00	0.85	0.26	0.32	0.23	0.29	0.30	0.25	0.04	0.17	0.20	0.37	0.57	0.30	0.88	0.82	0.16	-0.79
204	T	1.00	1.19	1.06	1.06	0.62	0.55	0.74	0.62	0.68	0.90	0.65	0.94	1.07	0.57	0.16	0.23	0.15	-0.82
205	T	1.00	1.00	1.08	1.01	0.71	0.72	0.70	0.72	0.89	0.82	0.95	0.84	0.80	0.77	0.64	0.71	0.94	-0.03
206	C	1.00	0.85	0.94	0.84	0.99	0.85	0.95	0.91	0.91	0.85	0.91	0.98	1.47	1.43	1.37	1.51	0.96	-0.02
207	G	1.00	0.87	0.82	0.91	0.92	0.90	0.58	0.85	0.80	0.82	0.82	0.85	1.13	1.16	1.09	1.11	0.93	-0.03
208	G	1.00	0.78	0.73	0.83	1.26	1.12	1.39	1.29	1.00	0.73	0.92	0.93	0.79	0.80	0.93	0.83	1.14	0.06
209	C	1.00	1.15	1.43	1.50	1.37	1.29	1.42	1.43	1.04	0.98	1.18	1.38	0.86	0.87	0.85	1.06	0.93	-0.03
210	G	1.00	0.81	0.83	0.81	0.52	0.47	0.48	0.57	0.69	0.54	0.64	0.57	0.50	0.54	0.54	0.53	1.29	0.11
211	G	1.00	0.82	0.78	0.83	0.53	0.58	0.60	0.61	0.68	0.60	0.67	0.70	1.89	1.91	1.68	1.58	0.82	-0.08
212	G	1.00	0.79	0.95	0.94	0.99	0.99	1.25	1.11	1.09	0.89	1.05	1.06	0.88	0.86	0.97	1.07	1.26	0.10
213	G	1.00	1.36	1.47	1.57	1.78	1.92	2.15	1.97	2.17	1.75	2.27	2.06	1.67	1.59	1.79	1.88	1.18	0.07
214	C	1.00	1.07	1.34	1.27	1.68	2.04	2.14	1.89	1.86	1.49	1.83	1.62	1.30	1.33	1.53	1.53	1.35	0.13
215	A	1.00	0.98	1.13	0.99	0.99	1.14	0.78	0.88	0.76	0.62	0.72	0.67	0.71	0.73	0.93	0.83	1.30	0.11
216	C	1.00	1.25	1.30	1.10	0.85	1.32	1.14	1.12	1.63	1.34	1.72	1.62	3.02	2.99	2.71	2.52	0.84	-0.07
217	T	1.00	1.40	1.36	1.30	1.18	1.47	1.27	1.32	1.73	1.27	1.44	1.47	2.49	2.70	2.29	2.10	0.99	0.00

218	G	1.00	1.57	1.11	1.27	1.00	1.32	1.20	1.09	1.44	1.03	1.38	1.40	3.18	3.30	3.04	2.57	0.83	-0.08
219	T	1.00	1.31	1.27	1.21	1.71	2.49	2.33	2.04	1.37	0.95	1.17	1.07	1.17	1.36	1.13	1.14	1.24	0.09
220	G	1.00	1.74	1.11	1.18	0.87	1.08	0.90	0.94	1.38	1.29	1.28	1.35	1.57	1.49	1.37	1.16	0.76	-0.12
221	C	1.00	1.50	1.88	1.84	2.68	3.47	3.74	3.30	2.20	1.59	1.81	1.94	2.45	2.48	2.01	1.95	0.90	-0.05
222	G	1.00	1.39	1.24	0.97	1.08	1.00	1.24	1.00	1.23	1.00	0.95	0.96	1.35	1.60	1.40	1.28	1.21	0.08
223	T	1.00	1.44	1.11	1.41	1.92	2.65	2.11	2.32	1.79	1.31	1.38	1.52	5.03	5.37	5.52	5.93	1.39	0.14
224	G	1.00	0.31	1.00	1.37	2.09	2.11	3.11	3.34	2.63	2.43	3.18	2.97	4.47	4.42	3.82	4.34	0.86	-0.07
225	G	1.00	1.84	1.83	1.63	1.09	1.29	1.23	0.88	1.59	0.95	1.01	1.06	0.82	1.02	0.96	0.90	1.63	0.21
226	A	1.00	1.02	0.98	0.95	2.66	2.74	2.74	2.77	0.40	0.78	0.62	0.52	0.46	0.29	0.45	0.50	0.82	-0.09
227	A	1.00	1.0	1.0	1.0	0.46	0.51	0.57	0.54	0.84	0.77	1.03	0.96	1.1	0.5	0.9	1.0	0.76	-0.12
228	A	1.00	1.02	1.13	1.02	0.56	0.76	0.76	0.72	1.02	1.41	1.56	1.44	1.35	0.88	1.14	1.25	0.65	-0.19
229	G	1.00	1.01	1.00	0.91	0.80	1.33	0.91	1.20	2.06	2.21	2.12	1.90	0.78	0.52	0.63	0.83	1.16	0.06
230	C	1.00	1.09	0.97	1.01	2.87	2.93	2.70	2.40	1.07	1.06	1.04	0.93	1.10	0.60	0.98	1.14	1.20	0.08
231	G	1.00	1.11	1.09	1.35	0.79	1.41	0.94	1.14	1.09	1.02	0.96	0.96	1.06	0.63	0.88	0.95	1.02	0.01
232	T	1.00	1.08	1.13	1.22	4.64	4.22	4.15	3.73	1.51	1.30	0.99	1.04	2.36	1.46	1.93	2.16	1.32	0.12
233	A	1.00	0.99	1.07	1.10	0.51	0.28	0.37	0.41	1.31	0.43	0.60	0.65	0.61	0.23	0.58	0.91	3.01	0.48
234	G	1.00	1.16	1.29	1.27	1.67	1.92	2.09	1.78	4.08	3.64	4.05	3.95	1.01	0.95	0.95	0.97	0.99	0.00
235	A	1.00	0.84	0.96	0.91	6.18	5.76	5.00	5.62	1.73	1.50	1.08	0.96	0.87	0.62	0.68	0.75	1.54	0.19
236	C	1.00	1.09	0.89	1.11	0.91	0.81	0.83	0.73	1.83	1.73	1.89	1.97	1.10	0.82	0.97	1.15	0.97	-0.01
237	A	1.00	1.01	0.99	1.13	0.65	0.32	0.41	0.44	1.58	0.44	0.60	0.84	0.64	0.26	0.61	0.79	2.34	0.37
238	A	1.00	0.98	1.21	0.88	0.71	0.67	0.77	0.77	0.71	0.89	1.29	0.95	1.69	1.11	1.44	1.43	0.64	-0.20
239	G	1.00	0.86	0.95	0.80	0.64	0.73	0.77	0.82	1.22	1.06	1.46	1.25	1.69	1.07	1.39	1.43	0.83	-0.08
240	G	1.00	1.10	1.05	0.83	1.45	1.19	1.31	1.18	0.82	0.61	0.75	0.62	1.01	0.66	0.97	1.12	1.46	0.17
241	A	1.00	1.05	1.04	0.91	1.13	0.93	1.12	0.99	0.44	0.55	0.42	0.46	0.66	0.41	0.66	0.79	1.14	0.06
242	G	1.00	1.01	1.08	0.91	0.57	0.48	0.64	0.60	0.79	0.79	0.80	0.80	1.00	0.55	0.83	1.00	0.99	0.00
243	A	1.00	1.10	1.11	0.79	1.40	1.17	1.35	1.26	0.61	0.49	0.54	0.51	1.00	0.57	0.83	0.96	1.14	0.06
244	T	1.00	1.13	1.21	1.01	0.55	0.52	0.57	0.55	1.00	0.73	0.73	0.78	1.25	0.68	1.11	1.14	1.18	0.07
245	G	1.00	1.06	1.13	1.07	0.54	0.52	0.59	0.57	1.11	0.78	0.82	0.81	2.31	1.39	2.22	2.40	1.43	0.15
246	C	1.00	1.09	1.04	1.07	1.55	1.38	1.43	1.37	0.80	0.59	0.60	0.62	1.89	1.26	2.03	2.23	1.50	0.18
247	A	1.00	1.07	1.03	1.00	0.73	0.38	0.47	0.52	1.57	0.47	0.57	0.78	0.82	0.45	0.80	0.95	2.34	0.37
248	G	1.00	1.11	1.04	1.18	0.86	0.84	0.86	0.88	1.38	1.19	1.64	1.29	4.06	2.65	3.94	4.19	1.10	0.04
249	G	1.00	0.98	1.11	1.01	1.19	1.13	1.23	1.18	0.65	0.67	0.68	0.61	0.51	0.42	0.53	0.53	1.11	0.04
250	T	1.00	0.98	0.97	0.86	0.82	0.77	0.90	0.86	0.25	0.41	0.46	0.37	0.34	0.20	0.37	0.40	0.82	-0.09
251	A	1.00	1.01	1.09	1.14	0.58	0.37	0.52	0.50	1.23	0.47	0.57	0.58	0.60	0.27	0.52	0.69	2.46	0.39
252	T	1.00	0.58	0.00	0.60	0.15	0.38	0.45	0.51	1.42	1.27	1.76	1.45	0.72	0.50	0.58	0.51	0.70	-0.16
253	T	1.00	0.15	0.95	0.55	0.50	0.37	0.47	0.50	0.71	0.60	1.11	0.87	1.54	1.22	1.30	1.25	0.66	-0.18
254	G	1.00	1.85	1.51	1.85	1.39	1.02	1.08	1.18	2.20	1.58	1.21	1.95	5.85	4.15	4.39	3.85	0.74	-0.13
255	G	1.0	58.8	61.7	65.7	210.1	218.6	219.2	227.6	158.6	131.0	182.5	169.8	80.0	56.4	66.7	62.4	0.73	-0.14
256	T	1.00	1.22	1.16	1.07	3.20	3.57	3.71	4.28	0.69	0.80	0.96	1.00	2.05	2.17	1.57	1.51	0.50	-0.30
257	G	1.00	1.07	0.98	1.19	0.54	0.57	0.63	0.58	1.28	0.89	1.23	1.09	1.05	0.97	0.91	0.93	1.03	0.01
258	A	1.00	0.95	0.94	0.94	1.28	1.30	1.34	1.27	0.42	0.52	0.48	0.47	0.67	0.56	0.68	0.67	0.87	-0.06
259	G	1.00	1.06	1.07	1.00	1.68	1.72	1.39	1.60	1.50	1.60	1.63	1.52	0.85	0.73	0.83	0.84	0.98	-0.01
260	T	1.00	0.95	0.93	0.91	2.44	2.66	2.94	2.34	0.68	0.76	0.70	0.71	0.84	0.70	0.80	0.83	0.94	-0.03
261	C	1.00	1.10	1.06	1.02	0.58	0.47	0.64	0.65	0.93	0.74	0.72	0.77	1.06	1.01	1.11	1.22	1.38	0.14
262	G	1.00	1.03	0.99	1.17	0.68	0.53	0.62	0.69	0.68	0.73	0.64	0.72	1.55	1.41	1.36	1.22	0.75	-0.13
263	G	1.00	1.07	0.96	1.04	0.68	0.53	0.73	0.70	0.58	0.80	0.80	0.89	1.21	1.22	1.15	0.89	0.49	-0.31
264	A	1.00	0.97	1.06	0.81	0.82	0.65	0.81	0.84	0.45	0.54	0.50	0.50	0.68	0.51	0.69	0.69	0.93	-0.03
265	T	1.00	1.08	1.10	0.91	0.51	0.46	0.55	0.54	0.60	0.73	0.74	0.78	0.97	0.75	0.96	0.93	0.74	-0.13
266	C	1.00	1.13	1.09	1.08	0.61	0.49	0.68	0.65	0.73	0.73	0.61	0.74	2.21	1.78	2.08	1.85	0.83	-0.08
267	G	1.00	1.13	1.09	1.17	0.61	0.59	0.78	0.70	1.42	0.95	1.02	1.28	1.00	0.90	0.95	0.95	1.06	0.02
268	C	1.00	1.07	1.07	0.96	1.26	1.13	1.33	1.43	0.74	0.56	0.52	0.65	0.65	0.58	0.66	0.63	1.12	0.05
269	A	1.00	1.05	1.02	1.04	0.62	0.41	0.53	0.51	0.99	0.46	0.58	0.72	0.51	0.26	0.55	0.62	1.65	0.22
270	G	1.00	0.87	0.89	1.02	0.98	0.91	1.06	0.91	2.24	2.57	3.81	4.12	1.47	1.42	1.40	1.21	0.45	-0.35
271	C	1.00	1.03	0.87	0.91	1.79	1.76	2.17	2.58	0.59	0.62	0.71	0.78	0.63	0.46	0.66	0.63	0.76	-0.12
272	T	1.00	0.91	0.97	0.99	0.80	0.61	0.73	0.76	1.06	1.02	1.63	1.56	1.21	1.08	1.17	1.06	0.60	-0.22
273	T	1.00	0.87	0.97	1.03	0.79	0.72	0.89	0.89	0.76	0.93	0.84	1.06	2.25	2.29	2.10	1.93	0.61	-0.21
274	G	1.00	0.96	0.90	1.13	0.71	0.68	0.71	0.76	0.95	1.13	1.21	1.24	0.85	0.73	0.84	0.77	0.69	-0.16
275	G	1.00	1.00	1.09	1.11	2.44	2.41	2.59	2.81	1.49	2.10	1.96	2.09	1.34	1.16	1.05	0.99	0.53	-0.28
276	A	1.00	0.86	0.84	0.82	2.77	2.79	2.92	3.35	0.66	0.85	0.85	0.96	1.26	0.94	0.89	0.82	0.45	-0.34
277	T	1.00	1.02	1.05	1.09	0.83	0.85	0.91	0.93	1.12	1.48	1.79	2.03	1.00	2.27	1.73	1.47	0.82	-0.09
278	G	1.00	1.01	0.96	1.00	0.70	0.52	0.64	0.58	0.81	0.90	0.81	0.79	1.65	2.18	1.65	1.28	0.80	-0.10
279	G	1.00	1.06	1.04	1.04	1.10	1.14	1.21	1.07	0.92	1.02	0.85	0.98	1.67	0.91	1.26	1.44	0.80	-0.10
280	C	1.00	1.06	1.03	1.08	1.50	1.58	1.61	1.42	0.97	0.94	0.72	0.89	1.06	0.73	0.87	0.78	0.80	-0.09
281	C	1.00	1.07	0.95	1.12	0.81	1.08	1.22	1.00	1.11	0.96	0.86	0.95	1.56	2.95	2.24	1.76	1.32	0.12
282	A	1.00	0.99	0.98	0.96	0.76	0.80	0.86	0.75	1.04	0.69	0.70	0.79	0.82	0.83	0.76	0.68	1.09	0.04
283	C	1.00	0.90	0.84	1.04	0.70	0.59	0.75	0.70	0.72	0.85	0.82	0.94	1.18	1.68	1.18	1.02	0.65	-0.18
284	T	1.00	0.93	0.95	0.97	0.66	0.59	0.67	0.60	1.18	1.65	1.47	1.42	0.91	1.71	0.95	0.79	0.72	-0.14
285	T	1.00	0.92	0.93	1.11	0.87	0.68	0.79	0.78	1.50	2.25	1.61	1.73	2.40	5.74	2.48	2.02	0.73	-0.14
286	A	1.00	0.89	0.95	1.28	0.88	0.68	0.72	0.71	1.59	1.42	3.45	1.41	0.62	1.26	0.77	0.73	1.32	0.12
287	C	1.00	0.89	0.94	0.91	0.75	0.56	0.81	0.72	4.76	6.86	1.69	5.18	1.10	2.60	1.29	1.12	0.94	-0.03
288	C	1.00	0.89	0.93	1.12	0.74	0.70	0.68	0.63	2.69	3.85	3.17	2.56	1.58	3.40	1.81	1.44	0.96	-0.02
289	T	1.00	1.03	1.25	1.24	0.80	0.96	0.86	0.92	1.28	1.96	1.84	1.54	2.09	3.79	2.18	1.71	0.68	-0.17
290	G	1.00	0.96	0.91	1.10	0.64	1.03	0.81	0.75	0.99	1.37	1.27	1.15	0.61	1.11	1.02	0.81	1.14	0.06
291	A	1.00	0.81	0.82	0.87	9.53	11.77	7.76	8.32	0.82	1.02	1.08	0.96	0.94	1.43	1.18	0.90	0.82	-0.09
292	A	1.00	0.73	0.84	0.83	0.48	0.48	0.42	0.47	2.00	2.58	2.49	2.53	0.55	0.75	0.65	0.58	0.83	-0.08
293	T	1.00	0.49	0.71	0.52	1.36	1.38	1.38	1.36	1.00	1.52	1.56	1.04	0.81	0.63	0.52	0.70	0.84	-0.08

294	G	1.00	0.25	0.69	0.08	2.48	1.95	-1.60	2.38	1.42	2.03	1.51	0.82	-1.70	-1.75	0.22	0.36	-	
295	A	1.00	0.50	0.65	0.50	1.65	1.60	0.35	1.70	1.00	1.44	1.44	0.92	0.25	0.16	0.64	0.72	3.06	0.49
296	C	1.00	1.13	1.80	1.74	0.18	-0.33	1.21	-0.45	0.93	0.37	-1.07	0.60	1.87	1.39	1.07	1.59	1.32	0.12
297	C	1.00	0.91	1.07	0.93	0.55	0.58	0.13	0.56	1.18	0.18	0.48	0.62	0.40	0.35	0.95	0.76	3.62	0.56
298	A	1.00	1.09	1.63	1.00	0.82	0.62	0.10	0.45	0.94	0.82	0.83	0.62	0.34	0.60	0.70	0.76	3.36	0.53
299	C	1.00	0.95	0.96	0.95	0.48	0.59	0.58	0.77	1.16	0.92	1.10	1.01	1.98	1.19	1.11	1.30	0.76	-0.12
300	C	1.00	0.62	0.89	0.98	0.67	0.51	0.81	0.81	0.74	0.55	0.61	0.60	1.28	0.92	1.23	1.46	1.40	0.15
301	T	1.00	1.14	1.92	1.46	0.75	0.73	1.43	0.75	1.12	0.90	0.89	0.84	1.03	0.81	0.78	0.58	0.74	-0.13
302	A	1.00	1.26	1.25	0.89	0.78	0.66	0.24	0.78	1.74	1.06	0.89	1.03	0.71	0.45	0.78	1.65	3.95	0.60
303	G	1.00	1.13	0.82	1.33	1.75	1.30	1.05	1.27	0.75	1.23	1.71	1.41	0.77	0.78	1.07	0.97	0.67	-0.17
304	A	1.00	1.37	1.40	2.03	1.56	2.43	1.80	1.75	0.81	1.53	2.38	2.50	1.31	2.44	2.29	0.46	0.11	-0.95
305	G	1.00	3.55	2.39	4.25	3.17	1.81	2.29	0.77	2.44	1.23	2.31	2.96	1.16	2.72	3.00	2.15	1.53	0.18
306	C	1.00	0.95	0.89	1.00	0.39	0.59	0.79	0.78	0.50	0.46	0.34	0.51	0.53	0.54	0.66	0.30	0.56	-0.25
307	C	1.00	0.80	1.04	0.45	0.57	0.50	0.56	0.48	0.86	0.67	0.48	0.48	0.53	0.47	0.56	0.72	2.39	0.38
308	T	1.00	0.58	1.17	1.02	0.89	0.69	0.35	0.74	0.74	1.01	1.18	0.66	0.86	0.51	0.88	1.36	1.77	0.25
309	T	1.00	0.88	0.95	1.53	0.87	0.82	0.55	0.76	0.60	0.63	1.33	0.99	0.62	0.49	0.63	0.82	0.79	-0.10
310	G	1.00	1.18	0.81	0.89	0.65	0.66	0.61	0.60	1.01	0.62	0.72	0.81	0.54	0.30	0.72	0.54	1.24	0.09
311	G	1.00	1.46	1.76	1.30	1.43	1.40	2.93	1.84	1.14	1.36	1.28	1.49	0.96	1.42	1.36	0.96	0.77	-0.12
312	A	1.00	2.34	2.51	2.29	6.08	5.94	1.72	6.11	2.88	3.27	2.36	3.48	3.29	3.32	2.67	2.48	0.62	-0.20
313	T	1.00	0.74	1.03	1.11	0.82	1.09	0.66	0.71	1.53	1.11	1.29	1.72	1.13	0.78	1.38	1.55	1.21	0.08
314	C	1.00	1.23	1.73	1.19	0.85	1.07	0.95	0.95	1.80	1.24	1.30	1.11	1.94	1.89	2.47	1.30	1.09	0.04
315	C	1.00	0.56	1.31	0.70	0.60	0.42	0.49	0.47	0.73	0.88	0.57	0.40	0.80	0.39	1.19	1.42	3.26	0.51
316	A	1.00	1.19	1.03	1.11	0.59	0.41	0.26	0.52	1.06	0.65	0.73	0.34	0.35	0.53	0.34	0.87	7.92	0.90
317	G	1.00	1.26	1.46	1.10	1.21	1.03	0.90	0.86	1.66	0.94	1.82	2.61	1.07	1.61	1.37	1.11	0.66	-0.18
318	G	1.00	1.34	1.72	2.27	2.30	2.75	2.38	2.53	2.79	1.86	1.79	2.22	3.90	1.92	3.19	1.83	0.59	-0.23
319	A	1.00	1.06	1.33	1.34	1.59	1.53	0.99	1.78	1.42	2.03	1.46	1.84	1.11	0.85	1.55	1.29	0.90	-0.05
320	G	1.00	1.01	1.17	1.24	1.13	0.72	1.95	0.70	1.00	1.23	1.54	0.94	0.83	0.77	0.78	1.07	1.38	0.14
321	A	1.00	1.18	1.08	1.40	2.55	2.58	0.58	2.47	0.33	0.78	1.03	1.01	1.33	0.82	1.08	0.88	0.22	-0.66
322	A	1.00	1.24	1.69	1.80	1.56	1.69	0.45	0.99	0.88	0.78	1.64	1.40	1.17	0.72	1.42	0.67	0.36	-0.44
323	C	1.00	0.93	1.50	1.22	0.58	0.47	0.31	0.58	0.32	0.47	1.51	1.25	0.76	1.01	0.97	0.98	0.33	-0.48
324	G	1.00	2.50	2.04	2.11	0.31	0.66	0.85	0.56	1.53	1.57	1.07	1.49	0.79	1.46	1.43	1.07	1.39	0.14
325	G	1.00	0.56	0.51	0.85	1.04	1.14	0.85	0.91	1.65	1.35	1.24	1.38	1.00	1.03	1.30	1.31	1.57	0.20
326	C	1.00	0.83	0.96	0.85	1.21	1.16	0.89	1.21	0.60	0.73	0.98	0.79	0.62	0.67	0.66	0.64	0.79	-0.10
327	G	1.00	1.11	1.68	1.47	1.00	0.94	0.88	1.21	1.00	0.85	1.10	0.94	0.76	0.92	0.94	0.88	1.24	0.09
328	G	1.00	0.81	1.24	0.99	1.09	1.22	0.90	1.21	0.75	1.50	1.18	1.07	0.95	1.10	1.23	0.98	0.72	-0.14
329	C	1.00	0.89	0.87	1.28	1.08	1.32	0.80	1.21	0.93	0.89	1.05	0.96	1.25	1.03	1.56	1.70	1.32	0.12
330	T	1.00	1.73	2.46	2.27	2.48	2.77	1.51	2.49	1.52	2.01	2.47	2.61	6.26	3.12	5.78	6.33	0.59	-0.23
331	G	1.00	0.97	0.78	0.93	0.98	1.34	0.68	1.00	1.03	0.92	1.06	0.91	0.60	0.64	1.32	1.63	3.06	0.49
332	G	1.00	1.42	1.67	1.34	1.72	2.22	1.35	1.75	1.84	1.77	1.42	1.34	0.85	1.19	0.87	0.64	1.03	0.01
333	g	1.00	0.64	1.20	0.88	1.02	1.30	1.02	0.94	0.58	1.05	1.25	0.91	0.54	0.55	0.72	0.33	0.39	-0.40
334	t	1.00	1.07	1.25	1.45	1.04	1.13	0.75	0.99	1.22	0.98	1.00	0.82	0.35	0.36	0.61	0.80	3.33	0.52
335	a	1.00	1.47	2.12	1.91	2.48	3.35	0.93	2.86	2.26	1.93	1.14	2.08	0.96	0.65	0.79	1.25	1.42	0.15
336	a	1.00	1.02	0.84	0.98	1.71	2.30	1.05	1.76	3.34	3.24	1.87	2.70	0.89	0.74	0.65	0.73	1.01	0.01
337	g	1.00	1.78	2.21	2.20	5.34	5.18	7.59	3.42	8.63	14.88	13.28	11.59	1.44	1.59	1.67	1.04	0.54	-0.27
338	a	1.00	1.11	1.26	1.35	14.06	12.91	5.70	13.07	1.41	2.11	4.04	2.25	1.19	1.47	1.00	0.71	0.38	-0.43
339	a	1.00	0.82	0.96	1.13	1.04	1.10	0.59	0.62	0.99	0.36	1.54	1.27	0.61	0.88	0.84	0.76	0.98	-0.01
340	c	1.00	1.56	1.58	1.54	0.31	0.62	0.28	0.36	1.60	1.51	1.75	1.92	1.24	1.23	1.29	1.45	0.98	-0.01
341	c	1.00	0.89	1.47	1.23	0.45	0.25	0.94	0.60	1.22	0.96	0.98	0.86	1.17	0.78	1.02	1.46	1.75	0.24
342	a	1.00	1.07	1.20	1.00	0.56	0.55	0.33	0.59	0.96	0.67	0.57	0.76	0.54	0.49	0.81	0.78	1.82	0.26
343	a	1.00	1.48	1.56	1.51	1.14	1.34	0.78	1.29	1.46	1.47	1.36	1.03	1.43	1.13	1.10	1.08	1.07	0.03
344	g	1.00	1.59	2.01	2.16	2.29	2.70	2.15	1.50	1.30	1.30	1.58	1.68	1.19	1.11	1.17	0.92	0.60	-0.22
345	c	1.00	1.80	2.43	2.33	0.95	0.84	0.89	1.48	1.29	0.92	1.35	1.10	0.95	0.77	0.99	1.07	1.32	0.12
346	c	1.00	1.22	1.28	2.24	0.85	0.59	0.79	0.65	0.81	0.83	1.23	0.78	0.82	0.57	0.75	0.95	1.22	0.09
347	c	1.00	0.14	0.60	0.37	0.45	0.47	0.54	0.56	0.57	0.82	1.03	0.72	0.62	0.49	0.72	0.86	1.11	0.04
348	c	1.00	1.32	1.29	0.91	0.62	0.57	0.42	0.61	0.63	0.78	0.58	0.79	0.59	0.50	0.82	0.82	1.11	0.04
349	t	1.00	1.05	1.49	1.67	0.96	0.94	0.64	0.79	0.89	1.09	0.95	0.98	0.61	0.50	0.94	1.03	1.54	0.19
350	t	1.00	1.32	1.37	1.66	1.13	1.26	1.06	1.16	0.89	0.90	1.01	1.21	1.29	0.76	1.77	1.35	0.77	-0.11
351	g	1.00	1.28	1.28	1.36	1.60	1.65	2.15	1.20	1.40	1.79	1.41	1.81	0.65	0.97	0.89	0.75	0.88	-0.05
352	t	1.00	1.05	1.25	1.28	2.77	3.08	1.23	3.41	1.18	1.08	1.56	1.13	1.34	1.07	1.03	1.03	0.81	-0.09
353	g	1.00	1.05	1.20	1.08	0.97	0.95	2.20	0.83	1.25	1.47	1.07	1.27	0.84	0.73	0.82	0.80	0.93	-0.03
354	t	1.00	1.04	1.32	1.17	2.27	2.20	0.63	1.94	0.83	0.94	0.96	0.81	0.76	0.54	0.88	0.90	1.21	0.08
355	g	1.00	1.30	1.94	1.47	1.25	0.85	1.58	0.88	1.49	1.46	1.91	1.59	0.85	0.77	0.99	1.19	1.32	0.12
356	t	1.00	1.12	1.30	1.41	1.49	1.54	0.65	1.51	0.86	0.89	0.81	0.87	0.59	0.39	0.67	0.74	1.24	0.09
357	c	1.00	1.06	1.24	1.18	0.70	0.83	0.94	0.71	1.32	1.17	1.37	1.06	0.84	0.81	0.93	1.15	1.71	0.23
358	c	1.00	1.20	1.45	1.38	0.80	0.60	0.71	0.70	1.22	1.16	1.23	1.07	0.82	0.67	0.92	1.08	1.49	0.17
359	c	1.00	1.08	1.55	1.30	0.81	0.67	0.36	0.69	1.10	1.06	0.92	0.95	0.74	0.47	0.89	1.01	1.57	0.20
360	t	1.00	1.08	1.19	1.28	0.58	0.44	0.42	0.48	0.51	0.66	0.73	0.69	0.46	0.26	0.61	0.59	0.97	-0.01
361	t	1.00	1.42	1.07	1.11	0.64	0.61	0.63	0.61	1.25	1.42	1.26	1.36	0.98	0.64	1.17	0.95	0.89	-0.05
362	t	1.00	1.30	1.62	1.37	0.96	1.00	1.09	0.90	3.47	3.60	4.03	3.95	1.37	1.22	1.53	1.49	0.95	-0.02
363	t	1.00	1.29	1.39	1.14	0.85	0.91	1.03	0.93	2.45	3.00	3.14	2.81	1.07	1.08	1.02	1.06	0.86	-0.07
364	c	1.00	1.09	1.30	1.26	0.83	0.72	0.81	0.73	1.58	1.73	1.98	1.72	0.98	0.87	1.07	1.07	1.00	0.00
365	t	1.00	1.06	1.30	1.24	0.91	0.81	0.83	0.85	1.83	1.92	2.16	1.98	1.21	1.18	1.23	1.42	1.09	0.04
366	t	1.00	1.13	1.29	1.28	0.96	0.89	0.84	0.95	1.21	1.35	1.44	1.43	3.12	2.60	3.21	3.11	0.84	-0.07
367	t	1.00	0.94	0.92	0.99	0.75	0.71	0.81	0.84	0.81	0.75	0.79	0.87	0.75	0.76	0.95	1.00	1.24	0.09
368	g	1.00	1.06	0.92	0.97	0.65	0.80	0.95	0.78	0.67	0.73	0.78	0.74	1.00	0.84	1.07	0.95	0.86	-0.07
369	g	1.00	1.08	1.05	1.13	1.07	0.94	1.05	1.13	1.12	0.95	1.03	0.94	0.92	1.14	1.11	1.17	1.51	0.18

370	c	1.00	1.02	0.89	0.86	0.90	0.92	0.65	0.97	0.68	0.81	0.64	0.74	0.64	0.67	0.76	0.69	0.99	0.00
371	c	1.00	1.29	1.12	1.23	0.90	0.98	0.85	0.90	0.91	1.07	1.04	0.99	1.21	1.01	1.16	1.05	0.80	-0.10
372	t	1.00	1.09	1.05	1.07	0.64	0.65	0.69	0.68	0.67	0.74	0.65	0.71	0.60	0.51	0.76	0.72	1.13	0.05
373	c	1.00	1.17	1.26	1.17	0.86	0.86	0.75	0.87	1.43	1.55	1.85	2.15	0.81	0.89	0.90	1.08	0.89	-0.05
374	t	1.00	1.05	1.26	1.24	0.97	1.02	0.76	1.00	1.55	1.52	1.39	1.27	0.94	1.08	0.88	1.01	1.31	0.12
375	g	1.00	1.09	1.06	1.01	0.59	0.58	0.63	0.62	0.59	0.56	0.61	0.56	0.50	0.40	0.65	0.66	1.42	0.15
376	g	1.00	1.26	1.53	1.38	1.28	1.48	0.90	1.33	1.25	1.27	1.11	1.05	0.98	1.37	0.99	1.22	1.48	0.17
377	t	1.00	1.21	1.30	1.50	1.55	1.50	1.09	1.50	0.94	1.18	1.21	1.14	1.32	1.35	1.19	1.27	0.79	-0.10
378	c	1.00	1.13	1.28	1.29	1.07	1.05	0.81	1.08	0.98	1.02	1.01	0.99	1.41	1.10	1.33	1.45	1.02	0.01
379	a	1.00	1.07	1.64	1.30	0.80	0.62	0.52	0.72	1.62	0.85	0.92	0.85	1.04	0.84	1.05	1.22	2.22	0.35
380	g	1.00	1.10	1.34	1.37	0.91	0.95	0.67	0.97	1.00	1.31	1.43	1.30	2.03	1.79	2.28	2.27	0.85	-0.07
381	a	1.00	1.15	1.20	1.25	1.65	1.86	1.28	1.90	0.72	0.98	0.97	0.90	0.86	0.76	1.15	1.07	1.00	0.00
382	g	1.00	1.07	1.21	1.12	0.90	0.96	0.70	0.91	1.02	1.11	1.10	1.18	0.68	0.60	0.79	0.88	1.13	0.05
383	a	1.00	1.04	1.23	1.20	1.29	1.38	1.07	1.45	0.85	1.00	0.90	0.91	0.83	0.74	0.77	0.84	0.94	-0.03
384	t	1.00	1.18	1.26	1.35	0.95	0.93	0.80	0.92	0.91	0.95	1.01	1.02	0.79	0.78	0.88	0.95	1.07	0.03
385	c	1.00	1.05	1.20	1.13	0.81	0.80	0.79	0.81	0.80	0.79	0.93	0.85	0.95	1.07	1.08	1.19	1.17	0.07
386	c	1.00	1.03	1.23	1.15	0.85	0.86	0.78	0.90	1.10	0.87	0.83	0.91	1.21	1.11	1.23	1.39	1.38	0.14
387	c	1.00	1.07	1.23	1.20	0.88	0.91	0.74	0.91	0.91	0.87	0.77	0.81	2.10	1.47	2.17	2.23	1.19	0.08
388	c	1.00	1.07	1.12	1.13	0.84	0.93	0.80	0.94	0.91	0.86	0.82	0.79	2.23	1.43	2.33	2.21	1.14	0.06
389	a	1.00	1.09	1.15	0.94	0.71	0.70	0.59	0.80	1.35	0.63	0.67	0.66	0.58	0.46	0.63	0.61	2.16	0.33
390	a	1.00	1.11	1.05	1.13	0.78	0.83	0.89	0.94	0.65	0.94	0.91	0.91	1.03	1.14	1.24	1.07	0.74	-0.13
391	c	1.00	1.11	0.91	0.99	0.71	0.79	0.93	0.81	0.80	0.89	0.83	0.80	1.66	1.40	1.82	1.44	0.87	-0.06
392	a	1.00	1.01	1.04	0.89	0.62	0.57	0.50	0.69	1.10	0.59	0.59	0.59	0.48	0.39	0.54	0.59	2.28	0.36
393	g	1.00	1.30	1.12	1.30	0.88	1.16	1.13	1.19	1.08	1.69	1.57	1.56	0.81	1.00	0.89	0.93	0.79	-0.10
394	c	1.00	1.18	1.09	1.18	1.96	2.20	2.10	2.37	0.86	1.15	1.07	1.02	0.68	0.73	0.75	0.90	1.12	0.05
395	c	1.00	1.09	1.20	1.15	0.94	1.02	0.83	0.93	0.93	0.96	1.09	0.98	0.70	0.38	0.73	0.86	1.17	0.07
396	c	1.00	1.10	1.07	1.05	0.81	0.92	0.76	0.87	1.00	0.90	0.90	0.88	0.81	0.62	0.84	0.89	1.25	0.10
397	t	1.00	1.11	1.06	1.10	0.83	0.92	0.78	0.87	0.84	0.92	0.95	0.90	0.75	0.46	0.77	0.85	1.06	0.03
398	t	1.00	1.08	0.89	0.97	0.75	0.85	0.82	0.81	0.75	0.85	0.88	0.81	0.95	0.74	0.97	0.99	0.95	-0.02
399	c	1.00	1.06	0.92	0.93	0.78	0.88	0.86	0.80	0.83	0.86	0.86	0.84	1.27	0.94	1.20	1.13	0.86	-0.06
400	t	1.00	1.06	0.99	1.04	0.83	0.94	0.81	0.93	0.98	1.00	0.96	0.99	0.79	0.64	0.85	0.87	1.10	0.04
401	t	1.00	1.04	0.89	0.91	0.79	0.84	0.82	0.82	0.79	0.87	0.94	0.93	0.84	0.80	0.90	0.87	0.88	-0.05
402	c	1.00	1.19	0.96	1.06	0.80	0.89	0.89	0.89	1.17	1.03	1.00	1.02	1.48	1.62	1.39	1.36	1.05	0.02
403	t	1.00	1.09	0.99	0.99	0.77	0.82	0.72	0.83	0.94	0.86	0.92	0.91	1.20	1.20	1.13	1.14	0.99	0.00
404	g	1.00	1.08	0.94	0.88	0.85	1.03	1.06	0.99	1.18	1.14	1.02	1.04	0.82	0.90	0.81	0.89	1.23	0.09
405	t	1.00	1.11	1.12	1.10	1.99	2.07	1.97	1.83	1.16	1.00	0.95	0.97	0.83	0.73	0.77	0.93	1.33	0.12
406	a	1.00	1.02	0.96	0.81	0.68	0.60	0.61	0.74	1.55	0.89	0.82	0.86	0.64	0.45	0.60	0.90	2.51	0.40
407	t	1.00	1.12	1.08	1.14	0.79	0.88	0.80	0.89	1.93	2.32	2.25	2.26	1.06	1.17	1.08	1.16	0.94	-0.03
408	c	1.00	1.11	1.01	0.91	0.73	0.79	0.78	0.88	1.04	1.04	1.06	1.03	1.96	2.42	1.96	2.02	1.04	0.02
409	t	1.00	0.99	0.97	0.93	0.74	0.76	0.58	0.69	0.74	0.76	0.79	0.84	0.72	0.58	0.69	0.80	0.99	0.00
410	c	1.00	0.95	0.93	0.89	0.67	0.65	0.70	0.72	0.88	0.82	0.84	0.83	2.21	2.63	1.94	1.98	0.94	-0.02
411	t	1.00	0.90	0.89	0.90	0.69	0.63	0.75	0.69	0.82	0.80	0.79	0.75	2.51	2.85	2.13	2.00	0.86	-0.06
412	t	1.00	0.91	0.72	0.80	0.65	0.63	0.66	0.67	0.73	0.79	0.76	0.83	1.24	1.60	1.19	1.14	0.81	-0.09
413	t	1.00	0.94	0.87	0.92	0.73	0.75	0.77	0.80	0.96	0.97	1.03	1.06	0.92	1.18	0.86	0.93	0.91	-0.04
414	c	1.00	1.01	0.85	0.82	0.75	0.69	0.79	0.74	1.12	0.99	1.01	1.09	1.28	1.78	1.50	1.47	1.18	0.07
415	t	1.00	0.93	0.80	0.81	0.77	0.88	0.76	0.85	1.02	0.86	0.77	0.98	2.00	2.88	1.73	1.45	0.75	-0.12
416	g	1.00	0.87	0.50	0.61	0.77	0.87	1.14	0.89	1.02	1.09	0.98	1.13	0.78	1.15	0.71	0.65	0.76	-0.12
417	t	1.00	0.99	0.91	0.86	6.47	6.59	6.03	6.29	1.82	1.95	1.90	1.99	1.62	2.03	1.39	1.22	0.68	-0.16
418	t	1.00	0.88	0.89	0.82	0.65	0.60	0.68	0.66	1.70	1.69	1.64	1.83	1.21	1.51	1.05	1.01	0.78	-0.11
419	g	1.00	0.81	0.72	0.64	0.63	0.71	1.30	0.78	2.22	2.54	2.10	2.25	1.18	1.62	1.13	1.08	0.90	-0.04
420	t	1.00	0.85	0.85	0.95	3.47	3.66	4.59	3.72	0.82	0.83	0.78	0.86	0.96	1.15	0.87	0.86	0.85	-0.07
421	g	1.00	0.79	0.71	0.78	0.92	0.88	1.50	0.92	1.12	1.28	1.19	1.17	0.64	0.71	0.62	0.60	0.91	-0.04
422	t	1.00	0.83	0.96	0.92	3.50	3.15	4.33	3.30	0.92	0.93	0.88	0.96	0.96	1.20	1.02	0.88	0.88	-0.06
423	t	1.00	0.89	0.69	0.86	0.56	0.49	0.62	0.53	1.10	1.19	1.11	1.21	1.05	1.23	0.96	0.92	0.80	-0.10
424	g	1.00	0.84	0.51	0.63	0.43	0.42	0.84	0.47	0.66	0.73	0.65	0.72	0.79	1.00	0.72	0.67	0.76	-0.12
425	g	1.00	0.80	0.66	0.76	1.03	0.99	1.65	0.97	0.75	0.76	0.68	0.71	0.78	0.99	0.72	0.65	0.88	-0.05
426	g	1.00	0.86	0.85	1.00	2.70	2.63	3.11	2.51	1.81	1.91	1.59	1.71	1.64	2.16	1.50	1.41	0.90	-0.04
427	t	1.00	0.90	0.87	1.03	1.03	0.91	1.03	0.89	0.49	0.58	0.59	0.58	1.08	1.22	0.96	0.86	0.67	-0.17
428	g	1.00	0.80	0.51	0.59	0.25	0.22	0.49	0.30	0.40	0.37	0.33	0.34	0.76	0.86	0.75	0.63	0.99	-0.01
429	a	1.00	0.82	0.58	0.72	2.03	1.85	2.10	1.80	0.58	0.54	0.62	0.66	1.00	1.08	0.91	0.81	0.70	-0.16
430	t	1.00	0.85	0.76	0.76	0.63	0.51	0.68	0.62	1.52	1.63	1.69	2.06	1.03	1.08	0.89	0.81	0.58	-0.24
431	t	1.00	0.91	0.87	0.87	0.61	0.50	0.44	0.45	0.78	0.71	0.74	0.89	1.15	1.12	1.04	1.00	0.76	-0.12
432	g	1.00	1.07	1.03	0.94	1.55	1.39	1.17	1.38	1.36	1.22	1.27	1.22	0.53	0.93	0.67	0.64	1.34	0.13
433	t	1.00	1.01	1.01	1.14	4.11	4.63	3.64	3.41	0.93	0.80	0.76	0.83	0.74	1.32	0.60	0.57	0.87	-0.06
434	t	1.00	0.85	0.86	0.94	0.70	0.55	0.61	0.49	1.34	1.06	1.28	1.23	0.94	1.30	0.84	0.98	1.13	0.05
435	g	1.00	1.05	1.16	0.95	0.68	0.42	0.43	0.68	1.02	0.82	0.84	0.81	1.14	1.62	1.09	1.11	1.22	0.09
436	g	1.00	0.78	1.07	0.91	1.09	1.16	1.12	0.96	0.62	0.47	0.64	0.53	1.15	1.17	1.01	0.95	0.96	-0.02
437	a	1.00	0.98	0.93	1.08	0.89	0.81	0.85	0.72	0.59	0.57	0.60	0.58	0.71	0.76	0.72	0.76	1.09	0.04
438	g	1.00	1.02	0.99	0.95	0.69	0.57	0.63	0.61	0.81	0.74	0.78	0.77	0.92	1.09	0.92	0.85	0.97	-0.01
439	a	1.00	0.96	0.84	0.94	0.91	0.91	0.88	0.82	0.47	0.46	0.43	0.47	0.52	0.59	0.57	0.64	1.22	0.09
440	c	1.00	0.96	0.98	0.95	0.54	0.56	0.57	0.52	0.66	0.65	0.67	0.66	0.61	0.66	0.62	0.66	1.09	0.04
441	g	1.00	0.87	0.90	0.92	0.61	0.58	0.50	0.56	1.06	0.73	0.81	0.82	1.12	1.18	1.10	1.03	1.19	0.08
442	t	1.00	1.07	0.92	0.92	2.15	2.28	1.95	1.89	0.71	0.60	0.63	0.56	1.22	1.27	1.08	1.16	1.22	0.09
443	t	1.00	0.97	1.01	0.94	0.75	0.75	0.87	0.86	1.22	1.08	1.18	1.26	1.60	1.61	1.26	1.03	0.62	-0.20
444	g	1.00	0.93	1.09	0.95	1.02	1.12	1.10	1.11	1.30	1.37	1.25	1.31	0.74	0.82	0.73	0.70	0.93	-0.03
445	a	1.00	0.92	1.02	1.10	8.77	9.08	7.93	8.53	1.49	1.46	1.24	1.36	0.71	1.26	0.65	0.73	1.14	0.06

446	t	1.00	0.90	1.01	1.06	0.64	0.65	0.59	0.62	2.75	3.07	2.73	2.89	0.61	0.89	0.58	0.53	0.84	-0.08
447	a	1.00	1.23	1.34	1.29	0.66	0.43	0.58	0.62	1.65	0.60	0.89	0.84	0.66	0.59	0.68	0.91	2.71	0.43
448	g	1.00	0.86	0.83	0.66	0.74	0.77	0.69	0.83	1.89	2.09	2.14	2.14	0.79	1.32	0.60	0.70	0.78	-0.11
449	t	1.00	0.88	0.94	0.92	3.64	3.56	3.50	3.52	0.80	0.87	0.91	0.89	1.19	1.69	1.08	1.02	0.77	-0.11
450	t	1.00	0.91	0.90	1.07	0.66	0.60	0.59	0.62	0.82	0.61	0.80	0.68	1.37	1.58	1.25	1.25	1.10	0.04
451	g	1.00	0.98	1.11	1.13	0.72	0.55	0.64	0.59	0.95	0.80	0.88	0.85	1.13	1.27	1.10	1.03	1.01	0.00
452	a	1.00	0.88	0.79	0.96	1.19	1.11	1.13	1.24	0.77	0.65	0.88	0.62	0.91	1.44	1.01	0.95	1.29	0.11
453	g	1.00	0.97	0.95	0.96	0.57	0.51	0.62	0.59	1.27	0.93	1.13	1.30	1.45	1.38	1.18	1.20	0.81	-0.09
454	g	1.00	0.83	0.96	0.76	1.19	1.18	1.14	1.19	1.03	0.98	1.13	1.12	0.79	1.11	0.79	0.84	0.98	-0.01
455	a	1.00	1.06	1.15	1.06	1.35	1.42	1.38	1.55	0.63	0.77	0.68	0.70	0.67	0.70	0.64	0.70	0.93	-0.03
456	a	1.00	0.86	0.95	0.90	0.73	0.69	0.72	0.80	1.01	0.96	1.01	1.06	0.62	0.83	0.73	0.75	1.15	0.06
457	a	1.00	0.88	1.03	0.88	0.52	0.56	0.67	0.66	1.55	1.51	1.71	1.60	0.74	1.06	0.73	0.72	0.95	-0.02
458	c	1.00	0.88	1.08	0.95	0.61	0.59	0.65	0.63	1.47	1.60	1.62	1.64	0.86	1.11	0.79	0.87	0.91	-0.04
459	c	1.00	0.95	0.89	0.89	0.66	0.57	0.61	0.61	1.25	0.77	0.88	0.89	1.36	1.25	1.24	1.21	1.25	0.10
460	t	1.00	1.02	1.05	0.96	0.69	0.70	0.83	0.76	0.67	0.74	0.79	0.64	1.98	1.47	1.69	1.63	0.87	-0.06
461	g	1.00	0.98	1.14	0.89	0.78	0.98	0.92	1.06	0.95	1.14	1.00	0.99	2.97	1.96	2.56	2.29	0.74	-0.13
462	a	1.00	0.94	0.89	0.82	4.47	4.86	4.75	4.63	0.94	0.96	1.03	1.01	0.58	1.19	0.77	0.87	1.39	0.14
463	c	1.00	0.92	0.97	0.86	0.52	0.55	0.62	0.58	1.56	1.66	1.78	1.77	0.82	1.04	0.82	0.93	1.00	0.00
464	t	1.00	0.86	0.94	0.97	0.56	0.54	0.62	0.64	0.87	0.76	0.87	0.82	2.17	1.56	2.14	2.12	1.05	0.02
465	g	1.00	1.02	1.14	0.94	0.59	0.61	0.66	0.72	0.76	0.76	0.69	0.70	0.99	0.97	1.01	0.94	1.03	0.01
466	g	1.00	1.02	1.08	1.07	0.82	0.90	0.90	0.87	0.70	0.59	0.76	0.63	0.84	1.07	0.94	1.00	1.32	0.12
467	c	1.00	0.93	1.03	1.11	0.88	1.16	1.05	1.09	0.62	0.68	0.72	0.67	0.80	1.25	1.06	1.15	1.34	0.13
468	c	1.00	0.90	0.98	0.98	0.39	0.42	0.51	0.44	0.53	0.62	0.62	0.56	1.52	1.22	1.50	1.57	0.98	-0.01
469	t	1.00	0.92	1.01	1.10	0.47	0.52	0.58	0.50	0.59	0.66	0.66	0.63	1.22	0.95	1.24	1.26	0.97	-0.01
470	c	1.00	0.96	1.03	0.99	0.60	0.73	0.73	0.69	0.66	0.74	0.64	0.66	1.35	1.06	1.33	1.26	0.94	-0.03
471	a	1.00	0.90	0.98	1.07	0.52	0.55	0.56	0.56	1.05	0.70	0.74	0.70	0.55	0.46	0.57	0.64	1.76	0.24
472	t	1.00	0.94	0.96	1.02	0.54	0.63	0.72	0.70	2.37	3.15	3.06	3.06	0.72	0.85	0.79	0.75	0.80	-0.09
473	t	1.00	1.03	1.02	0.98	0.52	0.67	0.73	0.67	2.02	2.70	2.39	2.36	1.43	1.77	1.32	1.27	0.76	-0.12
474	t	1.00	1.10	0.88	1.09	0.48	0.66	0.61	0.71	1.72	2.15	2.03	1.95	1.00	1.15	0.92	1.02	0.90	-0.05
475	c	1.00	1.10	1.11	1.10	0.54	0.50	0.58	0.63	1.27	1.32	1.32	1.27	1.55	1.55	1.34	1.34	0.87	-0.06
476	a	1.00	0.85	0.94	1.02	0.53	0.48	0.45	0.48	1.39	0.72	0.80	0.84	0.62	0.44	0.60	0.65	1.76	0.25
477	c	1.00	0.87	0.91	0.86	0.51	0.57	0.69	0.60	2.24	3.79	3.28	2.94	1.69	1.69	1.37	1.35	0.61	-0.22
478	c	1.00	0.98	0.85	0.99	0.52	0.80	0.69	0.78	0.97	1.23	1.12	1.01	1.21	1.24	1.08	1.24	0.99	-0.01
479	a	1.00	0.96	0.97	0.90	0.52	0.56	0.54	0.58	1.10	0.52	0.56	0.59	0.62	0.58	0.61	0.68	2.03	0.31
480	c	1.00	0.95	0.83	1.01	0.53	0.54	0.53	0.63	0.79	1.53	1.33	1.09	1.67	1.39	1.41	1.39	0.60	-0.22
481	a	1.00	1.03	1.02	1.00	0.57	0.48	0.50	0.57	1.31	0.63	0.59	0.66	0.58	0.45	0.59	0.66	2.27	0.36
482	a	1.00	0.87	1.06	0.83	0.47	0.72	0.87	0.86	0.87	1.69	1.64	1.52	3.14	3.54	3.20	2.94	0.54	-0.27
483	g	1.00	0.83	0.96	0.90	0.66	1.09	0.92	1.09	1.96	3.43	3.07	3.84	2.43	2.47	2.23	2.04	0.43	-0.37
484	a	1.00	1.14	1.06	1.12	3.02	3.53	3.50	4.28	0.88	1.12	0.91	0.93	2.19	2.26	2.09	2.03	0.88	-0.06
485	g	1.00	1.12	1.13	0.90	0.47	0.69	0.70	0.75	1.19	1.70	1.33	1.41	1.58	1.47	1.64	1.51	0.80	-0.10
486	g	1.00	0.98	0.93	1.01	1.00	1.21	1.08	1.12	0.70	1.16	0.92	0.87	1.20	0.95	1.22	1.23	0.82	-0.09
487	t	1.00	1.11	1.19	1.10	1.16	1.39	1.42	1.52	0.64	0.92	0.79	0.83	0.75	0.49	0.81	0.74	0.77	-0.12
488	t	1.00	1.06	1.03	0.87	0.56	0.71	0.76	0.78	0.80	1.18	0.90	0.92	1.22	1.10	1.24	1.12	0.80	-0.10
489	a	1.00	0.96	1.16	0.89	0.58	0.61	0.68	0.66	1.42	1.15	1.16	1.09	0.92	0.73	0.89	1.02	1.43	0.16
490	a	1.00	0.98	0.95	0.73	0.56	0.70	0.70	0.76	2.84	4.20	4.09	4.70	0.78	0.80	0.79	0.75	0.58	-0.23
491	c	1.00	1.27	1.13	0.96	0.58	0.65	0.62	0.75	3.83	6.64	5.27	5.56	1.28	1.25	1.05	0.99	0.53	-0.27
492	t	1.00	1.02	1.02	1.00	0.55	0.58	0.69	0.55	0.93	1.41	1.20	1.14	1.01	0.67	1.00	1.05	0.85	-0.07
493	c	1.00	1.10	1.12	0.97	0.59	0.64	0.68	0.71	0.49	0.73	0.64	0.61	2.40	2.41	2.36	2.29	0.77	-0.12
494	c	1.00	1.03	0.97	0.95	0.57	0.71	0.63	0.66	0.59	0.73	0.64	0.66	1.35	1.15	1.30	1.20	0.79	-0.10
495	t	1.00	1.00	1.00	0.91	0.44	0.48	0.50	0.47	0.40	0.57	0.54	0.54	2.27	2.33	1.92	1.65	0.53	-0.28
496	t	1.00	1.15	1.00	1.02	0.75	0.79	0.78	0.82	0.57	0.82	0.79	0.77	3.87	3.77	3.48	2.89	0.55	-0.26
497	g	1.00	1.15	1.02	1.01	0.91	1.02	1.08	1.30	0.99	1.32	1.01	1.16	1.82	1.56	1.79	1.47	0.69	-0.16
498	g	1.00	1.13	0.97	1.24	1.65	1.79	1.96	1.98	1.16	1.66	1.44	1.85	1.29	1.21	1.28	1.21	0.59	-0.23
499	a	1.00	1.16	0.97	0.97	2.41	3.51	3.53	3.44	0.80	0.94	0.82	0.81	0.99	0.86	0.90	0.83	0.83	-0.08
500	a	1.00	0.99	0.85	0.93	0.95	0.87	1.07	1.11	0.68	0.80	1.23	1.69	1.10	1.23	1.36	1.31	0.48	-0.32
501	a	1.00	1.29	1.10	1.05	0.50	0.63	0.54	0.45	2.61	3.38	2.50	3.27	1.65	1.84	1.03	0.95	0.46	-0.34
502	t	1.00	1.25	1.15	1.13	1.03	0.95	0.92	0.94	2.96	3.92	3.91	3.70	0.96	0.70	0.91	0.89	0.74	-0.13
503	a	1.00	1.12	1.06	1.10	0.86	0.66	0.71	0.73	1.26	0.92	1.02	0.91	0.58	0.37	0.63	0.71	1.71	0.23
504	c	1.00	1.10	1.09	1.00	0.89	0.89	0.87	0.87	3.16	4.15	4.03	3.61	1.43	1.19	1.22	1.19	0.73	-0.14
505	a	1.00	1.06	0.99	0.96	0.89	0.62	0.64	0.70	1.25	0.71	0.81	0.75	0.69	0.50	0.70	0.76	1.84	0.26
506	a	1.00	1.20	1.04	0.89	1.19	1.20	1.12	1.29	2.20	2.94	2.90	2.61	2.58	2.46	2.38	2.13	0.69	-0.16
507	a	1.00	1.05	0.96	0.97	1.04	1.11	0.96	1.17	3.80	4.74	4.72	4.14	0.82	0.75	0.81	0.81	0.90	-0.04
508	t	1.00	1.26	1.17	1.21	2.02	1.95	1.50	1.80	4.32	5.20	5.04	4.47	1.28	1.26	1.30	1.32	1.00	0.00
509	g	1.00	1.22	1.15	0.99	2.38	2.78	2.41	1.97	3.53	3.93	3.69	3.49	0.81	0.71	0.90	0.97	1.21	0.08
510	a	1.00	1.09	1.04	0.96	8.95	7.71	8.61	7.45	1.53	1.49	1.84	1.48	0.74	0.58	0.83	0.90	1.26	0.10
511	c	1.00	1.08	1.01	0.96	1.24	1.34	0.86	1.15	2.72	2.93	2.84	2.57	0.76	0.68	0.75	0.90	1.25	0.10
512	c	1.00	1.12	0.98	1.05	0.72	0.67	0.64	0.75	1.14	0.97	1.09	0.99	0.83	0.80	0.86	0.99	1.37	0.14
513	c	1.00	1.28	1.10	1.12	1.03	0.94	0.99	0.96	0.82	0.80	0.95	0.85	0.80	0.71	0.88	1.06	1.27	0.10
514	c	1.00	1.16	1.15	1.02	1.01	0.98	1.03	1.01	0.63	0.70	0.77	0.70	0.65	0.48	0.72	0.87	1.20	0.08
515	c	1.00	1.07	1.05	0.98	0.86	0.85	0.89	0.79	0.70	0.81	0.77	0.76	0.66	0.40	0.70	0.85	1.19	0.08
516	a	1.00	1.07	1.11	1.03	0.68	0.61	0.65	0.67	0.63	0.54	0.59	0.53	0.62	0.46	0.63	0.79	1.53	0.19
517	c	1.00	1.40	1.42	1.04	1.05	1.01	1.09	1.25	0.97	1.08	1.18	1.13	1.38	1.33	1.21	1.40	0.87	-0.06
518	c	1.00	1.15	1.36	1.10	1.11	1.01	1.10	1.06	0.81	0.88	0.97	0.87	1.79	1.56	1.55	1.55	0.81	-0.09
519	c	1.00	1.09	1.13	1.13	0.99	0.99	1.00	0.91	0.68	0.73	0.78	0.72	0.87	0.72	0.87	0.93	1.01	0.01
520	a	1.00	0.98	1.08	1.11	0.92	0.88	0.92	0.87	0.92	0.66	0.70	0.69	0.83	0.74	0.88	0.94	1.51	0.18
521	c	1.00	1.08	1.13	1.21	1.13	1.12	1.07	1.22	0.98	1.05	0.96	1.04	1.54	1.78	1.47	1.41	0.87	-0.06

522	c	1.00	0.92	1.01	1.14	0.93	0.97	0.85	0.93	1.13	0.79	0.89	0.97	2.47	3.43	2.18	1.87	0.89	-0.05
523	t	1.00	0.85	0.94	1.09	0.73	0.80	0.78	0.72	0.69	0.66	0.64	0.71	1.72	2.59	1.61	1.53	0.86	-0.07
524	a	1.00	0.93	1.07	1.27	0.84	0.75	0.89	0.81	1.11	0.58	0.64	0.65	0.70	1.03	0.79	0.85	2.10	0.32
525	c	1.00	0.85	0.83	1.07	0.93	0.97	1.02	1.03	2.78	2.50	2.11	2.32	1.51	2.53	1.65	1.35	1.07	0.03
526	a	1.00	0.75	0.81	0.96	0.58	0.55	0.52	0.51	0.90	0.40	0.40	0.51	0.42	0.57	0.45	0.54	2.32	0.36
527	t	1.00	0.59	0.72	0.88	0.75	0.83	0.88	0.81	2.59	2.17	1.67	1.97	0.97	1.48	0.89	0.81	1.10	0.04
528	c	1.00	0.57	0.80	0.71	0.56	0.70	0.67	0.66	1.40	1.41	0.78	0.98	1.38	2.30	0.85	0.92	0.95	-0.02
529	a	1.00	0.67	0.98	0.79	0.52	0.52	0.52	0.50	0.84	0.49	0.36	0.50	0.62	0.73	0.55	0.60	1.63	0.21
530	c	1.00	0.67	0.94	0.61	0.55	0.69	0.59	0.70	1.33	1.10	0.81	1.01	1.27	1.91	0.70	0.89	0.92	-0.04
531	t	1.00	0.68	1.13	0.75	0.54	0.59	0.68	0.61	0.52	0.41	0.38	0.49	0.86	1.01	0.58	0.66	0.83	-0.08
532	c	1.00	0.69	1.04	0.54	0.48	0.64	0.56	0.65	0.58	0.42	0.45	0.51	2.29	3.28	1.05	1.38	0.68	-0.17
533	t	1.00	0.77	1.08	0.74	0.55	0.78	0.66	0.67	0.65	0.50	0.50	0.60	1.00	1.42	0.58	0.95	1.02	0.01
534	c	1.00	0.87	1.13	0.92	0.87	0.93	0.76	0.81	0.59	0.44	0.52	0.69	2.26	4.15	1.64	2.12	0.80	-0.10
535	t	1.00	0.76	0.72	0.77	0.53	0.45	0.46	0.44	0.53	0.58	0.42	0.45	1.20	2.85	1.64	1.56	1.53	0.18
536	g	1.00	0.79	0.69	0.81	0.55	0.66	0.35	0.29	0.24	0.29	0.26	0.34	0.34	0.85	0.52	0.57	1.19	0.08
537	a	1.00	0.94	0.62	0.80	0.40	0.18	0.82	1.02	0.30	0.27	0.18	0.26	0.46	0.96	0.44	0.46	1.12	0.05
538	g	1.00	0.95	0.87	1.24	1.64	2.86	2.58	2.04	0.18	0.32	0.35	0.62	0.31	0.83	0.49	0.59	0.55	-0.26
539	g	-	-	-	-	-	-	-	-	-	-	-	-	-	-	-	-	-	-
540	a	-	-	-	-	-	-	-	-	-	-	-	-	-	-	-	-	-	-
541	c	-	-	-	-	-	-	-	-	-	-	-	-	-	-	-	-	-	-
542	t	-	-	-	-	-	-	-	-	-	-	-	-	-	-	-	-	-	-
543	a	-	-	-	-	-	-	-	-	-	-	-	-	-	-	-	-	-	-
544	a	-	-	-	-	-	-	-	-	-	-	-	-	-	-	-	-	-	-
545	a	-	-	-	-	-	-	-	-	-	-	-	-	-	-	-	-	-	-
546	a	-	-	-	-	-	-	-	-	-	-	-	-	-	-	-	-	-	-
547	c	-	-	-	-	-	-	-	-	-	-	-	-	-	-	-	-	-	-
548	t	-	-	-	-	-	-	-	-	-	-	-	-	-	-	-	-	-	-
549	g	-	-	-	-	-	-	-	-	-	-	-	-	-	-	-	-	-	-
550	c	-	-	-	-	-	-	-	-	-	-	-	-	-	-	-	-	-	-
551	a	-	-	-	-	-	-	-	-	-	-	-	-	-	-	-	-	-	-
552	c	-	-	-	-	-	-	-	-	-	-	-	-	-	-	-	-	-	-
553	c	-	-	-	-	-	-	-	-	-	-	-	-	-	-	-	-	-	-
554	t	-	-	-	-	-	-	-	-	-	-	-	-	-	-	-	-	-	-
555	t	-	-	-	-	-	-	-	-	-	-	-	-	-	-	-	-	-	-
556	c	-	-	-	-	-	-	-	-	-	-	-	-	-	-	-	-	-	-
557	c	-	-	-	-	-	-	-	-	-	-	-	-	-	-	-	-	-	-
558	t	-	-	-	-	-	-	-	-	-	-	-	-	-	-	-	-	-	-
559	t	-	-	-	-	-	-	-	-	-	-	-	-	-	-	-	-	-	-
560	c	-	-	-	-	-	-	-	-	-	-	-	-	-	-	-	-	-	-
561	t	-	-	-	-	-	-	-	-	-	-	-	-	-	-	-	-	-	-
562	c	-	-	-	-	-	-	-	-	-	-	-	-	-	-	-	-	-	-
563	t	-	-	-	-	-	-	-	-	-	-	-	-	-	-	-	-	-	-
564	c	-	-	-	-	-	-	-	-	-	-	-	-	-	-	-	-	-	-
565	t	-	-	-	-	-	-	-	-	-	-	-	-	-	-	-	-	-	-
566	c	-	-	-	-	-	-	-	-	-	-	-	-	-	-	-	-	-	-
567	c	-	-	-	-	-	-	-	-	-	-	-	-	-	-	-	-	-	-
568	c	-	-	-	-	-	-	-	-	-	-	-	-	-	-	-	-	-	-
569	t	-	-	-	-	-	-	-	-	-	-	-	-	-	-	-	-	-	-
570	g	-	-	-	-	-	-	-	-	-	-	-	-	-	-	-	-	-	-
571	c	-	-	-	-	-	-	-	-	-	-	-	-	-	-	-	-	-	-
572	a	-	-	-	-	-	-	-	-	-	-	-	-	-	-	-	-	-	-
573	g	-	-	-	-	-	-	-	-	-	-	-	-	-	-	-	-	-	-
574	G	-	-	-	-	-	-	-	-	-	-	-	-	-	-	-	-	-	-
575	A	-	-	-	-	-	-	-	-	-	-	-	-	-	-	-	-	-	-
576	T	-	-	-	-	-	-	-	-	-	-	-	-	-	-	-	-	-	-
577	A	-	-	-	-	-	-	-	-	-	-	-	-	-	-	-	-	-	-
578	C	-	-	-	-	-	-	-	-	-	-	-	-	-	-	-	-	-	-
579	T	-	-	-	-	-	-	-	-	-	-	-	-	-	-	-	-	-	-
580	T	-	-	-	-	-	-	-	-	-	-	-	-	-	-	-	-	-	-
581	T	-	-	-	-	-	-	-	-	-	-	-	-	-	-	-	-	-	-
582	T	-	-	-	-	-	-	-	-	-	-	-	-	-	-	-	-	-	-
583	G	-	-	-	-	-	-	-	-	-	-	-	-	-	-	-	-	-	-
584	T	-	-	-	-	-	-	-	-	-	-	-	-	-	-	-	-	-	-
585	G	-	-	-	-	-	-	-	-	-	-	-	-	-	-	-	-	-	-
586	G	-	-	-	-	-	-	-	-	-	-	-	-	-	-	-	-	-	-
587	A	-	-	-	-	-	-	-	-	-	-	-	-	-	-	-	-	-	-
588	A	-	-	-	-	-	-	-	-	-	-	-	-	-	-	-	-	-	-
589	C	-	-	-	-	-	-	-	-	-	-	-	-	-	-	-	-	-	-
590	T	-	-	-	-	-	-	-	-	-	-	-	-	-	-	-	-	-	-
591	C	-	-	-	-	-	-	-	-	-	-	-	-	-	-	-	-	-	-
592	T	-	-	-	-	-	-	-	-	-	-	-	-	-	-	-	-	-	-
593	A	-	-	-	-	-	-	-	-	-	-	-	-	-	-	-	-	-	-
594	T	-	-	-	-	-	-	-	-	-	-	-	-	-	-	-	-	-	-
595	G	1.00	1.20	1.10	1.36	2.57	2.48	2.57	1.82	1.70	1.43	1.97	1.84	1.02	0.65	1.16	0.88	0.80	-0.10
596	G	1.00	1.27	0.94	1.07	2.43	2.35	2.20	1.72	0.49	0.78	0.45	0.74	0.28	0.14	0.18	0.12	0.27	-0.57
597	G	1.00	1.27	1.08	0.88	5.61	6.98	3.74	3.20	1.07	0.97	1.42	0.86	0.75	0.36	0.58	0.63	1.03	0.01

598	A	1.00	0.72	1.01	1.10	0.83	0.94	3.27	1.18	1.41	1.57	1.58	2.05	2.68	1.52	3.28	3.62	0.93	-0.03
599	A	1.00	1.01	1.21	1.27	1.24	1.12	1.48	1.05	1.25	1.50	1.57	2.06	0.71	0.11	2.11	2.39	2.03	0.31
600	C	1.00	1.08	0.44	0.15	0.57	0.36	0.09	0.07	0.76	0.14	0.35	0.55	0.31	0.12	0.26	0.09	0.38	-0.42
601	A	1.00	0.78	3.68	6.07	1.09	1.35	2.38	1.44	1.92	2.26	2.27	0.00	3.06	1.73	2.12	3.41		
602	A	1.00	1.09	1.53	1.61	0.79	0.68	0.65	0.67	1.65	1.38	1.39	1.11	1.45	0.76	1.15	1.46	1.49	0.17
603	T	1.00	1.00	0.81	1.38	0.66	0.52	0.41	0.51	1.36	1.42	1.33	1.67	0.64	0.79	1.59	1.42	1.79	0.25
604	G	1.00	0.74	0.72	0.91	0.41	0.44	0.37	0.31	0.94	0.74	0.82	0.71	0.75	0.43	0.76	0.77	1.36	0.13
605	C	1.00	1.03	0.89	0.92	4.07	4.97	3.89	2.00	1.79	1.13	2.56	1.01	2.03	0.95	0.93	1.14	1.00	0.00
606	A	1.00	1.48	1.43	1.42	0.99	0.62	0.54	0.38	1.13	0.47	0.36	0.55	0.54	0.22	0.64	0.72	2.72	0.44
607	G	1.00	0.38	1.63	1.12	0.31	0.28	0.19	0.39	1.50	1.18	1.61	1.28	2.42	1.72	2.32	2.36	1.14	0.06
608	C	1.00	0.63	0.03	0.33	2.60	1.98	3.08	1.76	0.47	0.87	1.27	0.78	0.93	0.69	0.94	1.19	0.78	-0.11
609	A	1.00	1.20	0.90	0.88	0.74	0.45	0.28	0.31	1.44	0.50	0.47	0.65	0.82	0.41	0.76	0.79	2.13	0.33
610	G	1.00	0.93	0.99	1.70	2.31	3.11	1.18	1.24	0.83	1.26	1.35	1.21	2.24	1.89	2.82	2.68	0.82	-0.09
611	C	1.00	0.79	1.24	1.09	2.01	1.22	4.01	2.53	0.38	0.86	1.29	0.81	0.66	2.95	1.09	0.91	0.65	-0.19
612	C	1.00	1.27	1.18	1.24	0.98	0.79	0.82	0.68	1.08	0.85	0.97	0.95	4.27	1.55	3.75	3.95	1.05	0.02
613	G	1.00	0.98	0.95	0.99	0.61	0.41	0.51	0.40	0.86	0.78	0.77	0.92	2.67	1.98	2.97	2.84	0.98	-0.01
614	A	1.00	0.93	0.96	1.05	3.06	3.08	3.53	2.36	0.49	0.69	2.94	0.89	3.53	3.28	3.95	3.35	0.52	-0.29
615	G	1.00	1.07	1.03	1.00	1.16	1.01	0.81	1.60	2.41	3.27	1.97	4.22	1.40	1.29	1.45	1.26	0.51	-0.29
616	A	1.00	1.05	1.03	1.08	6.80	7.40	8.65	6.70	0.90	0.92	1.26	1.31	0.76	0.60	0.84	0.95	0.86	-0.07
617	G	1.00	1.12	0.82	1.02	0.76	1.01	0.52	0.62	1.98	2.67	2.85	2.85	0.60	0.48	0.69	0.70	0.81	-0.09
618	C	1.00	1.11	1.32	1.33	2.81	2.93	2.97	2.22	0.74	0.64	0.79	0.65	0.68	0.36	0.83	1.01	1.70	0.23
619	C	1.00	1.19	1.14	1.25	0.67	0.63	0.67	0.50	0.75	0.67	0.68	0.65	1.04	0.74	1.11	1.11	1.22	0.09
620	G	1.00	1.09	1.03	0.96	0.55	0.53	0.63	0.47	0.69	0.71	0.63	0.62	0.74	0.46	0.80	0.85	1.27	0.10
621	A	1.00	1.13	0.97	1.15	2.99	3.58	3.56	3.49	0.77	0.84	1.11	0.95	0.99	0.82	1.19	1.22	0.99	0.00
622	A	1.00	1.14	1.09	1.21	0.72	0.79	0.74	0.90	2.40	3.55	3.21	3.57	0.74	0.60	0.79	1.00	0.91	-0.04
623	A	1.00	1.18	1.02	1.15	0.63	0.52	0.77	0.83	1.95	2.67	3.09	3.07	0.75	0.64	0.86	0.97	0.82	-0.09
624	G	1.00	1.08	0.96	1.00	0.64	0.58	0.77	0.92	3.40	5.13	5.54	6.29	0.66	0.61	0.77	0.80	0.65	-0.18
625	G	1.00	1.06	1.01	0.98	2.83	3.01	2.50	1.99	0.86	1.13	1.31	0.94	0.68	0.59	0.77	0.94	1.28	0.11
626	G	1.00	1.17	1.11	1.06	1.64	1.80	1.72	1.27	0.75	0.74	0.76	0.68	0.85	0.55	0.87	1.03	1.34	0.13
627	C	1.00	1.20	1.19	1.14	1.14	1.21	1.17	0.85	0.73	0.75	0.80	0.74	0.62	0.41	0.74	0.93	1.49	0.17
628	C	1.00	1.17	1.14	1.12	0.71	0.77	0.89	0.58	0.70	0.74	0.66	0.70	1.27	0.96	1.30	1.35	1.07	0.03
629	A	1.00	1.41	1.26	1.20	0.84	0.60	0.65	0.50	1.24	0.67	0.60	0.63	2.33	2.05	2.39	2.31	1.93	0.29
630	G	1.00	1.12	1.12	1.13	0.87	0.77	0.88	0.80	0.70	0.87	0.79	0.85	2.87	2.64	2.86	2.52	0.72	-0.14
631	G	1.00	1.18	1.09	0.98	0.79	0.67	0.89	0.78	0.69	0.87	0.68	0.80	0.98	1.00	1.00	0.97	0.85	-0.07
632	A	1.00	1.26	1.06	1.19	3.07	4.32	3.80	2.78	0.87	0.92	0.90	1.16	0.55	0.49	0.72	0.83	1.13	0.05
633	A	1.00	1.34	0.96	1.10	0.74	0.74	0.77	0.79	3.58	4.55	3.96	4.36	1.19	1.08	1.41	1.51	1.05	0.02
634	C	1.00	1.37	0.99	1.13	0.91	1.45	1.35	1.10	4.19	4.78	4.57	5.25	2.13	2.08	2.36	2.25	0.85	-0.07
635	G	1.00	1.19	0.90	0.93	0.77	1.04	0.98	0.88	2.12	2.64	2.04	2.28	0.92	0.94	0.93	0.92	0.94	-0.03
636	C	1.00	1.30	0.98	1.07	7.91	9.87	7.91	6.02	1.61	1.83	1.52	1.59	1.00	1.40	1.13	1.13	1.14	0.06
637	T	1.00	1.23	0.98	1.07	0.61	0.45	0.54	0.45	2.37	2.85	2.26	2.45	0.65	0.50	0.70	0.76	1.13	0.05
638	T	1.00	1.25	1.03	1.16	0.78	0.68	0.74	0.81	3.02	3.84	2.92	3.14	1.54	2.28	1.99	2.10	1.31	0.12
639	C	1.00	1.35	1.12	1.27	0.95	0.88	0.92	1.08	3.70	4.81	3.81	4.04	2.47	3.97	2.43	2.30	0.85	-0.07
640	A	1.00	1.10	1.02	0.99	0.75	0.77	0.58	0.45	1.36	1.10	0.94	1.03	0.67	0.67	0.69	0.74	1.46	0.16
641	A	1.00	1.13	1.00	1.12	0.71	0.80	0.89	1.04	2.72	3.84	3.07	3.74	1.15	1.73	1.20	1.21	0.77	-0.11
642	C	1.00	1.20	0.98	1.58	0.79	0.70	0.98	1.15	4.68	6.06	5.08	5.60	0.89	0.98	0.99	1.10	1.03	0.01
643	C	1.00	1.14	1.15	0.99	1.06	0.99	1.06	1.15	1.21	1.24	1.23	1.31	1.03	1.06	1.16	1.06	0.95	-0.02
644	G	1.00	0.96	0.95	0.95	0.65	0.79	0.72	0.94	1.05	1.35	1.12	1.11	0.70	0.67	0.73	0.72	0.97	-0.01
645	C	1.00	0.91	0.93	1.05	3.54	4.23	3.83	2.46	0.61	0.79	0.57	0.70	0.89	0.91	0.80	0.97	0.94	-0.03
646	T	1.00	1.08	1.14	1.25	0.72	0.51	0.50	0.63	0.94	0.91	0.69	0.94	0.91	0.90	0.90	1.05	1.16	0.06
647	G	1.00	0.93	0.87	0.73	0.53	0.60	0.64	1.04	0.60	0.73	0.65	0.71	0.64	0.60	0.69	0.66	0.86	-0.06
648	G	1.00	0.94	0.97	1.03	0.74	0.69	0.77	0.64	0.71	0.82	0.60	0.74	0.69	0.61	0.78	0.77	1.06	0.03
649	T	1.00	0.93	0.96	0.98	0.70	0.73	0.74	0.78	0.56	0.70	0.61	0.65	0.49	0.36	0.56	0.61	1.07	0.03
650	T	1.00	0.92	0.94	0.99	0.62	0.64	0.71	0.77	0.53	0.68	0.59	0.62	0.66	0.71	0.67	0.64	0.84	-0.07
651	C	1.00	1.01	1.02	0.98	0.80	0.83	0.95	1.07	0.85	0.83	0.68	0.82	1.76	2.37	1.83	1.78	1.04	0.02
652	C	1.00	1.02	1.13	1.14	0.91	0.81	0.85	0.84	0.73	0.78	0.75	0.73	1.84	2.25	1.72	1.60	0.87	-0.06
653	T	1.00	0.98	0.96	0.94	0.60	0.62	0.65	0.68	0.58	0.61	0.59	0.57	0.62	0.51	0.64	0.65	1.07	0.03
654	G	1.00	1.10	1.12	1.15	1.61	1.52	1.41	1.68	0.68	0.79	0.72	0.78	0.77	0.64	0.72	0.89	1.00	0.00
655	A	1.00	1.00	0.98	1.10	0.75	0.68	0.70	1.01	0.92	1.20	1.10	1.18	1.14	1.72	1.04	0.99	0.67	-0.17
656	C	1.00	1.02	0.98	0.90	0.53	0.38	0.35	0.61	0.81	0.72	0.71	0.67	1.14	2.10	1.06	1.01	1.08	0.03
657	G	1.00	0.80	0.92	0.84	0.74	0.86	0.92	0.96	0.47	0.63	0.61	0.71	1.53	2.63	1.37	1.17	0.50	-0.30
658	G	1.00	0.98	1.19	1.18	1.01	0.98	1.16	0.98	0.74	0.68	0.85	0.68	0.75	0.55	0.77	0.88	1.27	0.10
659	G	1.00	0.83	0.97	0.87	0.82	0.84	0.91	0.71	0.52	0.62	0.59	0.59	0.74	0.98	0.72	0.60	0.73	-0.14
660	C	1.00	0.87	1.05	1.05	0.63	0.48	0.43	0.39	0.98	0.49	0.54	0.45	0.44	0.53	0.50	0.54	2.70	0.43
661	A	1.00	0.70	0.75	0.67	0.57	0.50	0.49	0.78	0.59	0.62	0.78	0.62	1.83	2.44	1.54	1.32	0.69	-0.16
662	T	1.00	0.80	0.91	0.80	0.62	0.58	0.78	2.23	0.71	0.87	1.10	0.95	0.90	0.79	0.68	0.75	0.62	-0.21

Average	-0.001073123
Standard Deviation	0.199782384

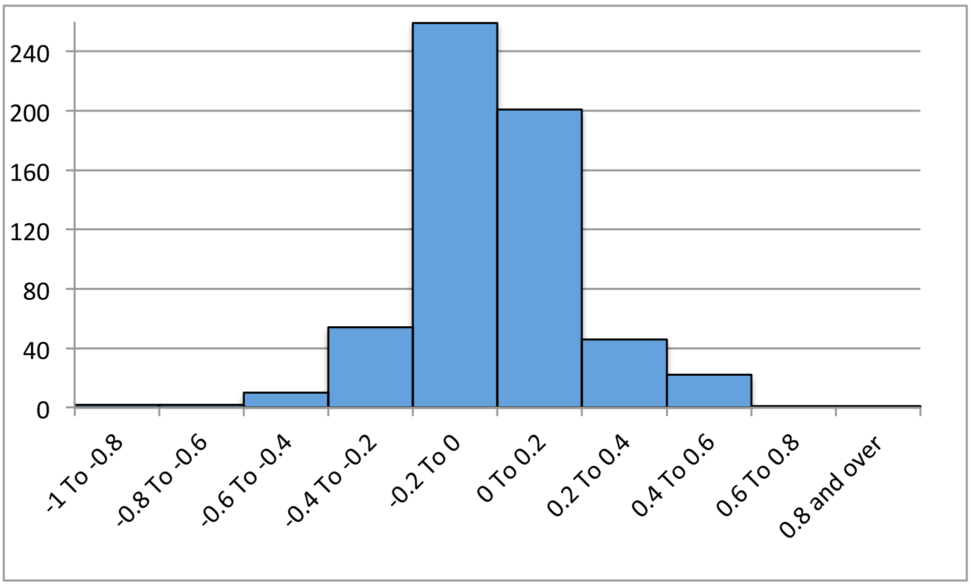

Index frequency:

-1 To -0.8	2
-0.8 To -0.6	2
-0.6 To -0.4	10
-0.4 To -0.2	54
-0.2 To 0	259
0 To 0.2	201
0.2 To 0.4	46
0.4 To 0.6	22
0.6 To 0.8	1
0.8 and over	1
